# The relevance of mitochondrial DNA variants fluctuation during reprogramming and neuronal differentiation of human iPSCs

**DOI:** 10.1016/j.stemcr.2021.06.016

**Published:** 2021-07-29

**Authors:** Flavia Palombo, Camille Peron, Leonardo Caporali, Angelo Iannielli, Alessandra Maresca, Ivano Di Meo, Claudio Fiorini, Alice Segnali, Francesca L. Sciacca, Ambra Rizzo, Sonia Levi, Anu Suomalainen, Alessandro Prigione, Vania Broccoli, Valerio Carelli, Valeria Tiranti

**Affiliations:** 1 IRCCS Istituto delle Scienze Neurologiche di Bologna, Programma di Neurogenetica, Bologna 40139, Italy; 2 Fondazione IRCCS Istituto Neurologico Carlo Besta, Milan 20133, Italy; 3 Division of Neuroscience, San Raffaele Scientific Institute, Milan 20132, Italy; 4 Department of Biomedical and NeuroMotor Sciences (DIBINEM), University of Bologna, Bologna 40123, Italy; 5 Vita-Salute San Raffaele University, Milan 20132, Italy; 6 Stem Cell and Metabolism Research Program Unit, Faculty of Medicine, University of Helsinki, Helsinki 00014, Finland; 7 Neuroscience Institute, HiLife, University of Helsinki, Helsinki 00014, Finland; 8 HUSLab, Helsinki University Hospital, Helsinki 00014, Finland; 9 Department of General Pediatrics, Neonatology and Pediatric Cardiology, Duesseldorf University Hospital, Medical Faculty, Heinrich Heine University, Duesseldorf 40225, Germany; 10 National Research Council (CNR), Institute of Neuroscience, Milan 20132, Italy

**Keywords:** human iPSCs, neuronal precursor cells, mtDNA deep sequencing, iPSCs quality control

## Abstract

The generation of inducible pluripotent stem cells (iPSCs) is a revolutionary technique allowing production of pluripotent patient-specific cell lines used for disease modeling, drug screening, and cell therapy. Integrity of nuclear DNA (nDNA) is mandatory to allow iPSCs utilization, while quality control of mitochondrial DNA (mtDNA) is rarely included in the iPSCs validation process. In this study, we performed mtDNA deep sequencing during the transition from parental fibroblasts to reprogrammed iPSC and to differentiated neuronal precursor cells (NPCs) obtained from controls and patients affected by mitochondrial disorders. At each step, mtDNA variants, including those potentially pathogenic, fluctuate between emerging and disappearing, and some having functional implications. We strongly recommend including mtDNA analysis as an unavoidable assay to obtain fully certified usable iPSCs and NPCs.

## Highlights

• mtDNA deep sequencing is mandatory in quality control of iPSCs
• mtDNA variants fluctuate at each step from fibroblasts/PBMC, to iPSCs and NPCs
• mtDNA variants greatly affect iPSC phenotype, reflecting their healthiness
• Results could be misinterpreted if mtDNA variants presence has not been assessed

## Graphical abstract

**Figure:**
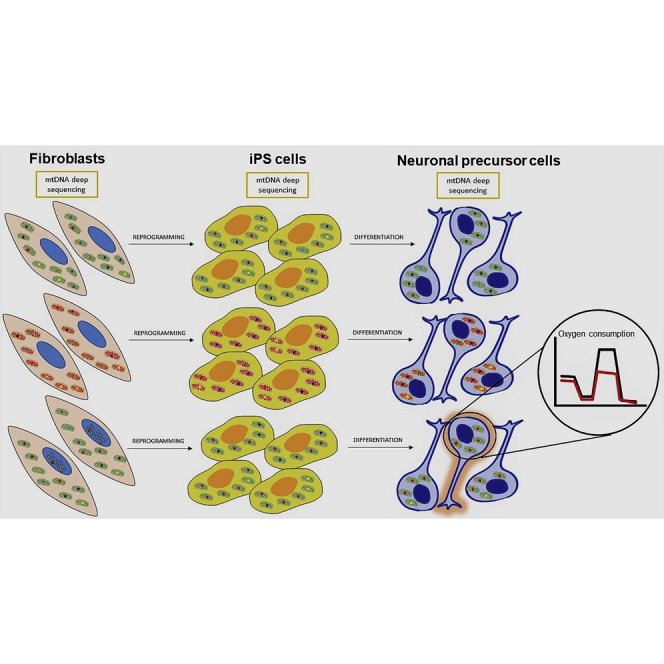

## Teaser

Tiranti and colleagues observed that quality control of iPSCs rarely includes the analysis of mitochondrial DNA (mtDNA), a small circular molecule present in the mitochondria. However, mtDNA variants greatly affect the final phenotype of iPSCs. They highlighted that iPSC-derived neuronal precursors could also present mtDNA variants and underlined the paramount importance of screening mtDNA.

## Introduction

The exponential increase of applications of induced pluripotent stem cells (iPSCs) includes generation of differentiated cells, development of organoids for investigations of disease mechanisms and drug discovery (Shi et al., 2017), and their clinical use for therapeutic purposes in humans (Barker et al., 2017). This poses specific questions on their quality control, and there are concerns about age-related burden of somatic mutations (Kang et al., 2016; Lo Sardo et al., 2017), lineage-specific epigenetic memory affecting the methylation pattern (Nashun et al., 2015), and pre-existing or reprogramming-related occurrence of tumorigenic mutations (Ben-David and Benvenisty, 2011; Gore et al., 2011). Such concerns have recently hampered and currently limit the therapeutic use of autologous iPSCs (Garber, 2015).

One area of genetic variability affecting the quality of iPSCs is mitochondrial DNA (mtDNA), a small, circular, multicopy, double-stranded molecule of DNA within mitochondria, which has a mutagenesis rate much higher than nuclear DNA (Gustafsson et al., 2016; Wallace, 2015). The mtDNA hosts hundreds of pathogenic mutations causing a vast variety of clinical phenotypes (La Morgia et al., 2020) characterized by defective oxidative phosphorylation (OXPHOS), increased oxidative stress, calcium mishandling, propensity to apoptosis, altered organelle dynamics, and removal by autophagy (Nunnari and Suomalainen, 2012). Furthermore, mtDNA genetics follows peculiar rules: maternal inheritance, homo-hetero-polyplasmy, mitotic segregation and threshold effect, germline bottleneck, and individual cell clonal expansion of mutant molecules. All these mtDNA features are reflected in higher-order complexities when it comes to global function of individual cells, including iPSCs (Xu et al., 2013). Of particular relevance is the potential functional reflection on iPSCs of mtDNA sequence diversity characterizing human populations (Gómez-Durán et al., 2010; Strobbe et al., 2018).

A few studies clearly highlighted the major impact that mtDNA mutagenesis may have on iPSC quality (Kang et al., 2016; Lorenz and Prigione, 2016; Perales-Clemente et al., 2016; Prigione et al., 2011; Zambelli et al., 2018). Critical issues are the clonal expansion in single cells of private heteroplasmic mtDNA variants as part of the so-called universal heteroplasmy (Payne et al., 2013), the possible *de novo* mtDNA mutagenesis occurring during cycling of the parental cell line, the possible mtDNA mutagenesis related to cell reprogramming, and the bottleneck effects and genetic drifts due to the mtDNA copy number reduction characterizing iPSCs (Bukowiecki et al., 2014; Xu et al., 2013). All these possibilities remain open questions, and previous studies have reached contradictory results. The introduction of deep next-generation sequencing (NGS) has lowered the detection limit of heteroplasmy to 0.2%, and different studies demonstrated that low-level heteroplasmy (down to 0.1%) may be transmitted and maintained within families (Giuliani et al., 2014; Guo et al., 2013).

We present our own analysis of iPSCs generated from fibroblasts and peripheral blood mononuclear cells (PBMCs) and, for the first time, of neuronal precursor cells (NPCs). We performed mtDNA deep sequencing in fibroblasts/iPSCs/NPCs obtained from controls and two classes of patients: a mitochondrial group carrying known homoplasmic or heteroplasmic mtDNA mutations and a nuclear group carrying mutations in nuclear genes coding for mitochondrial proteins.

## Results

### Deep sequencing of mtDNA

We performed mtDNA deep sequencing in 17 fibroblast cell lines, one PBMC cell line, 35 iPSCs clones, and 16 NPCs, belonging to the three categories: controls (C1 to C5) (Figure S1A), mitochondrial (M1 to M6) (Figure S1B), and nuclear (N1 to N7) patients (Figure S1C) (Table 1). A median coverage of 14,978X (±4,731X), 7,138X (±3,269X), and 6,172X (±2,551X) was respectively achieved in fibroblasts/PBMCs, iPSCs, and NPCs (Table S1). In some cases, we observed a coverage reduction for certain mtDNA regions and we checked for the presence of macrodeletions by analyzing the sequencing data with the MitoSAlt tool (https://sourceforge.net/projects/mitosalt/) (Basu et al., 2020). No macrodeletions were observed with heteroplasmy levels higher than 1%. This result was further supported by the analysis with digital droplet PCR, on the same cell lines carrying *OPA1* mutations here investigated and previously published (Iannielli et al., 2018).

**Table 1:** Features of subjects in control, mitochondrial, and nuclear groups

Patient Age	Haplogroup	Phenotype (#MIM)	Gene	Nt change	AA change	Reprogram system	Starting material	iPSC clones	NPC clones	CGH/Karyotype iPSC clones	Reference
**Control group**											

C1 34	J1c2	none	none	none	none	Sendai virus	fibroblasts	19M	0	0.4-Mb duplication in 18p11.22	
								38M	0	normal	
								44M	1	normal	
C2 54	H4a1a	none	none	none	none	Sendai virus	fibroblasts	#37	1	normal	
								#68	1	normal	
C3 32	H1	none	none	none	none	Sendai virus	fibroblasts	#7	0	1.5-Mb deletion in 16q23.3	
C4 54	H4a1a	none	none	none	none	Sendai virus	fibroblasts	#123	0	normal	
								#130	0	normal	
C5 39	D1g	none	none	none	none	Sendai virus	PBMCs	#105	0	normal	

**Mitochondrial group**											

M1 26	J1c4	LHON (#535000)	MT-ND4	m.11778G>A homoplasmic	p.R340H	Sendai virus	fibroblasts	#20	1	normal	
								#32	0	normal	
M2 19	H1at1	LHON (#535000)	MT-ND1	m.3460G>A homoplasmic	p.A52T	Sendai virus	fibroblasts	15M	1	60.6-Mb duplication in 1p35.3p22.2	
								33M	0	normal	Peron et al., (2020)
M3 40	H7	MELAS (#540000)	MT-TL1	m.3243A>G heteroplasmic	NA	retroviral transduction	fibroblasts	#A	0	normal	Hämäläinen et al., (2013)
								#B	0	normal	
								#L	0	normal	
								#N	0	normal	
M4 80	H1b	NARP (#551500)	MT-ATP6	m.9185T>C homoplasmic	p.L220P	episomal plasmid	fibroblasts	A1	1	normal	Auré et al. (2013) Lorenz et al., (2017)
M5 47	H1b	NARP (#551500)	MT-ATP6	m.9185T>C homoplasmic	p.L220P	retrovirus	fibroblasts	A2	1	normal	
M6 20	H1b	NARP (#551500)	MT-ATP6	m.9185T>C homoplasmic	p.L220P	retrovirus	fibroblasts	A3	1	normal	

**Nuclear group**											

N1 12	U5a1a1	PKAN (#234200)	PANK2	c.569insA homozygous	p.Y190X	Sendai virus	fibroblasts	1535	1	normal	Orellana et al., (2016)
N2 20	U4b1a	DOA (#165500)	OPA1	c.1334 G>A heterozygous	p.R445H	Sendai virus	fibroblasts	13M	1	normal	
								20M	0	normal	
N3 27	H13a1a1	DOA (#165500)	OPA1	c.1334 G>A heterozygous	p.R445H	Sendai virus	fibroblasts	#202	0	normal	
								#205	0	normal	
N4 69	HV0b	DOA (#165500)	OPA1	c.1462A>G heterozygous	p.G488R	Sendai virus	fibroblasts	#12	1	normal	Iannielli et al., (2018)
								#18	1	normal	
N5 70	H1aq	DOA (#165500)	OPA1	c.1484C>T heterozygous	p.A495V	Sendai virus	fibroblasts	#72	1	normal	
								#75	1	normal	
N6 57	H1ab1	CPEO (#203700)	POLG	c.1943C>G homozygous	p.P648R	Sendai virus	fibroblasts	#2	0	normal	
								#3	0	normal	
								#4	1	normal	
								#6	0	normal	
								#8	1	normal	
N7 75	J1c15	CPEO (#609286)	TWNK	c.907C>T heterozygous	p.R303W	Sendai virus	fibroblasts	#34	0	normal	

MIM, Online Mendelian Inheritance in Man; Nt, nucleotide; AA, amino acid; CPEO, chronic progressive external ophtalmoplegia

Variants were analyzed with PhyloTree (https://www.phylotree.org/tree/index.htm) to reconstruct the haplogroup of each cell line and the full consistency of haplotypes between parental fibroblasts, iPSCs derived clones, and correlated NPCs (Table 1). All haplogroups were common to the population of European ancestry (H, J, U), except one control cell line (C5) carrying the D1g haplotype. We analyzed only private single nucleotide mtDNA variants, not belonging to the specific haplogroup. We traced their segregation during reprogramming and differentiation to assess the change in heteroplasmy fraction (HF) and screened for possible novel damaging variants.

### Impact of age, reprogramming methods, and mitochondrial haplotype on abundance of mtDNA variants in fibroblasts, iPSCs, and NPCs

**Figure 1:**
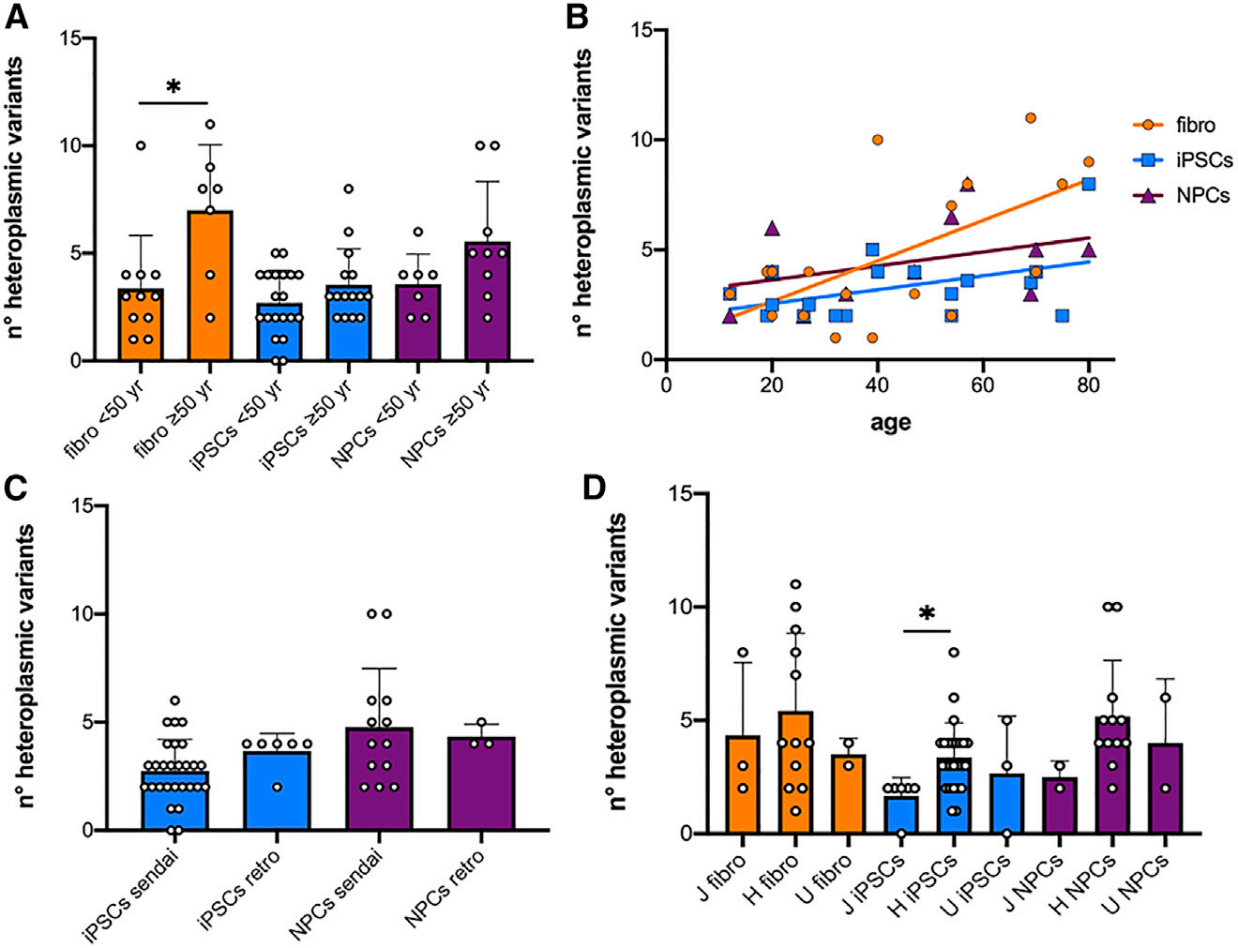
Effect of age, reprogramming methods, and mitochondrial haplotype on the number of mtDNA heteroplasmic variants in fibroblasts, iPSCs, and NPCs (A) Elderly subjects presented higher number of heteroplasmic variants in fibroblasts (p = 0.0132), but not in iPSCs (p = 0.135) and NPCs (p = 0.10). (B) The linear correlation between ages and the accumulation of heteroplasmic variants was statistically significant only in fibroblasts (adjusted *R*-squared = 0.376, p = 0.0068). Adjusted *R*-squared = 0.196, p = 0.065 in iPSCs; adjusted *R*-squared = 0.151, p = 0.236 in NPCs. (C) Number of heteroplasmic variants was independent of the reprogramming method in iPSCs (p = 0.08) and NPCs (p = 0.97). (D) Haplotype J had significantly fewer variants in iPSCs (p = 0.031). Number of variants in fibroblasts (p = 0.796) and NPCs (p = 0.241) was not related to a specific haplotype.

In our study, the age range of subjects was wide (12–80 years) and we investigated the impact of age on the number of mtDNA variants. The average number of variants in fibroblasts from older subjects (≥50 years old) was significantly higher compared with younger subjects (Figure 1A). Moreover, there was a linear correlation (Figure 1B) between ages and somatic mtDNA variability accumulated in fibroblasts. In contrast, we did not observe a greater number of mtDNA variants (Figure 1A) either in the iPSCs or in the NPCs in the older group. Similarly, the age-related variants burden was progressively lesser and gradually dropped in each cell stage (Figure 1B).

We here employed three reprogramming procedures: Sendai virus in 14 cell lines, retroviral transduction in three lines, and an episomal plasmid in the remaining one (M4, Table 1). We did not observe a significant effect of a specific reprogramming method on the number of mtDNA heteroplasmic variants either in iPSCs or in NPCs (Figure 1C).

We also evaluated the effect of mitochondrial haplotypes, specifically J, H, and U, on the burden of mtDNA variants, excluding the D1g haplotype, present in a single cell line. The number of variants in fibroblasts and NPCs was not related to a specific haplotype, while haplotype J turned out to have significantly fewer variants in iPSCs (Figure 1D).

### mtDNA heteroplasmy and variants segregations from parental fibroblasts to iPSCs

**Figure 2:**
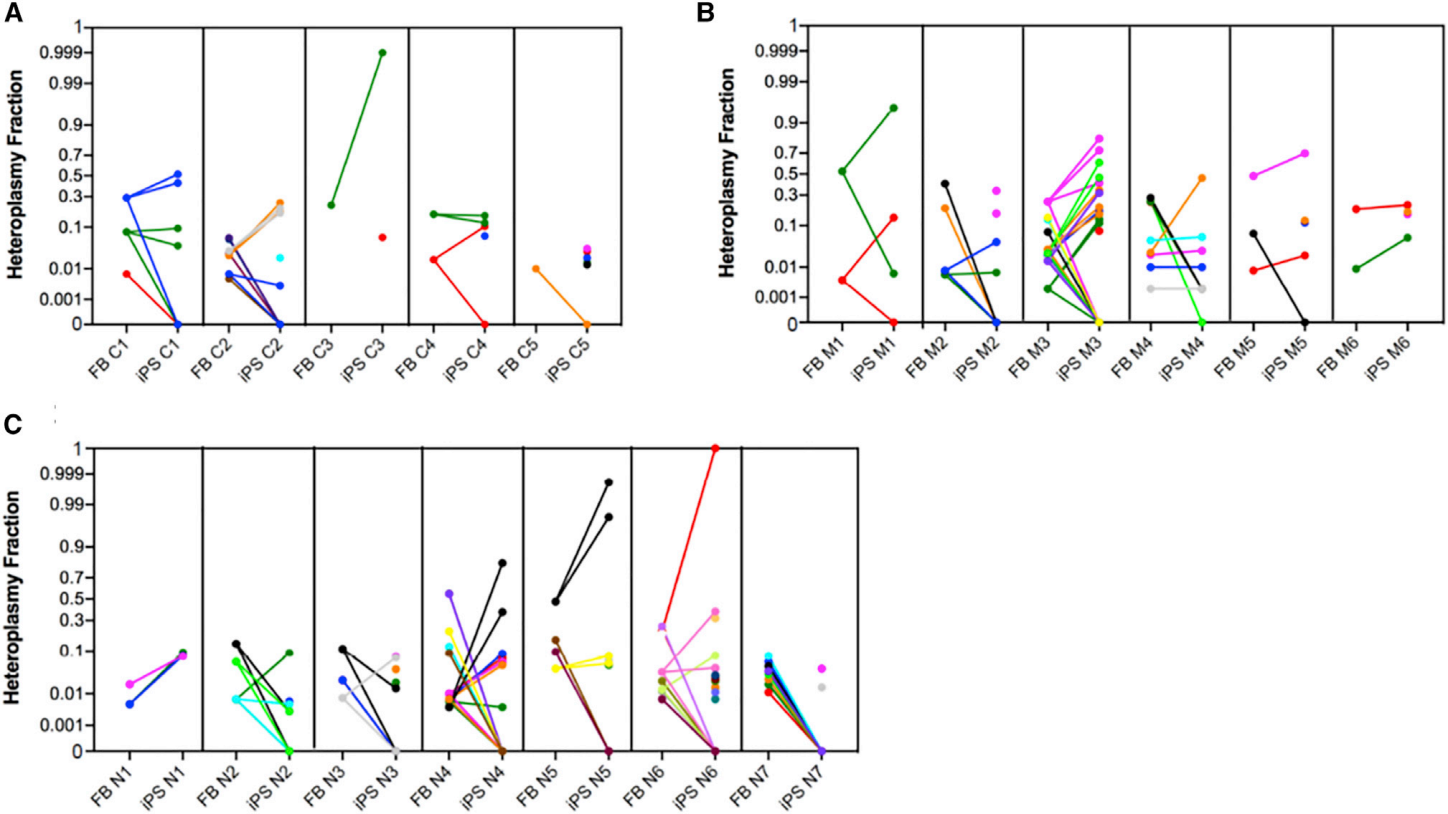
Fibroblasts to iPSCs heteroplasmy shift Variants’ HF fluctuations during the reprogramming step from fibroblasts to iPSCs in control (A), mitochondrial (B), and nuclear (C) groups. Dots appearing in iPSC mean unique variants. Non-transmitted variants are highlighted with a straight line going to zero in iPSC. One color represents one variant.

The analysis of private variants showed a high frequency of heteroplasmy in the three groups for a total of 120 heteroplasmic variants; the mitogenomes of the patients' groups did not harbor a higher number of variants in both fibroblasts (p = 0.082) and iPSCs (p = 0.234) (not shown). Moreover, in iPSCs, we observed a wide range of segregation models of the fibroblast variants (Figures S3 and S4): some iPSC clones completely lost the parental fibroblast variants, presenting (Figures S2G, S3D, S4C, and S4K) or not (Figures S2A and S4B) their own unique variants. Other iPSC clones harbored entirely (Figures S2F and S4A) or in part (Figures S2A, S2B, S2D, S3B, S3E, S3F, S4D, and S4E) only the variants present in their parental fibroblasts. However, the majority of iPSC clones contained both parental fibroblasts and unique variants in variable numbers and combinations (Figures 2A–2C). Although the highest variability was observed in the iPSCs carrying the *POLG* p.P648R mutation (N6), these did not display a significantly higher number of variants compared with the iPSCs derived from control, mitochondrial, or nuclear groups (Figure S5A).

We observed a general increase of heteroplasmy levels, which, in some cases, led variants heteroplasmic in fibroblasts to become homoplasmic in iPSCs as in C3 #7 (m.10377C>T from 23% to 99%–100%), M1 #32 (m.11150G>A from 53% to 95%), N5 #72-#75 (from 48% to 98%–100%), and N6 #2-#4-#8 (from 21% to 100%) (Figures 2A–2C). This incremental shift of heteroplasmy was also observed for the heteroplasmic pathogenic MELAS (mitochondrial encephalomyopathy, lactic acidosis, and stroke-like episodes) m.3243A>G mutation. In fact, iPSC clones derived from M3 fibroblasts, showing a 24% mutation load of the MELAS allele, displayed a broad distribution of the HF with one clone 100% wild-type (A), and others ranging from 41% to 81% (B, L, and N) of the mutant allele (Figure 2B, variant in magenta). However, exceptions to this general increase were observed in M1 #20 (m.11150G>T from 53% to 0.65%; Figure S3A), in M4 A1 (m.10158T>C from 24% to 0.2%, m.11157T>C from 25% to 0.2%, and m.13424T>C from 28% to 0.2%; Figure S3F), in N2 13M (m.10861T>C from 14% to 0.3% and m.16044T>C from 6% to 0.3%; Figure S4B) and in N3 #205 (m.13020T>C from 11% to 1%; Figure S4C). In contrast, the Leber hereditary optic neuropathy (LHON) m.11778G>A in M1 and m.3460G>A in M2, and the neuropathy ataxia retinitis pigmentosa (NARP) m.9185T>C in M4/M5/M6 remained homoplasmic in all iPSC clones as in the original fibroblasts.

### mtDNA heteroplasmy and variants segregations from iPSCs to NPCs

Sixteen iPSC clones, three in the control, five in the mitochondrial, and eight in the nuclear groups (Table 1), were differentiated in NPCs and checked for mtDNA private variants heteroplasmy. Globally, 86 heteroplasmic variants were observed in iPSCs/NPCs and the three groups did not differ in terms of variants number (p = 0.676; not shown). The large majority of the variants were shared between iPSCs and NPCs: only one NPC clone (#72 N5) presented just its own unique variants (Figure S4F), while other NPCs were more heterogeneous (#68 C2, 13M N2, and #4 N6) sharing two, two, and one variants with the iPSCs of origin, respectively (Figures S2D, S4B, and S4I).

**Figure 3:**
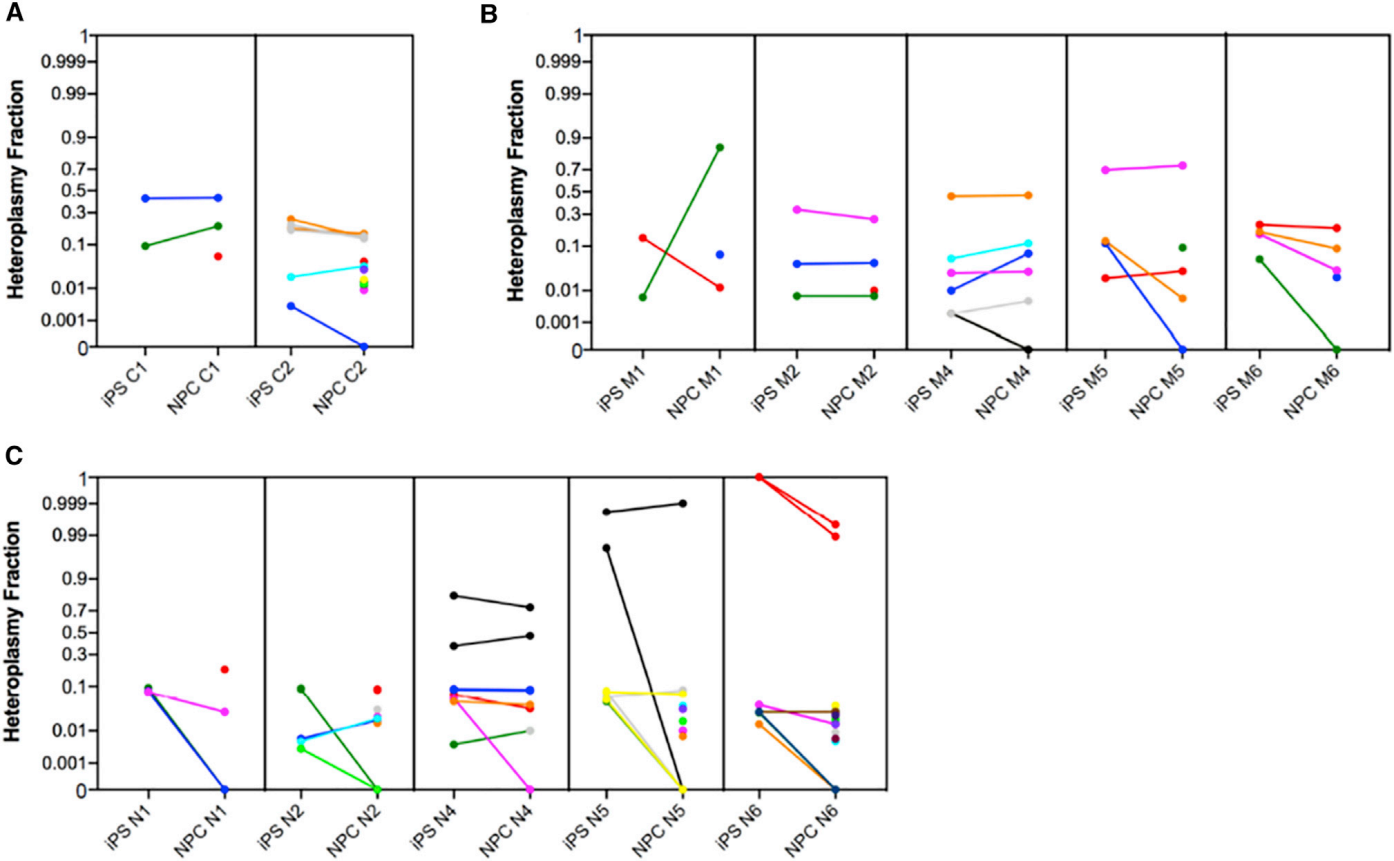
iPSCs to NPCs heteroplasmy shift Variants’ HF fluctuations during the differentiation step from iPSCs to NPCs in control (A), mitochondrial (B), and nuclear (C) groups. Dots appearing in NPC mean unique variants. Non-transmitted variants are highlighted with a straight line going to zero in NPC. One color represents one variant.

NPCs variants usually presented an HF similar to that of the iPSCs of origin, and even in the case of increment/reduction this was of limited magnitude and consistent with the direction of heteroplasmy change previously observed in the reprogramming step (Figures 3A–3C). A few exceptions were noted, as in M1 NPC #20 holding a variant with HF increasing from 0.65% to 85%, whereas in M4 NPC A1 the HF increase was less marked (from 6% to 11%); both variants were predicted to be benign. Moreover, C1 and N6 NPCs showed, respectively, a private benign variant in the *MT-TI* tRNA gene (6% HF) and a synonymous variant in the *MT-CYB* gene (0.6% HF), present in the parental fibroblasts at extremely low HF (0.7%), but apparently absent in their corresponding iPSCs (Figures S2B and S4J).

### mtDNA localization and prediction of variants transmitted in reprogramming and differentiating step

**Figure 4:**
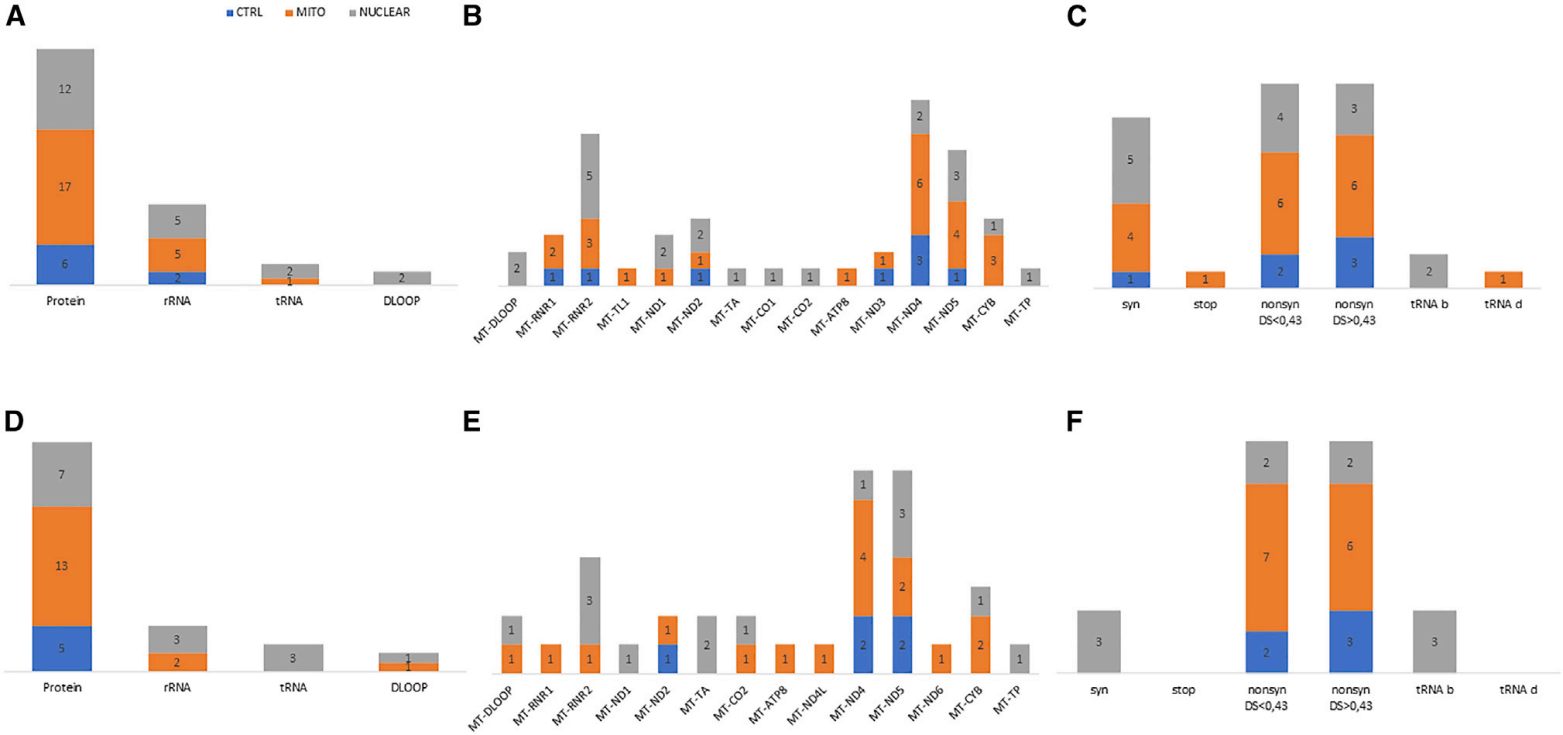
mtDNA localization and prediction of variants transmitted in reprogramming and differentiating steps in the three groups (A) Fifty-two heteroplasmic variants transmitted during the reprogramming step. (B) Mitochondrial genes localization of transmitted variants in iPSCs. (C) Predictions of transmitted variants in iPSCs. (D) Thirty-five heteroplasmic variants transmitted in the differentiating step. (E) Mitochondrial genes localization of transmitted variants in NPCs. (F) Predictions of transmitted variants in NPCs. syn, synonymous; nonsyn, nonsynonymous; b, benign; d, damaging.

Fifty-two out of 86 variants, with HF ranging from 0.2% to 100%, were transmitted during the reprogramming step, without any obvious bias among the three groups (p = 0.3) (Table S2). Specifically, eight variants were in the control, 23 in the mitochondrial, and 21 in the nuclear groups. We observed 35 variants in protein-coding genes, 12 rRNA genes, three in tRNA genes, but only two in the D-loop region (Figures 4A and S5B–S5D). *MT-ND4*, *MT-ND5*, and *MT-RNR2*, the three largest mtDNA genes, presented the greater number of variants, whereas *MT-ATP6*, *MT-CO3*, *MT-ND4L*, and *MT-ND6* did not show variants; *MT-TA*, *MT-TL1*, and *MT-TP* were the only tRNA genes carrying variants (Figure 4B). The variants’ transmission seemed to be independent from their pathogenicity in both control and patient groups. Most of the transmitted variants were predicted to be benign (24 of 38) and were in cells from affected patients (Figures 4C and S5E–S5G), but neither benign (p = 0.251) nor damaging (p = 0.428) nor non-coding (p = 0.809) variants were preferentially transmitted during the reprogramming step.

In the differentiating step, 35 variants ranging from HF 0.2%–100%, out of 52, were transmitted from iPSCs to NPCs: five in the control, 16 in the mitochondrial, and 14 in the nuclear groups (Table S3). The variants’ transmission was unaffected by the control/patient status of the NPCs (p = 0.74), and most of the variants were located in protein-coding genes. The remaining variants were in non-coding genes: two in the D-loop region, five in rRNA, and three in tRNA genes (Figures 4D and S5H–S5J). The majority of transmitted variants were in *MT-ND4* and *MT-ND5* genes, and all protein-coding genes, except *MT-ATP6*, *MT-CO1*, *MT-CO3*, and *MT-ND3*, presented with variants; *MT-TA* and *MT-TP* were the only tRNA genes carrying variants (Figure 4E). Even in iPSC to NPC transition, despite 17 out of 28 variants being predicted to be benign (Figure 4F and S5K–S5M), there was no difference in the transmission of benign (p = 0.559), likely damaging (p = 0.285), or non-coding (p = 0.621) variants in the three groups.

### Analysis of variants unique in iPSCs and NPCs

**Figure 5:**
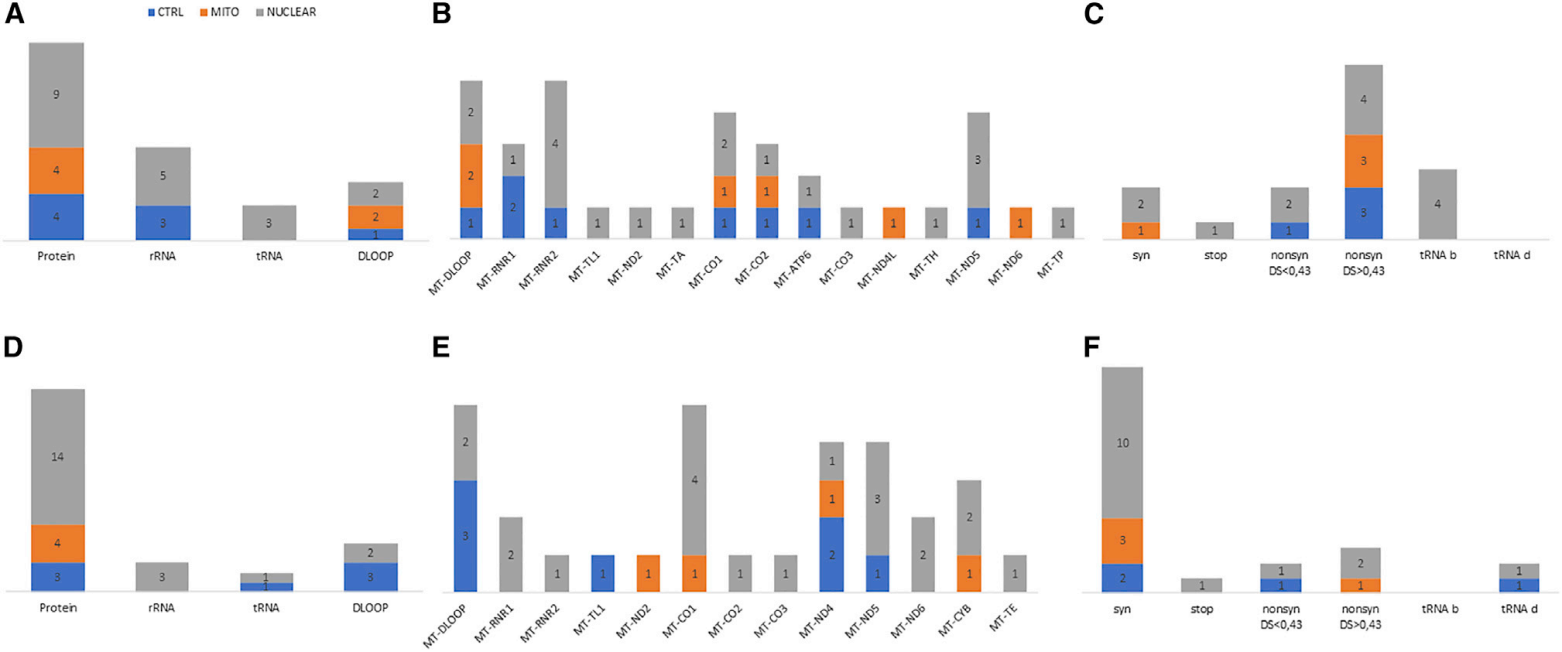
mtDNA localization and prediction of unique variants in iPSCs and NPCs in the three groups (A) Thirty-four heteroplasmic variants were unique in iPSCs clones. (B) Mitochondrial genes localization of transmitted variants in iPSCs. (C) Predictions of unique variants in iPSCs. (D) Thirty-one heteroplasmic variants were unique in the NPCs clones. (E) Mitochondrial genes localization of unique variants in NPCs. (F) Predictions of unique variants in NPCs. syn, synonymous; nonsyn, nonsynonymous; b, benign; d, damaging.

The variant m.7824C>T in *MT-CO2*, causing the amino acid change p.S80F predicted to be highly damaging (disease score 0.835 and Polyphen2 score 0.913), had high heteroplasmy levels (81% and 37%), in N4 iPSC clones #12 and #18 and derived NPC (72% and 47%), and was already present at only 0.4% in N4 parental fibroblasts (Figures S4D and S4E). Therefore, we focused on iPSCs unique variants, comprising both somatic variants and variants arising during reprogramming. We counted 34 unique variants: eight in the control, six in the mitochondrial, and 20 in the nuclear groups (Table S4). Although the average number of unique variants was not dependent on the presence of wild-type or mutated mitochondrial/nuclear DNA in the starting cell lines (p = 0.096), we noticed the highest variability in the N6 iPSC clone. Then, we tested whether these *POLG*-derived iPSCs accumulated more unique variants compared with all other iPSCs, and we found a statistically significant difference (Figure S5N). Five variants were in the D-loop region, eight in rRNA genes, four in tRNA, and 17 in protein-coding genes (Figure 5A). *MT-DLOOP* and *MT-RNR2* resulted in more variants, while *MT-ATP8*, *MT-CYB*, *MT-ND1*, *MT-ND3*, and *MT-ND4* did not have heteroplasmic variability; only *MT-TA*, *MT-TH*, *MT-TL1*, and *MT-TP* tRNA genes showed the presence of variants (Figure 5B). Annotation of the protein-coding variants revealed that one was a stop-gain, three were synonymous, while 13 were nonsynonymous, 10 of which were predicted to be deleterious (Figure 5C). However, we noticed that the nature of a variant (p = 0.213 for the non-coding variants) or its predicted pathogenicity (p = 0.888 and p = 0.920, respectively, for the benign and damaging variants) did not drive the occurrence as a unique variant in the three groups. Finally, interrogation of the GenBank database showed that 14 variants were never reported, whereas the most represented variants in the database were in the D-loop region and in rRNA genes (Table S4).

NPCs accumulated 31 unique variants (HF from 0.5% to 18.9%): seven in the control, four in the mitochondrial, and 20 in the nuclear groups (Table S5). We did not notice a significant difference in the average number between the three groups (p = 0.26), even though roughly half of the variants of the nuclear group were contributed by the N6 (*POLG*) patient. Five variants were located in the D-loop region, two in tRNA, three in rRNA genes, and 21 in protein-coding genes (Figure 5D). *MT-DLOOP* and *MT-CO1* accumulated more variants, while we did not observe variants in *MT-ATP6*, *MT-ATP8*, *MT-ND1*, *MT-ND3*, and *MT-ND4L* genes; *MT-TE* and *MT-TL1* were the only tRNA genes with variants (Figure 5E). Notably, the MELAS m.3243A>G mutation arose, at low heteroplasmy (1%), in C2 #68. Finally, also for NPC unique variants, notwithstanding that 17 out of 23 were predicted to be benign (Figure 5F), we failed to observe a significant difference in the occurrence of unique benign (p = 0.402), damaging (p = 0.767), or non-coding (p = 0.114) variants between controls and patients’ groups. Ten variants, located in the *MT-DLOOP* and rRNA, were never reported in GenBank (Table S5).

### Analysis of non-transmitted variants in iPSCs and NPCs

Thirty-two fibroblast variants (HF range 0.5%–55%) were non-transmitted in iPSC clones in a comparable way between groups (p = 0.357): five in the control, seven in the mitochondrial, and 20 in the nuclear groups (Table S6). One variant was in the D-loop region; nine in rRNA genes, with the 16s rRNA resulting in the gene non-transmitting the largest number of variants; four in the tRNA; and 18 in protein-coding genes, of which 13 were nonsynonymous predicted to likely be damaging (Figures S6A–S6C). In particular, two reported pathogenic variants, m.5540G>A with 5% HF (Bannwarth et al., 2013; Silvestri et al., 2000) and m.8993T>C with 19.6% HF (Kara et al., 2012; Weerasinghe et al., 2018), did not pass from C2 and M2 fibroblasts to iPSCs. Although 15 out of 22 non-transmitted variants were predicted as damaging, we noticed a statistical difference only for the non-transmitted non-coding variants among the mitochondrial and the nuclear groups (p = 0.02), whereas there was no difference for benign (p = 0.853) and damaging (p = 0.767) variants.

During the differentiation step, 16 variants with HF ranging from 0.2% to 11% were non-transmitted: one in the control, five in the mitochondrial, and 11 in the nuclear groups; the last two groups shared a variant in *MT-RNR2* (m.1693C>A) (Table S7). Importantly, also in this step, we did not observe a bias in the non-transmission of the variants among the three groups (p = 0.521). Eight variants were in non-coding regions in tRNA/rRNA genes (three in the D-loop region, one in a tRNA, and four in rRNA genes) (Figures S6D and S6E), whereas nine affected protein-coding genes, including four nonsynonymous variants predicted as deleterious (Figure S6F). Neither non-coding (p = 0.188), nor benign (p = 0.909), nor damaging (p = 0.086) variants were preferentially non-transmitted.

### Functional studies of mtDNA mutations in NPCs

**Figure 6:**
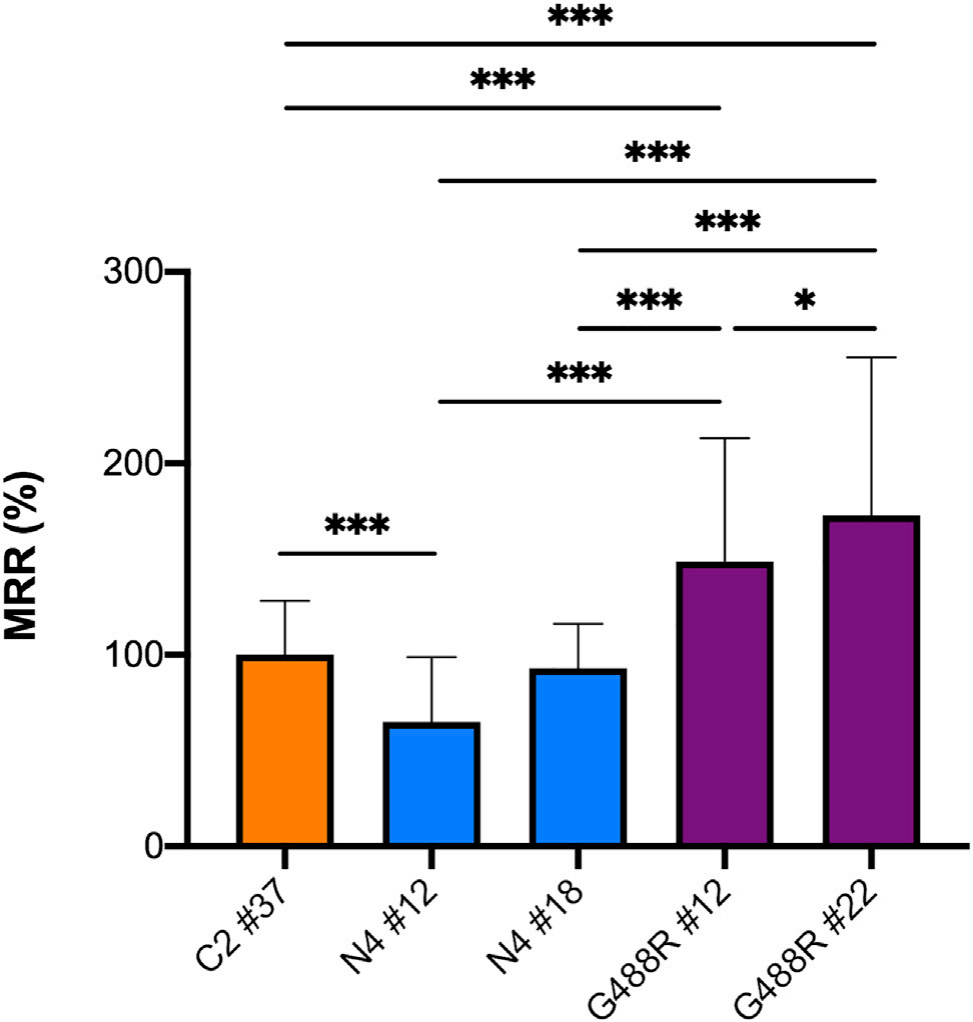
MRR of NPC Control (C2 #37), *OPA1* p.G488R mutation with (N4 #12 and N4 #18) or without (G488R #12 and G488R #22; Iannielli et al., 2018) p.S80F mutation in CO2 mtDNA sequence. Control = 100% MRR. ^∗^p < 0.05, ^∗∗∗^p < 0.001. All measurements were performed in 30 replicates for each sample. At least three different experiments in blind conditions to the examiner were carried out on different days.

To verify functional consequences of predicted pathogenic mtDNA mutations we used, as a paradigm, NPCs derived from iPSCs carrying variable levels of the p.S80F mutation (m.7824C>T) in the *MT-CO2* subunit gene of cytochrome c oxidase (COX), predicted to be highly pathogenic and never reported in Mitomap. We tested two independent NPCs (#12 and #18) obtained from patient N4, with 73% or 47% HF. Oxygen consumption rate showed a significant reduction of the maximal respiratory rate in NPC clone #12, and to a lesser extent in clone #18, as compared with control NPCs or NPCs derived from two clones (G488R#12 and G488R#22), generated from a different patient carrying an *OPA1* mutation identical (Iannielli et al., 2018) to the one present in patient N4, but with a wild-type mtDNA sequence (Figure 6).

## Discussion

Appearance of mtDNA variants during iPSCs reprogramming and their differentiation into specific lineages is a sensible area of investigation for the experimental and therapeutic use of iPSCs. We showed that, at each step from parental fibroblasts/PBMCs, to reprogrammed iPSCs, and then differentiated NPCs, HF of mtDNA variants, including those potentially pathogenic, fluctuate between emerging or disappearing or undergoing drift. The dynamics of these fluctuations may include bottleneck events but also genetic drifts in the absence of a clear purifying selection.

We performed deep mtDNA sequencing in several cell types, obtained from both control individuals and patients with mitochondrial diseases. The high coverage obtained, especially in fibroblasts, allowed us to confidently identify variants with a very low heteroplasmic load, missed by standard sequencing technologies, demonstrating the existence of universal mtDNA heteroplasmy, due to both inherited and somatic variants (Payne et al., 2013; Wei et al., 2019). We showed that low heteroplasmic variants (<1%) can be considered with high confidence true variants since those present with an extremely low HF (from 0.2% to 1%) in fibroblasts were identified in derived iPSCs and/or NPCs and unique variants were shared among different iPSCs or NPCs, indicating their probable origin from the parental fibroblasts. Fibroblasts are pooled cells with high mtDNA sequence heterogeneity and certain variants, even present at high HF in a single cell, ultimately appear diluted or even absent in the cell culture. Thus, we might consider as an initial bottleneck event the clonal nature of iPSCs reprogramming from a single fibroblast, highlighting its parental heteroplasmic profile. Ultimately, the mtDNA variants observed in the iPSCs may reflect either true *de novo* events or pre-existing heteroplasmic variants buried under the detection threshold in the parental fibroblasts, but expanding in the clonally reprogrammed cells. Congruently, it was shown that MELAS iPSC clones shifted either close to zero or to high mutant load during reprogramming, whereas fibroblast cultures showed a wide spectrum of heteroplasmy states (Hämäläinen et al., 2013). This scenario was already described and discussions revolved around the issue of whether the iPSCs variants were pre-existing and detectable in the original fibroblasts cell culture or not (Kang et al., 2016; Perales-Clemente et al., 2016).

Previous studies underlined the correlation of mtDNA variants in iPSCs with the age of patients originating the primary cell culture (Kang et al., 2016). We confirm this age-related increase of mtDNA variants in fibroblasts, as shown by simply comparing the young (aged 12–49 years) and old (aged 50–80 years) subjects. However, we failed to detect the same correlation either in iPSCs or in NPCs, even if in Kang’s study there was also a consistent trend in reducing the amount of variants across reprogramming (see Figure 3E in Kang et al., 2016). The reprogramming procedure implies a sort of rejuvenation process (Goya et al., 2018) in which iPSCs derived from old individuals could reset their genetic heritage through multiple passages and, in doing so, erase the mtDNA alterations accumulated during aging in the parental fibroblasts. Our data are consistent with the number of passages of the iPSCs (15–20), whereas those analyzed by Kang had a significantly lower (2–4) number of passages (Kang et al., 2016), when iPSCs display the greatest heterogeneity and variability (Volpato and Webber, 2020). The hypothesis of germline purifying selection based on the functional impact of mtDNA mutations has been previously proposed in murine models, acting most efficiently against variants affecting protein-coding genes (Stewart et al., 2008). Our results to some extent provide the same indication of preferential selection against mtDNA mutation in protein-encoding genes. It has also been reported that the MELAS mutation, affecting a tRNA, progressively reduced its heteroplasmic load with increasing iPSCs passages in prolonged cell culture (Folmes et al., 2013). Thus, even if a purifying selection against specific damaging variants cannot be excluded, we here demonstrated that pathogenic variants are present in iPSCs and maintained in derived NPCs. This may suggest a different surveillance mechanism to explain the vanishing of age-related differences, which might go beyond mtDNA. It remains that iPSCs rely mainly on glycolysis, explaining the tolerance of damaging variants affecting the OXPHOS system. Further investigations are required to fully understand the rejuvenation or purifying selection.

The iPSCs carrying haplogroup J1c, but neither fibroblasts nor NPCs, had a significantly reduced variants number compared with the most frequent European haplogroup H. This points to something happening at the reprogramming step or in the culturing passages of iPSCs. The nuclear background or the existence of an mtDNA pathogenic mutation were irrelevant as the three haplogroup J1c cases were respectively a control and a LHON patient, in the young group, and a Twinkle patient, in the old group. Haplogroup J is solidly associated with LHON, as a background enhancing penetrance (Carelli et al., 2006; Hudson et al., 2007; Torroni et al., 1997). Haplogroup J has also been associated with longevity in an Italian population (De Benedictis et al., 1999), but not in others (Rose et al., 2001), and the JT superhaplogroup has been proposed to be protective in Parkinson disease (Hudson et al., 2013). Testing haplogroup J in cybrids highlighted a slightly inefficient OXPHOS compared with other haplogroups, in particular haplogroup H, which may enhance the pathogenicity of LHON mutations, but in the long run accumulate less somatic mutations favoring longevity and protecting from Parkinson (Gómez-Durán et al., 2012; Strobbe et al., 2018). This seems recapitulated in the acute model of iPSCs generation, as emphasized by our results of reduced propensity to accumulate mtDNA variants in haplogroup J.

Overall, most of the reported variants increased the level of heteroplasmy from fibroblast to iPSCs. Once fixed in iPSC clones, these variants were, in most cases, present and maintained at a similar heteroplasmic level in NPCs, suggesting that, during this transition, no major modulations of mtDNA amount occur, although additional mtDNA variants may arise. This latter event was, however, mostly, but not exclusively, occurring in *POLG* mutant cells, characterized by defective mtDNA replication. The *POLG* mutation (Table 1), a homozygous p.P648R change hitting the spacer domain of the protein, potentially alters enzyme activity, processivity, DNA-binding affinity, or affects interactions with the mitochondrial single-stranded DNA-binding protein a partner protein, part of the mitochondrial “replisome” complex (Luo and Kaguni, 2005). It is therefore conceivable to observe an increase in mutation frequency in the *POLG* mutant cell line endowed with a crippled enzyme. This does not exclude the already mentioned pre-existence of buried variants emerging as apparently *de novo* variants. In fact, in fibroblasts, we failed to observe a difference in total variants load, which became apparent in terms of *de novo* variants only after reprogramming. A similar situation occurs in the single Twinkle iPSC clone, another gene involved in mtDNA maintenance. Remarkably, in *POLG*-, *Twinkle*-, and *OPA1*-derived iPSC clones, we failed to observe mtDNA deletions, usually accumulating in post-mitotic tissues of patients (Carelli and Chan, 2014).

We did not observe the presence of recurrent mtDNA variants, nor obvious mutational hot spots or genes more prone than others to genetic variation, except for the m.1693C>A variant in the *MT-RNR2*, detected in three unrelated iPSCs. This novel variant was absent in the Mitomap repository, and was not recognized by previous studies of the iPSCs mitogenome (Kang et al., 2016; Perales-Clemente et al., 2016). Most reported variants affected protein-coding genes and roughly half of them were potentially damaging. As proof of principle, we studied the p.S80F mutation in *MT-CO2*, present at extremely low heteroplasmic level in *OPA1* fibroblasts, and expanded during reprogramming in two independent iPSCs clones and derived NPCs. This mtDNA variant caused a severe impairment of the maximal respiratory capacity, which could cause, if unaware, a misinterpretation of the functional effect of the *OPA1* mutation.

Overall, we found about equal amounts of variants in structural genes encoding OXPHOS subunits and in genes dedicated to the mtDNA translation (tRNAs/rRNAs). This contrasts with the total size of the protein-encoding genes (11.3 kb), against the size of the translation-dedicated mtDNA genes (3.9 kb), which may suggest some degree of purifying selection mostly acting on protein-coding genes (Stewart et al., 2008). It remains that clearly pathogenic variants are tolerated during reprogramming and subsequent differentiation. Interestingly, there seems to be preferential occurrence of mtDNA variants hitting a few specific tRNAs and, more consistently, the *MT-RNR2*. Some of the tRNAs (*MT-TI*, *MT-TW*, *MT-TH*, *MT-TA*, *MT-TE*, *MT-TP*) are shared by Kang et al. (2016). However, it is unclear if there is some selectivity in mtDNA mutagenesis or if some degree of purifying selection is acting through reprogramming/differentiation, or if a combination of both mechanisms may occur.

Despite previous and the present studies reported the importance of analyzing both the mitochondrial and nuclear genome in iPSCs, mtDNA sequencing is rarely incorporated in the quality control check of iPSCs (Attwood and Edel, 2019; Doi et al., 2020; Doss and Sachinidis, 2019). Remarkably, the first iPSCs therapy for Parkinson disease started in 2018 (http://www.cira.kyoto-u.ac.jp/e/pressrelease/news/180730-170000.html) but the clinical grade iPSCs characterization did not seem to include mtDNA analysis. Likewise, various studies documenting mitochondrial dysfunctions in neurodegenerative diseases, such as multiple system atrophy (Monzio Compagnoni et al., 2018), amyotrophic lateral sclerosis(Dafinca et al., 2016), Parkinson disease (Little et al., 2018), and Alzheimer disease (Birnbaum et al., 2018), did not report mtDNA analysis of iPSCs. We here showed that mtDNA variants occur not only in iPSCs but also in NPCs, adding concerns when working with this *in vitro* system.

Our study remarked that generation of iPSCs is consistently affected by events of expansion/reduction or *de novo* generation of heteroplasmic variants in mtDNA, potentially deleterious, ultimately affecting the global healthiness of the iPSCs. This has profound implications for further differentiated cells or organoids (Lancaster and Knoblich, 2014) used to model diseases, but particularly for *in vivo* therapeutic use of iPSCs in humans, as for Parkinson patients (Fan et al., 2020; Stoddard-Bennett and Pera, 2020). Furthermore, it was recently shown that nonsynonymous mtDNA mutations in iPSCs may lead to neoantigens eliciting an immune response, indicating that autologous iPSCs may not be immunologically inert (Deuse et al., 2019).

In conclusion, the mtDNA sequence profile of iPSCs is an unavoidable step to ensure that these cells are suitable for modeling diseases and testing experimental treatments. A systematic study of the dynamic changes in mtDNA variants occurrence and segregation also provides a great opportunity to better understand all issues related to universal heteroplasmy. Ultimately, checking genetic stability of iPSCs and evaluating nuclear and mitochondrial genomes is of paramount importance for quality-grade iPSC-based clinical trials.

## Experimental procedures

### Genetic characterization of patients’ derived fibroblasts

Fibroblasts derived from 13 patients affected by mitochondrial disorders and five healthy age-matched subjects were included in this study and obtained from the cell lines and DNA bank of pediatric movement disorders and mitochondrial diseases of the Telethon Network of Genetic Biobanks (http://biobanknetwork.telethon.it). Patients were classified into a mitochondrial group (M1 to M6) with either homoplasmic or heteroplasmic mtDNA mutations, or a nuclear group (N1 to N7) with mutations in nuclear genes coding for mitochondrial proteins (Table1).

### Generation and Characterization of iPSCs

We used either already generated or newly produced iPSCs. On average, passages of iPSCs in culture were in the range between 15 and 20. Table 1 summarizes all the relevant information.

iPSCs for NARP mutation were generated from fibroblasts derived from a three-generation family carrying the homoplasmic m.9185T>C mutation. Subjects M4, M5, and M6 in Table 1 correspond to patient A1 (patient III-6), patient A2 (daughter of A1, patient 1, IV-5), and patient A3 (third-degree cousin of A2, patient 2, V-5) respectively (Auré et al., 2013). iPSCs for patient M4 were generated by episomal plasmid, and for patients M5 and M6 by transduction with retrovirus. Characterization and mtDNA Sanger sequence analysis were performed as reported (Lorenz et al., 2017). iPSCs for MELAS mutation were generated from fibroblasts as described (Hämäläinen et al., 2013). The mutation load was determined in fibroblasts and derived iPSCs by minisequencing (Suomalainen et al., 1993). Available clones reported in Table 1 have the following heteroplasmic levels: #A = 4.2%, #B = 2.5%, #L = 79.7%, and #N7 = 2.5%. iPSCs for dominant optic atrophy (DOA) and pantothenate kinase-associated neurodegeneration (PKAN) were generated from primary fibroblasts as described (Iannielli et al., 2018; Orellana et al., 2016). New iPSCs for DOA and LHON were generated and characterized as described (Iannielli et al., 2018; Peron et al., 2020).

### CGH array

Integrity of nuclear DNA was verified by array comparative genomic hybridization (CGH) as described (Peron et al., 2020).

### mtDNA sequencing

Sequence analysis of the entire mtDNA molecule was performed by the NGS approach (Caporali et al., 2018). The primers used to amplify the mtDNA molecule in two segments are strategically located outside the regions involved in the generation of breakpoints underlying mtDNA deletions. The NGS libraries were constructed by Nextera XT (Illumina) and paired end sequenced on MiSeq System (Illumina), using MiSeq Reagent Kit v3 (600 cycles).

Fastq files were analyzed with MToolBox v1.1 and v.1.2 (https://github.com/mitoNGS/MToolBox) (Calabrese et al., 2014). Only mono-allelic SNVs (single nucleotide variant) with a read depth ≥100 and a base quality score ≥30 were retained. All private heteroplasmic SNVs were visually inspected with the tool IGV (Integrative Genome Viewer: https://software.broadinstitute.org/software/igv/) in order to check the variants' strand bias, and strongly unbalanced variants were discarded. Reads depths (total, forward, and reverse) for unique variants with HF ≤ 2% are reported in Table S8.

Nonsynonymous variants with a disease score >0.43 were predicted to be deleterious, while for tRNA variants we considered the MitoTIP prediction.

### Statistical analysis

Statistical analyses were performed with GraphPad Prism 6.0. Differences between mean values of variants with respect to age of cell line donors and to reprogramming method were assessed by the Mann-Whitney unpaired t test. Univariate linear regression analysis was performed to assess the effect of age on the number of variants in the cell lines. Differences and multiple comparisons in the three groups were estimated with the Krustal-Wallis, one-way ANOVA test. A p value ≤0.05 was considered significant.

### Determination of respiratory activity

Oxygen consumption rate (OCR) was measured in DOA and control NPCs with an XF96 Extracellular Flux Analyzer (Seahorse Bioscience, Billerica, MA). NPCs were seeded at a density of 20,000 cells/well and measurement was performed as described (Invernizzi et al., 2012). Maximal respiration rate (MRR) was calculated as percentage of control.

### Data and code availability

The SRA (Sequence Read Archive) accession number for the data reported in this paper is PRJNA706687.

## Authors contributions

Conceptualization, V.C. and V.T.; methodology, F.P., C.P., A.I., I.D.M., A.M., L.C., S.L., A.P., A.S., and V.B.; investigation, F.P., C.P., A.I., L.C., C.F., A.S., F.L.S., and A.R.; resources, C.P., A.I., S.L., A.P., A.S., and V.B.; data curation, F.P., C.F., and L.C.; writing – original draft, F.P., C.P., V.C., and V.T.; writing – review & editing, all authors; funding acquisition, V.C., V.B., V.T.; supervision, V.C., V.B., and V.T.

## Declaration of interests

The authors declare no competing interests.

## Supplemental information

Document S1. Figures S1–S6 and Tables S1–S8

Document S2. Article plus supplemental information
