# Supplementary material for: The relevance of mitochondrial DNA variants fluctuation during reprogramming and neuronal differentiation of human iPSCs
Source: Stem Cell Reports. 2021 Jul 29;16(8):1953–67. doi: 10.1016/j.stemcr.2021.06.016 (PMC8365099; doi:10.1016/j.stemcr.2021.06.016)
Supplement: Document S1. Figures S1–S6 and Tables S1–S8 [file mmc1.pdf]

Stem Cell Reports, Volume *16*

# Supplemental Information

## The relevance of mitochondrial DNA variants fluctuation during reprogramming and neuronal differentiation of human iPSCs

**Flavia Palombo, Camille Peron, Leonardo Caporali, Angelo Iannielli, Alessandra Maresca, Ivano Di Meo, Claudio Fiorini, Alice Segnali, Francesca L. Sciacca, Ambra Rizzo, Sonia Levi, Anu Suomalainen, Alessandro Prigione, Vania Broccoli, Valerio Carelli, and Valeria Tiranti**

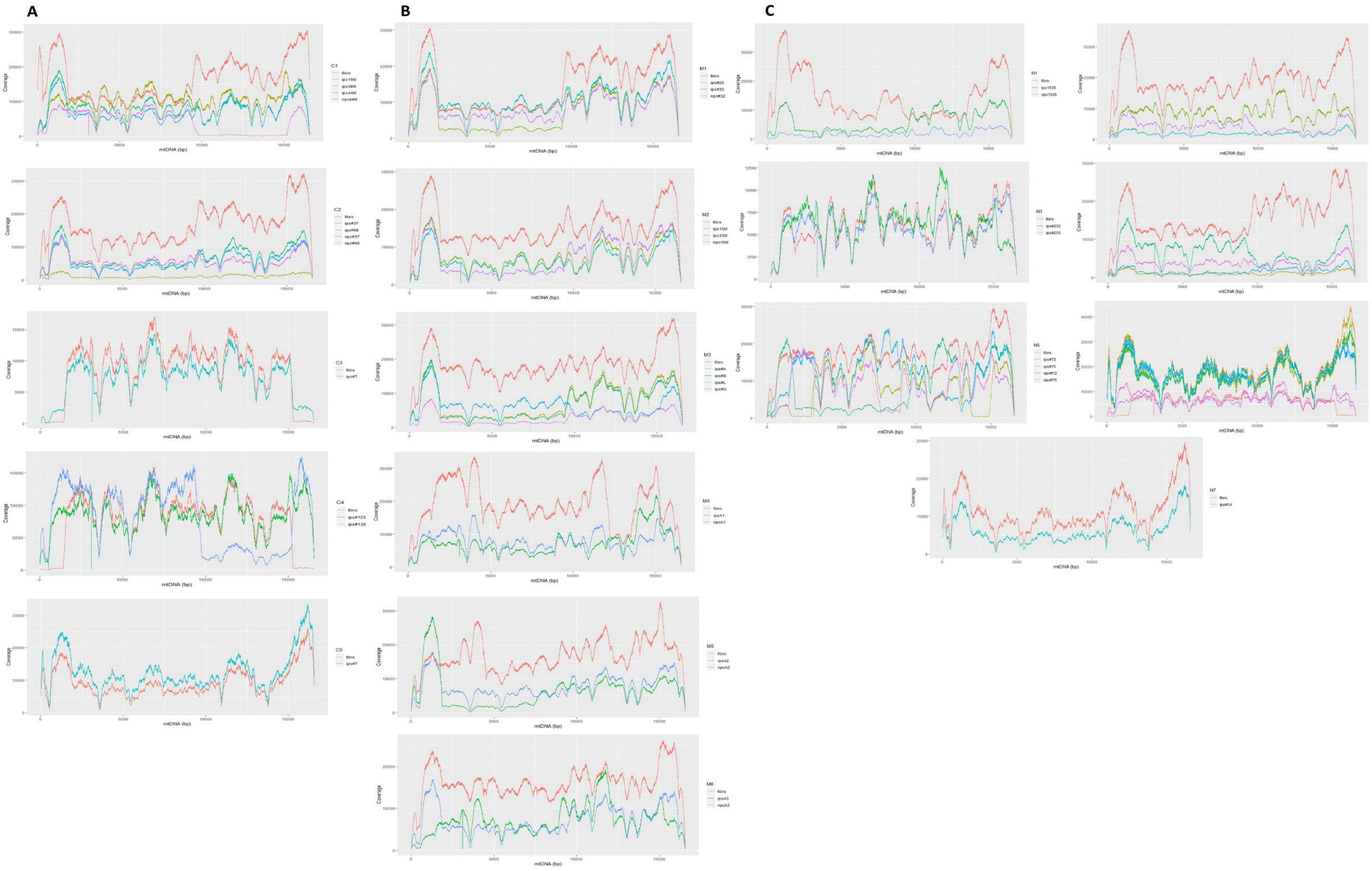


**Figure S1. mtDNA NGS coverage in CONTROL (A), MITO (B) and NUCLEAR (C) group.**

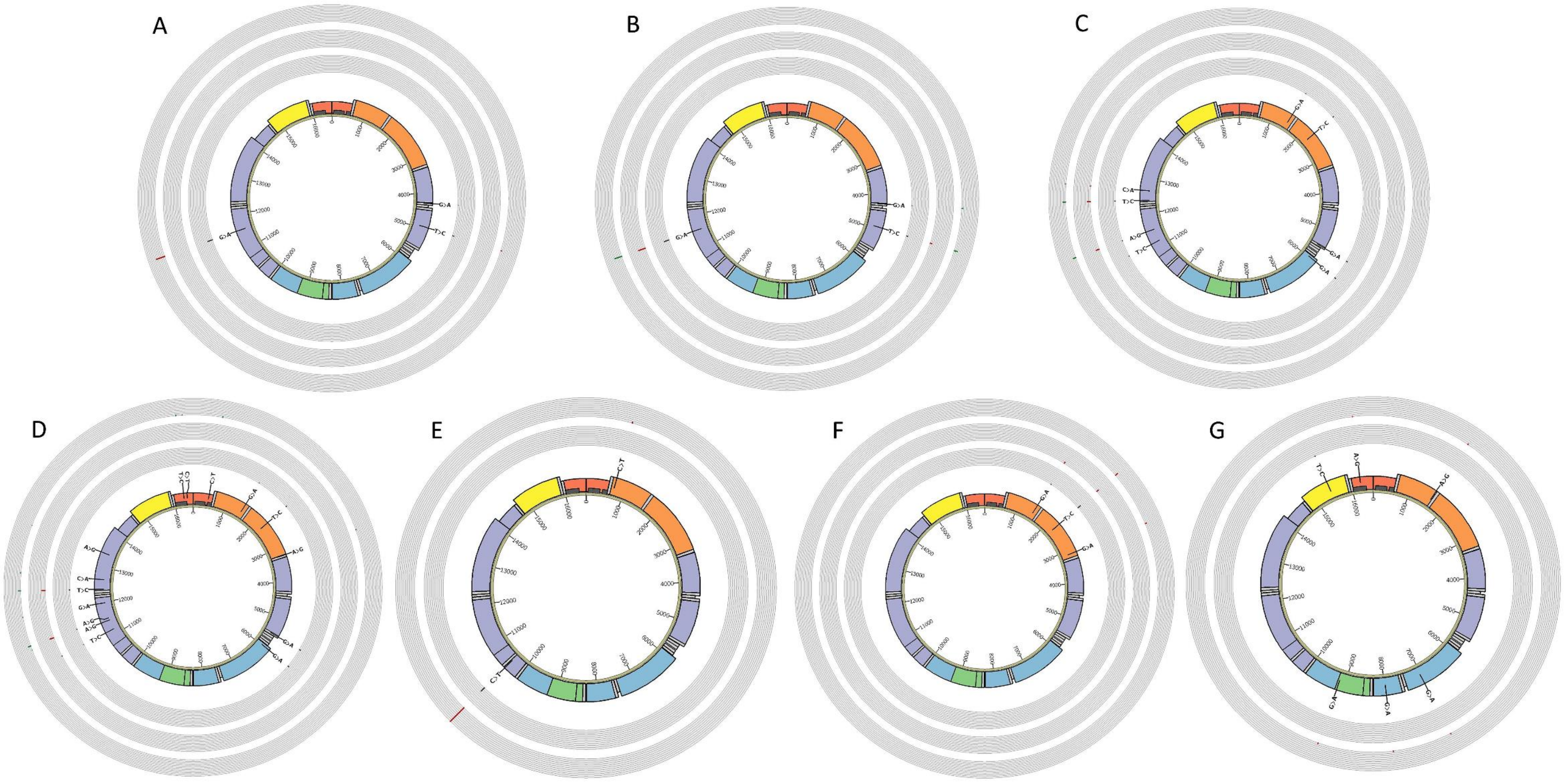


**Figure S2. Circos plot of the structure of the human mitochondrial genome showing CONTROL heteroplasmic variants.** Colored boxes represent: MT-DLOOP (red), transfer RNAs (light gray), ribosomal RNAs (orange), Complex I genes (purple), Complex III gene (yellow), Complex IV genes (blue) and Complex V genes (green). Shorter boxes are antisense transcripts, while others are sense transcripts. Dark gray bars represent hypervariable regions. The innermost circle with black lines showed fibroblasts heteroplasmic variants, the outer circles with red lines showed iPSCs heteroplasmic variants and the outermost circle with green lines showed NPCs heteroplasmic variants; the length of the lines are proportionate to variants heteroplasmic loads. A. C1 fibroblasts, 19M and 38M iPSC clones. B. C1 fibroblasts, 44M iPSC and NPC clones. C. C2 fibroblasts, #37 iPSC and NPC clones. D. C2 fibroblasts, #68 iPSC and NPC clones. E. C3 fibroblasts and #7 iPSC clone. F. C4 fibroblasts, #123 and #130 iPSC clones. G. C5 fibroblasts and #105 iPSC clone.

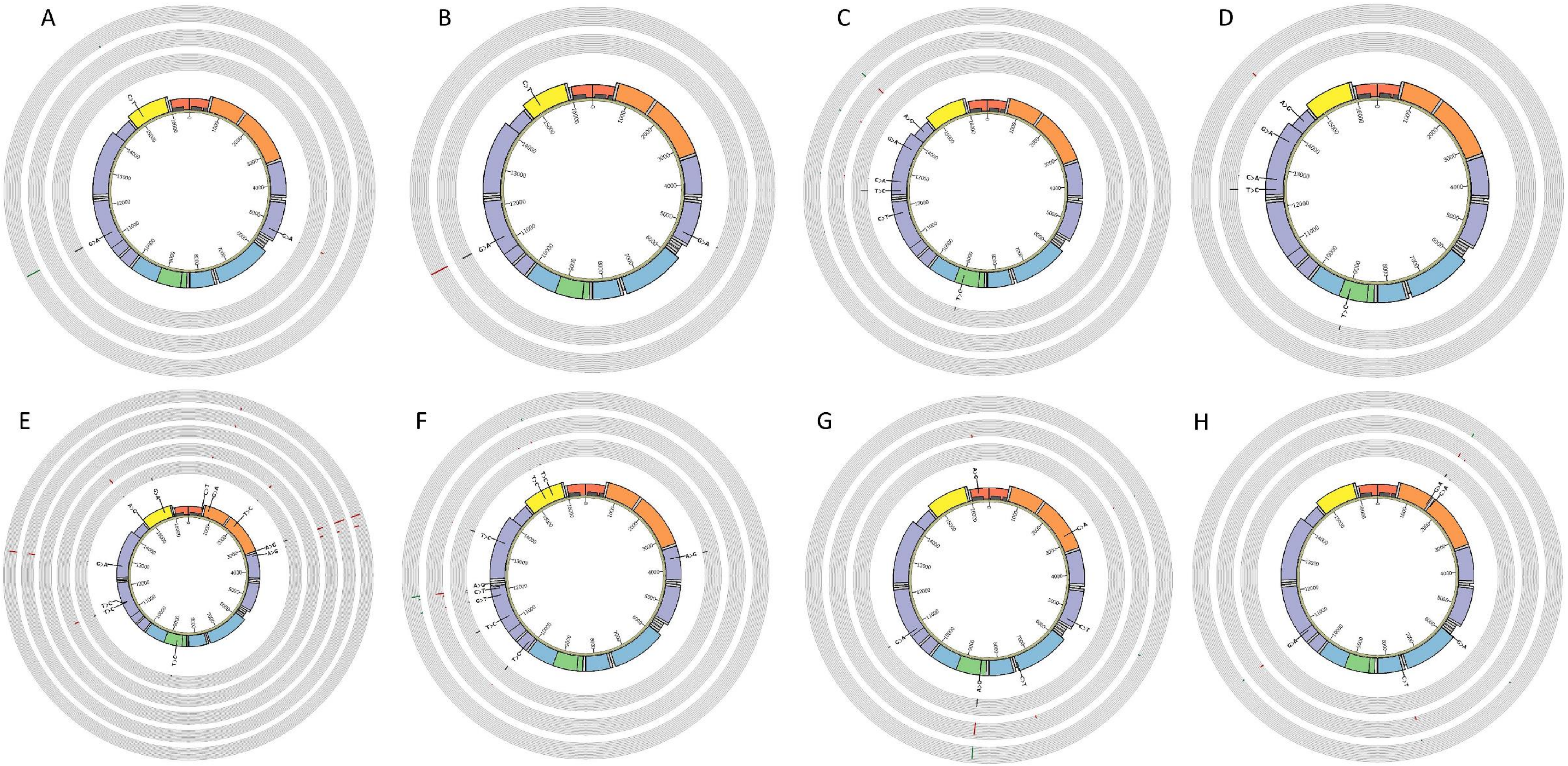


**Figure S3. Circos plot of the structure of the human mitochondrial genome showing MITO heteroplasmic variants.** A. M1 fibroblasts, #20 iPSC and NPC clones. B. M1 fibroblasts and #32 iPSC clone. C. M2 fibroblasts, 15M iPSC and NPC clones. D. M2 fibroblasts and 33M iPSC clone. E. M3 fibroblasts, #A, #B, #L and #N iPSC clone. F. M4 fibroblasts, A1 iPSCs and NPC clones. G. M5 fibroblasts, A2 iPSC and NPC clones. H. M6 fibroblasts, A3 iPSC and NPC clones.

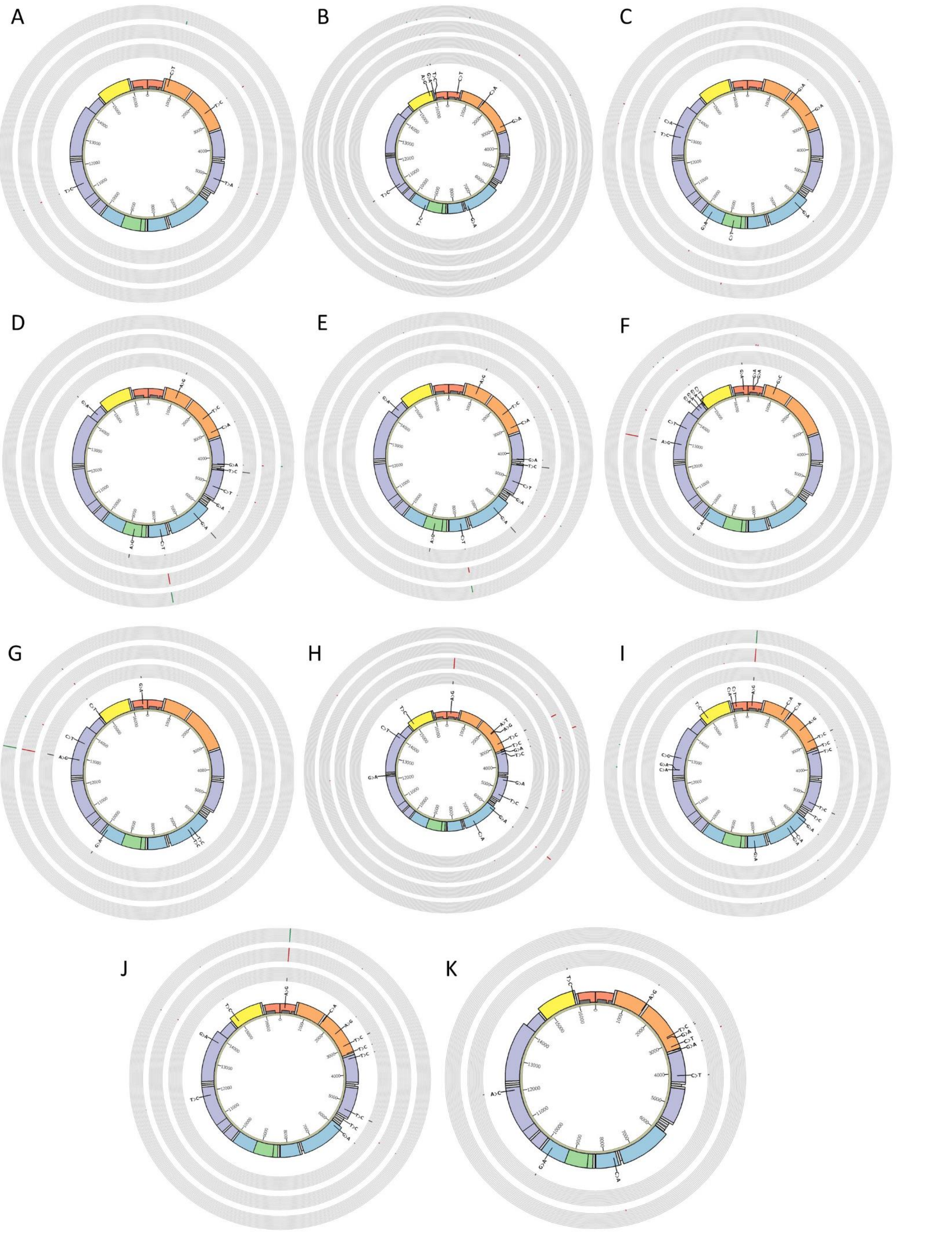


**Figure S4. Circos plot of the structure of the human mitochondrial genome showing NUCLEAR heteroplasmic variants**. A. N1 fibroblasts, 1535 iPSC and NPC clones. B. N2 fibroblasts, 13M iPSC and NPC clones. C. N3 fibroblasts, #202 and #205 iPSC clones. D. N4 fibroblasts, #12 iPSC and NPC clones. E. N4 fibroblasts, #18 iPSC and NPC clones. F. N5 fibroblasts, #72 iPSC and NPC clones. G. N5 fibroblasts, #75 iPSC and NPC clones. H. N6 fibroblasts, #2, #3 and #6 iPSC clones. I. N6 fibroblasts, #4 iPSC and NPC clones. J. N6 fibroblasts, #8 iPSC and NPC clones. K. N7 fibroblasts and #34 iPSC clone

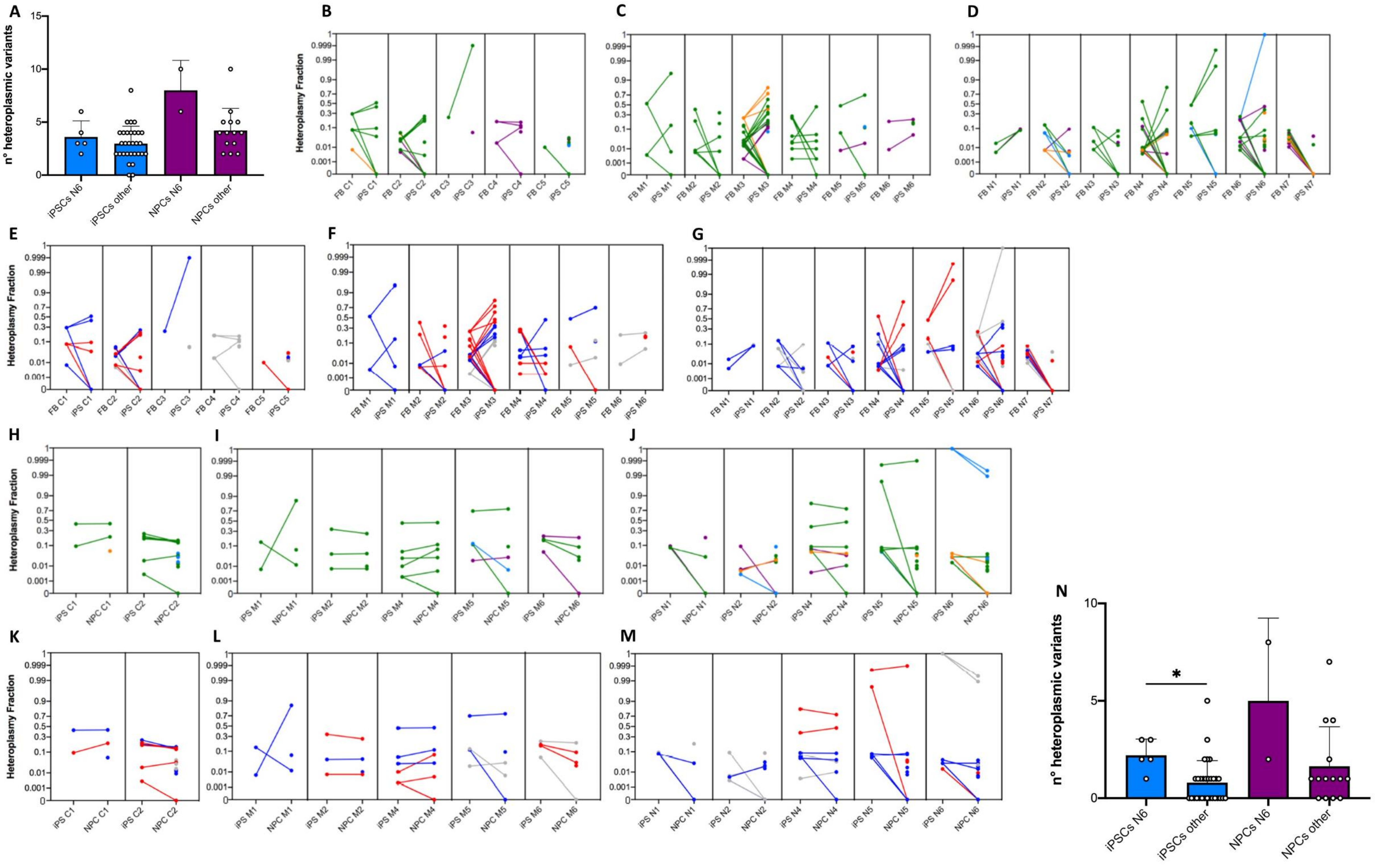

A
n° heteroplasmic variants
15
10
5
0
iPSCs N6
iPSCs other
NPCs N6
NPCs other
B
Heteroplasmy Fraction
1
0.999
0.99
0.9
0.7
0.5
0.3
0.1
0.01
0.001
0
FB C1
iPS C1
FB C2
iPS C2
FB C3
iPS C3
FB C4
iPS C4
FB C5
iPS C5
C
FB M1
iPS M1
FB M2
iPS M2
FB M3
iPS M3
FB M4
iPS M4
FB M5
iPS M5
FB M6
iPS M6
D
FB N1
iPS N1
FB N2
iPS N2
FB N3
iPS N3
FB N4
iPS N4
FB N5
iPS N5
FB N6
iPS N6
FB N7
iPS N7
E
F
G
H
iPS C1
NPC C1
iPS C2
NPC C2
I
iPS M1
NPC M1
iPS M2
NPC M2
iPS M4
NPC M4
iPS M5
NPC M5
iPS M6
NPC M6
J
iPS N1
NPC N1
iPS N2
NPC N2
iPS N4
NPC N4
iPS N5
NPC N5
iPS N6
NPC N6
K
L
M
N
n° heteroplasmic variants
10
5
0
*
iPSCs N6
iPSCs other
NPCs N6
NPCs other

**Figure S5. Analysis and characterization of heteroplasmic variants.** A. iPSC and NPC clones obtained from the N6 patient carrying the *POLG* p.P648R mutation did not display a significantly higher number of heteroplasmic variants compared to the iPSCs (p=0.417) and the NPCs (p=0.06) derived from controls and from the MITO or NUCLEAR groups. B-D. Variants HF fluctuations during the reprogramming step from fibroblasts to iPSCs in CONTROL (B), MITO (C) and NUCLEAR (D) groups. Dots appearing in iPSC means unique variants. Non transmitted variants are highlighted with a straight line going to zero in iPSC. blue= MT-DLOOP violet=rRNA orange=tRNA green=coding genes. E-G. Variants HF fluctuations during the reprogramming step from fibroblasts to iPSCs in CONTROL (E), MITO (F) and NUCLEAR (G) groups. Dots appearing in iPSC means unique variants. Non transmitted variants are highlighted with a straight line going to zero in iPSC. blue=benign red=damaging gray=no prediction. H-J. Variants HF fluctuations during differentiation step from iPSCs to NPCs in CONTROL (H) MITO (I) and NUCLEAR (J) groups. Dots appearing in NPC means unique variants. Non transmitted variants are highlighted with a straight line going to zero in NPC. blue= MT-DLOOP violet=rRNA orange=tRNA green=coding genes. K-M. Variants HF fluctuations during differentiation step from iPSCs to NPCs in CONTROL (K), MITO (L) and NUCLEAR (M) groups. Dots appearing in NPC means unique variants. Non transmitted variants are highlighted with a straight line going to zero in NPC. blue=benign; red=damaging; gray=no prediction. N. iPSC clones obtained from the N6 patient carrying the *POLG* p.P648R mutation accumulated a significantly higher number of unique variants compared to all other iPSCs (p=0.003), differently from the NPC clones (p=0.09).

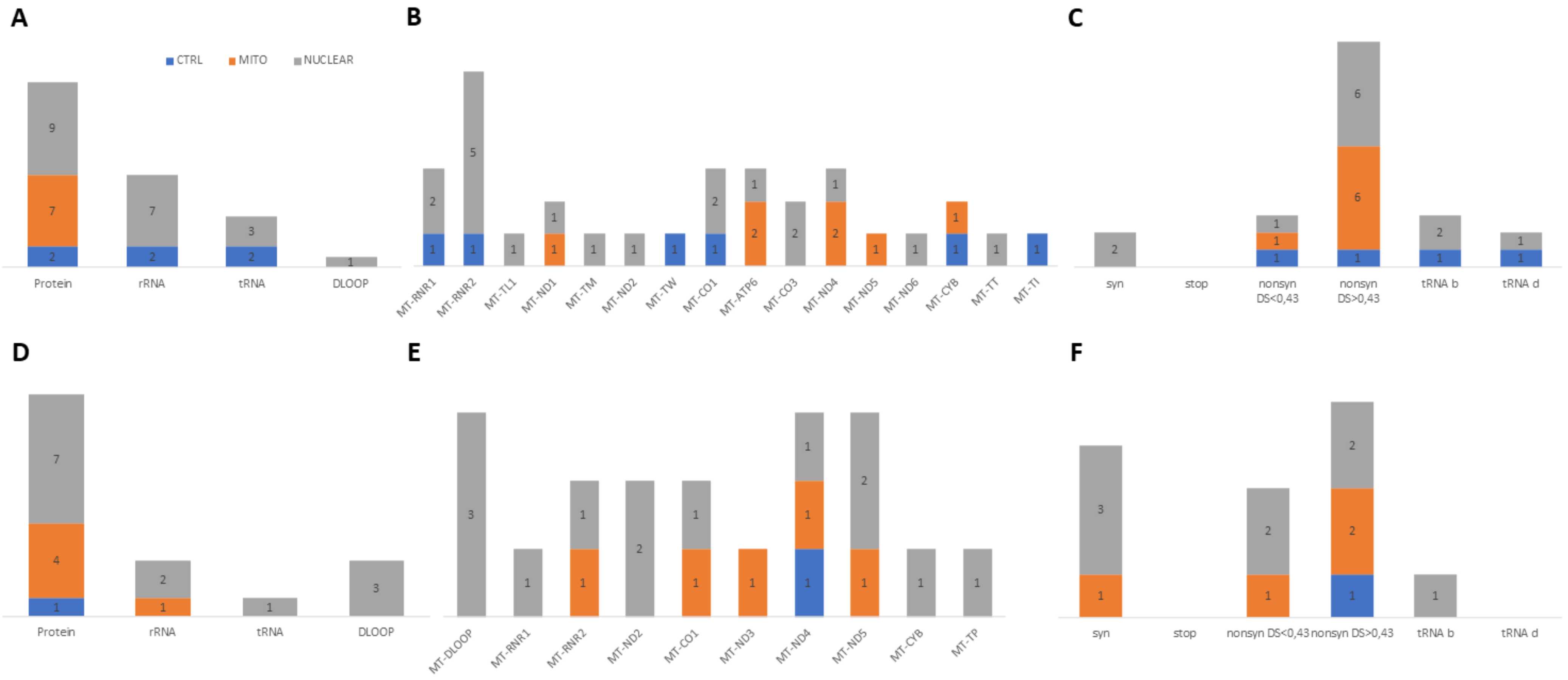


**Figure S6. mtDNA localization and prediction of variants non-transmitted in reprogramming and differentiating steps in the three groups.** A. Thirty-two heteroplasmic variants were non-transmitted to the iPSCs clones during the reprogramming step. B. Mitochondrial genes localization of non-transmitted variants in iPSCs. C. Predictions of non-transmitted variants in iPSCs. D. Sixteen heteroplasmic variants were non-transmitted to the NPCs clones in the differentiating step. E. Mitochondrial genes localization of non-transmitted variants in NPCs. F. Predictions of non-transmitted variants in NPCs. syn=synonymous; nonsyn=nonsynonymous; b=benign; d=damaging

| Fibroblasts | Mean Coverage | % of bases above 5000X | iPSC clones | Mean Coverage | % of bases above 5000X | NPC clones mean coverage | % of bases above 5000X |
|---|---|---|---|---|---|---|---|
| **C1** | 16,215 X | 100 | 19M | 10.433 X | 94.4 | | |
| | | | 38M | 8,331 X | 84.5 | | |
| | | | 44M | 7,055 X | 72.7 | 4,057 X | 51 |
| **C2** | 16,559 X | 100 | #37 | 1,087 X | 0 | 5,531 X | 47.1 |
| | | | #68 | 6,653 X | 56.1 | 5,534 X | 60 |
| **C3** | 7,641 X | 80.5 | #7 | 7,274 X | 76.5 | | |
| **C4** | 7,874 X | 78.7 | #123 | 8,437 X | 90 | | |
| | | | #130 | 8,220 X | 61.5 | | |
| **C5** | 7,740 X | 89.5 | #105 | 9,135 X | 96.3 | | |
| **M1** | 14,751 X | 100 | #20 | 7,282 X | 49.2 | 7,231 X | 82 |
| | | | #32 | 8,798 X | 94.2 | | |
| **M2** | 15,206 X | 100 | 15M | 6,785 X | 75.4 | 6,230 X | 50 |
| | | | 33M | 6,095 X | 65.5 | | |
| **M3** | 18,298 X | 100 | #A | 6,171 X | 49.2 | | |
| | | | #B | 5,923 X | 48.4 | | |
| | | | #L | 5,904 X | 63.2 | | |
| | | | #N | 2,726 X | 16 | | |
| **M4** | 18,917 X | 100 | A1 | 7,189 X | 72.8 | 7,417 X | 85.3 |
| **M5** | 7,537 X | 68.5 | A2 | 6,271 X | 56.3 | 7,002 X | 82 |
| **M6** | 16,034 X | 100 | A3 | 7,138 X | 74.2 | 6,618 X | 70.8 |
| **N1** | 14,648 X | 100 | 1535 | 5,970 X | 45.6 | 1,913 X | 0 |
| **N2** | 19,002 X | 100 | 13M | 8,458 X | 90.4 | 4,074 X | 27.6 |
| | | | 20M | 1,857 X | 0 | | |
| **N3** | 6,453 X | 68.3 | #202 | 6,238 X | 75.3 | | |
| | | | #205 | 5,711 X | 67.4 | | |
| **N4** | 15,839 X | 100 | #12 | 1,136 X | 0 | 1,655 X | 0 |
| | | | #18 | 6,857 X | 67.2 | 3,776 X | 13.7 |
| **N5** | 17,048 X | 100 | #72 | 7,793 X | 66.6 | 12,425 X | 85.2 |
| | | | #75 | 8,388 X | 49.8 | 7,232 X | 75.7 |
| **N6** | 5,698 X | 89.9 | #2 | 15,998 X | 98.3 | | |

| | | | | | | | |
|---|---|---|---|---|---|---|---|
| | | | #3 | 14,770 X | 97.9 | | |
| | | | #4 | 11,107 X | 94.2 | 6,114 X | 77 |
| | | | #6 | 11,723 X | 95.7 | | |
| | | | #8 | 13,095 X | 97.3 | 7,229 X | 86.3 |
| **N7** | 10,640 X | 96.3 | #34 | 5,606 X | 49.5 | | |

**Table S1. mtDNA NGS coverage.**

| POSITION | REF | ALT | LOCUS | AA CHANGE | tRNA | MitoTIP | DISEASE SCORE | POLYPHEN2 | WHO |
|---|---|---|---|---|---|---|---|---|---|
| 183 | A | G | MT-DLOOP | | | | | | N6 |
| 811 | G | A | MT-RNR1 | | | | | | M3 |
| 1489 | G | A | MT-RNR1 | | | | | | C4 |
| 1562 | G | A | MT-RNR1 | | | | | | M6 |
| 1693 | C | A | MT-RNR2 | | | | | | N2 |
| 1693 | C | A | MT-RNR2 | | | | | | M6 |
| 1975 | T | C | MT-RNR2 | | | | | | M3 |
| 2256 | T | C | MT-RNR2 | | | | | | C4 |
| 2404 | T | C | MT-RNR2 | | | | | | N4 |
| 2522 | T | C | MT-RNR2 | | | | | | N1 |
| 2766 | C | A | MT-RNR2 | | | | | | M5 |
| 2895 | T | C | MT-RNR2 | | | | | | N6 |
| 3009 | C | A | MT-RNR2 | | | | | | N4 |
| 3243 | A | G | MT-TL1 | | 14;0;DL;A;Y | pathogenic | | | M3 |
| 3399 | A | G | MT-ND1 | Syn | | | | | M3 |
| 3404 | T | C | MT-ND1 | L33P | | | 0.84 | 0.991 | N6 |
| 4244 | G | A | MT-ND1 | S313N | | | 0.11 | 0.0 | N4 |
| 4933 | T | C | MT-ND2 | L155P | | | 0.857 | 0.99 | C1 |
| 5068 | T | A | MT-ND2 | M200K | | | 0.417 | 0.184 | N1 |
| 5107 | C | T | MT-ND2 | T213I | | | 0.244 | 0.101 | N4 |
| 5293 | G | A | MT-ND2 | S275N | | | 0.189 | 0 | M1 |
| 5610 | G | A | MT-TA | | 50;II;TS;C;N | possibly benign | | | N4 |
| 6018 | G | A | MT-CO1 | A39T | | | 0.14 | 0.015 | N6 |
| 7824 | C | T | MT-CO2 | S80F | | | 0.835 | 0.913 | N4 |
| 8537 | A | G | MT-ATP8 | I58V | | | 0.341 | 0.326 | M5 |
| 10158 | T | C | MT-ND3 | S34P | | | 0.397 | 0.202 | M4 |
| 10377 | C | T | MT-ND3 | Syn | | | | | C3 |
| 10861 | T | C | MT-ND4 | Syn | | | | | N2 |
| 11150 | G | A | MT-ND4 | A131T | | | 0.161 | 0.035 | M1 |
| 11154 | T | C | MT-ND4 | I132T | | | 0.72 | 0.99 | C2 |
| 11157 | T | C | MT-ND4 | I133T | | | 0.794 | 0.99 | M4 |
| 11299 | T | C | MT-ND4 | Syn | | | | | N1 |
| 11361 | T | C | MT-ND4 | M201T | | | 0.709 | 0.949 | M3 |

| | | | | | | | | | |
|---|---|---|---|---|---|---|---|---|---|
| 11511 | A | G | MT-ND4 | N251S | | | 0.153 | 0 | C2 |
| 11546 | G | A | MT-ND4 | V263M | | | 0.137 | 0 | C1 |
| 11835 | G | T | MT-ND4 | W359L | | | 0.73 | 0.995 | M4 |
| 12049 | C | T | MT-ND4 | Syn | | | | | M4 |
| 12100 | A | G | MT-ND4 | Syn | | | | | M4 |
| 12371 | T | C | MT-ND5 | L12P | | | O.502 | na | C2 |
| 12721 | C | A | MT-ND5 | L129M | | | 0.62 | 0.965 | M2 |
| 12778 | G | A | MT-ND5 | Stop-gain | | | | | M3 |
| 12926 | A | G | MT-ND5 | D197G | | | 0.485 | 0.65 | N5 |
| 13020 | A | C | MT-ND5 | Syn | | | | | N3 |
| 13424 | T | C | MT-ND5 | L363P | | | 0.857 | 0.995 | M4 |
| 13425 | C | A | MT-ND5 | Syn | | | | | N3 |
| 13759 | G | A | MT-ND5 | A475P | | | 0.08 | 0 | M2 |
| 14750 | A | G | MT-CYB | T2A | | | 0.051 | 0 | M3 |
| 14761 | C | T | MT-CYB | Syn | | | | | N5 |
| 15293 | T | C | MT-CYB | F183L | | | 0.74 | 0.999 | M4 |
| 15541 | T | C | MT-CYB | Syn | | | | | M4 |
| 15959 | G | A | MT-TP | | 70;II;AS;C;N | possibly benign | | | N2 |
| 16044 | T | C | MT-DLOOP | | | | | | N2 |

**Table S2. Variants transmitted from fibroblasts to iPSCs clones.**

| POSITION | REF | ALT | LOCUS | AA CHANGE | tRNA | MitoTIP | DISEASE SCORE | POLYPHEN2 | WHO |
|---|---|---|---|---|---|---|---|---|---|
| 183 | A | G | MT-DLOOP | | | | | | N6 |
| 1562 | G | A | MT-RNR1 | | | | | | M6 |
| 2404 | T | C | MT-RNR2 | | | | | | N4 |
| 2766 | C | A | MT-RNR2 | | | | | | M5 |
| 3009 | C | A | MT-RNR2 | | | | | | N4 |
| 3010 | G | A | MT-RNR2 | | | | | | N2 |
| 4244 | G | A | MT-ND1 | S313N | | | 0.11 | 0 | N4 |
| 4933 | T | C | MT-ND2 | L155P | | | 0.857 | 0.99 | C1 |
| 5293 | G | A | MT-ND2 | S275N | | | 0.189 | 0 | M1 |
| 5610 | G | A | MT-TA | | 50;II;TS;C;N | possibly benign | | | N4 |
| 5654 | T | C | MT-TA | | 2;II;AS;A;N | possibly benign | | | N6 |
| 7592 | C | T | MT-CO2 | H3Y | | | 0.718 | 0.991 | M6 |
| 7824 | C | T | MT-CO2 | S80F | | | 0.835 | 0.913 | N4 |
| 8537 | A | G | MT-ATP8 | I58V | | | 0.341 | 0.326 | M5 |
| 10695 | G | A | MT-ND4L | A76T | | | 0.74 | 0.931 | M6 |
| 11150 | G | A | MT-ND4 | A131T | | | 0.161 | 0.035 | M1 |
| 11299 | T | C | MT-ND4 | Syn | | | | | N1 |
| 11511 | A | G | MT-ND4 | N251S | | | 0.153 | 0 | C2 |
| 11546 | G | A | MT-ND4 | V263M | | | 0.137 | 0 | C1 |
| 11835 | G | T | MT-ND4 | W359L | | | 0.73 | 0.995 | M4 |
| 12049 | C | T | MT-ND4 | Syn | | | | | M4 |
| 12100 | A | G | MT-ND4 | Syn | | | | | M4 |
| 12371 | T | C | MT-ND5 | L12P | | | 0.502 | No Info | C2 |
| 12649 | C | A | MT-ND5 | L105M | | | 0.67 | 1 | C2 |
| 12721 | C | A | MT-ND5 | L129M | | | 0.62 | 0.965 | M2 |
| 12926 | A | G | MT-ND5 | D197G | | | 0.485 | 0.65 | N5 |
| 13575 | C | T | MT-ND5 | Syn | | | | | N5 |
| 13759 | G | A | MT-ND5 | A475T | | | 0.08 | 0 | M2 |

| | | | | | | | | | |
|---|---|---|---|---|---|---|---|---|---|
| 13813 | G | A | MT-ND5 | V493I | | | 0.14 | 0.002 | N6 |
| 14424 | A | G | MT-ND6 | S84P | | | 0.832 | 1 | M2 |
| 14761 | C | T | MT-CYB | Syn | | | | | N5 |
| 15293 | T | C | MT-CYB | F183L | | | 0.74 | 0.999 | M4 |
| 15541 | T | C | MT-CYB | Syn | | | | | M4 |
| 15959 | G | A | MT-TP | | 70;II;AS;C;N | possibly benign | | | N2 |
| 16254 | A | G | MT-DLOOP | | | | | | M5 |

**Table S3. Variants transmitted from iPSCs to NPCs clones.**

| POSITION | REF | ALT | HF | LOCUS | AA CHANGE | tRNA | MitoTIP | DISEASE SCORE | POLYPHEN2 | WHO | GB |
|---|---|---|---|---|---|---|---|---|---|---|---|
| 185 | G | A | 6% | MT-DLOOP | | | | | | N5 #72 | 3445 |
| 228 | G | A | 5% | MT-DLOOP | | | | | | N5 #72 | 1901 |
| 537 | C | T | 8% | MT-DLOOP | | | | | | M3 A | 114 |
| 722 | C | T | 6% | MT-RNR1 | | | | | | C3 #7 | 62 |
| 1388 | C | A | 2.9% | MT-RNR1 | | | | | | N6 #4 | NA |
| 1573 | A | G | 2.9% | MT-RNR1 | | | | | | C5 #105 | NA |
| 2297 | A | T | 0.7% | MT-RNR2 | | | | | | N6 #3 | NA |
| 2643 | G | A | 2% | MT-RNR2 | | | | | | N3 #205 | 2 |
| 3010 | G | A | 0.6% | MT-RNR2 | | | | | | N2 13M | 7223 |
| 3110 | C | T | 4.3% | MT-RNR2 | | | | | | N7 #34 | 10 |
| 3123 | G | A | 7% | MT-RNR2 | | | | | | C4 #130 | NA |
| 3281 | G | A | 32% | MT-TL1 | | 50;0;TS;G;N | possibly benign | | | N6 #6 | 2 |
| 4596 | G | A | 1.4% | MT-ND2 | V43I | | | 0.12 | 0.006 | N6 #3 | NA |
| 5654 | T | C | 4.4% | MT-TA | | 2;II;AS;A;N | possibly benign | | | N6 #8 | 5 |
| 6486 | C | A | 15% | MT-CO1 | L195I | | | 0.73 | 0.99 | N6 #4 | NA |
| 7002 | C | A | 1.1% | MT-CO1 | L367M | | | 0.75 | 0.999 | N6 #2 | NA |
| 7026 | G | A | 1.5% | MT-CO1 | A375T | | | 0.87 | 1.0 | C5 #105 | NA |
| 7412 | C | T | 11% | MT-CO1 | syn | | | | | M5 A2 | 11 |
| 7592 | C | T | 16% | MT-CO2 | H3Y | | | 0.718 | 0.991 | M6 A3 | 1 |
| 7687 | C | A | 1.5% | MT-CO2 | I34M | | | 0.77 | 0.969 | N7 #34 | NA |
| 7970 | T | A | 2% | MT-CO2 | E129K | | | 0.13 | 0.0 | C5 #105 | NA |
| 8815 | C | T | 8% | MT-ATP6 | Stop-gain | | | | | N3 #205 | NA |
| 9163 | G | A | 3.4% | MT-ATP6 | V213I | | | 0.81 | 0.975 | C5 #105 | 58 |
| 9715 | G | A | 4% | MT-CO3 | G170D | | | 0.842 | 0.999 | N3 #202 | NA |
| 10695 | G | A | 17% | MT-ND4L | A76T | | | 0.74 | 0.931 | M6 A3 | 2 |
| 12192 | G | A | 2.2% | MT-TH | | 59;II;TL;G;N | likely benign | | | N6 #3 | 112 |
| 12649 | C | A | 2% | MT-ND5 | L105M | | | 0.67 | 1.0 | C2 #37 | NA |

| | | | | | | | | | | | |
|---|---|---|---|---|---|---|---|---|---|---|---|
| 13575 | C | T | 8%<br>7% | MT-ND5 | syn | | | | | N5 #72<br>N5 #75 | 24 |
| 13813 | G | A | 3% | MT-ND5 | V493I | | | 0.14 | 0.002 | N6 #8 | 22 |
| 14004 | C | T | 2.5% | MT-ND5 | syn | | | | | N6 #6 | 7 |
| 14424 | A | G | 34%<br>16% | MT-ND6 | S84P | | | 0.832 | 1.0 | M2 #15<br>M2 #33 | 1 |
| 16019 | C | A | 3% | MT-TP | | 5;II;AS;G;N | possibly benign | | | N6 #4 | NA |
| 16240 | A | G | 1.3% | MT-DLOOP | | | | | | C5 #105 | 358 |
| 16254 | A | G | 12% | MT-DLOOP | | | | | | M5 A2 | 229 |

**Table S4. *Unique* variants of iPSCs clones.**

| POSITION | REF | ALT | HF | LOCUS | AA CHANGE | tRNA | MitoTIP | DISEASE SCORE | POLYPHEN2 | WHO | GB |
|---|---|---|---|---|---|---|---|---|---|---|---|
| 438 | C | T | 8.8% | MT-DLOOP | | | | | | N2 13M | 15 |
| 456 | C | T | 4.6% | MT-DLOOP | | | | | | C2 #68 | 1911 |
| 765 | C | T | 18.9% | MT-RNR1 | | | | | | N1 1535 | NA |
| 991 | G | C | 1% | MT-RNR1 | | | | | | N5 #72 | NA |
| 1693 | C | A | 2.8%<br>1.9% | MT-RNR2 | | | | | | N6 #4<br>N6 #8 | NA |
| 3243 | A | G | 1.2% | MT-TL1 | | 14;;DL;A;Y | confirmed pathogenic | | | C2 #68 | 9 |
| 5366 | C | T | 9.4% | MT-ND2 | syn | | | | | M5 A2 | 3 |
| 5917 | G | A | 2.2% | MT-CO1 | R5H | | | 0.76 | 0.999 | M6 A3 | NA |
| 6512 | T | C | 1% | MT-CO1 | syn | | | | | N5 #75 | 8 |
| 6610 | G | A | 3.1% | MT-CO1 | Stop-gain | | | | | N6 #4 | NA |
| 6641 | T | C | 0.7% | MT-CO1 | syn | | | | | N5 #75 | 40 |
| 7337 | G | A | 2.3% | MT-CO1 | syn | | | | | N2 13M | 589 |
| 8007 | G | A | 0.9% | MT-CO2 | R141Q | | | 0.8 | 1.0 | N6 #4 | NA |
| 9311 | T | C | 1.6% | MT-CO3 | syn | | | | | N2 13M | 47 |
| 11443 | A | G | 0.9% | MT-ND4 | syn | | | | | C2 #68 | 4 |
| 11788 | C | T | 1% | MT-ND4 | syn | | | | | M2 15M | 33 |
| 11827 | T | C | 0.5% | MT-ND4 | syn | | | | | N6 #8 | 80 |
| 11969 | G | A | 1.5% | MT-ND4 | A404T | | | 0.26 | 0.002 | C2 #68 | 742 |
| 12549 | C | A | 2% | MT-ND5 | syn | | | | | N6 #4 | NA |
| 12561 | G | A | 1.5% | MT-ND5 | syn | | | | | N6 #4 | 230 |
| 13011 | C | G | 4.2% | MT-ND5 | syn | | | | | N6 #4 | NA |
| 13395 | A | G | 1.3% | MT-ND5 | syn | | | | | C2 #68 | 337 |
| 14438 | G | A | 4.2% | MT-ND6 | P79L | | | 0.91 | 1.0 | N5 #72 | NA |
| 14560 | G | A | 1.8% | MT-ND6 | syn | | | | | N5 #72 | 1140 |
| 14710 | G | A | 3.5% | MT-TE | | 36;II;CL;C;N | confirmed pathogenic | | | N5 #72 | NA *Encephalomyopathy + Retinopathy |

| | | | | | | | | | | | |
|---|---|---|---|---|---|---|---|---|---|---|---|
| 15100 | C | T | 7% | MT-CYB | syn | | | | | M1 #20 | 16 |
| 15643 | C | T | 1% | MT-CYB | syn | | | | | N4 #18 | 9 |
| 15758 | A | G | 3.4% | MT-CYB | I338V | | | 0.15 | 0.001 | N2 13M | 389 |
| 16148 | C | T | 2.5% | MT-DLOOP | | | | | | N6 #4 | 3350 |
| 16304 | T | C | 3% | MT-DLOOP | | | | | | C2 #68 | 11367 |
| 16400 | C | T | 1.7% | MT-DLOOP | | | | | | C2 #68 | 322 |

**Table S5. *Unique* variants in NPCs clones.**

| POSITION | REF | ALT | HF | LOCUS | AA CHANGE | tRNA | MitoTIP | DISEASE SCORE | POLYPHEN2 | WHO | GB |
|---|---|---|---|---|---|---|---|---|---|---|---|
| 1082 | A | G | 12% | MT-RNR1 | | | | | | N4 | NA |
| 1489 | G | A | 0.5% | MT-RNR1 | | | | | | C2 | NA |
| 1578 | A | G | 1.1% | MT-RNR1 | | | | | | N7 | NA |
| 1816 | G | A | 2.3% | MT-RNR2 | | | | | | N3 | 1 |
| 2256 | T | C | 2.6% | MT-RNR2 | | | | | | C2 | NA |
| 2338 | A | G | 1.2% | MT-RNR2 | | | | | | N6 | NA |
| 2778 | T | C | 1.8% | MT-RNR2 | | | | | | N7 | NA |
| 2819 | G | A | 6.1% | MT-RNR2 | | | | | | N7 | NA |
| 3193 | T | C | 2.2% | MT-RNR2 | | | | | | N6 | NA |
| 3248 | G | A | 2.4% | MT-TL1 | | 18;0;DL;G;Y | possibly benign | | | N7 | 1 |
| 3628 | A | G | 25.3% | MT-ND1 | T108A | | | 0.135 | 0.001 | M4 | NA |
| 4059 | C | T | 4.9% | MT-ND1 | syn | | | | | N7 | 57 |
| 4445 | T | C | 55.4% | MT-TM | | 48;II;VL;U;Y | possibly benign | | | N4 | NA |
| 5296 | T | C | 25.1% | MT-ND2 | L276P | | | 0.69 | 0.988 | N6 | NA |
| 5540 | G | A | 5.6% | MT-TW | | 30;II;CS;G;N0.65 | possibly pathogenic | | | C2 | NA *Encephalomyopathy/ DEAF |
| 5970 | G | A | 6% | MT-CO1 | G23S | | | 0.26 | 0.04 | C2 | 3 |
| 6261 | G | A | 2.3% | MT-CO1 | A120T | | | 0.664 | 0.988 | N3 | 361 |
| 6382 | G | A | 55.2% | MT-CO1 | G160E | | | 0.869 | 0.992 | N4 | NA |
| 8728 | T | C | 7.4% | MT-ATP6 | W68R | | | 0.874 | 0.999 | M3 | NA |
| 8817 | A | G | 21.5% | MT-ATP6 | syn | | | | | N4 | 54 |
| 8993 | T | C | 19.6% | MT-ATP6 | L156P | | | 0.902 | 0.999 | M2 | 2 *NARP/Leigh Disease /MILS /other |
| 9753 | G | A | 8% | MT-CO3 | E183K | | | 0.76 | 0.983 | N7 | 1 |
| 9906 | G | A | 15.7% | MT-CO3 | G234S | | | 0.758 | 1.0 | N5 | NA |
| 10863 | G | A | 6.9% | MT-ND4 | S35N | | | 0.762 | 0.995 | M5 | 3 |
| 11340 | T | C | 12.9% | MT-ND4 | L194S | | | 0.699 | 0.978 | M3 | NA |
| 12040 | A | C | 3.2% | MT-ND4 | K427N | | | 0.7 | 0.994 | N7 | 11 |
| 12460 | T | C | 40.5% | MT-ND5 | S42P | | | 0.711 | 0.978 | M2 | NA |
| 14384 | G | A | 9.1% | MT-ND6 | A97V | | | 0.073 | 0.0 | N4 | 83 |

| | | | | | | | | | | | |
|---|---|---|---|---|---|---|---|---|---|---|---|
| 15441 | T | C | 1% | MT-CYB | L232S | | | 0.66 | 0.981 | C5 | NA |
| 15612 | G | A | 13.9% | MT-CYB | G289E | | | 0.844 | 1.0 | M3 | NA |
| 15898 | T | C | 3.8% | MT-TT | | 11;II;DS;U;N | possibly pathogenic | | | N7 | 1 |
| 16390 | G | A | 9.7% | MT-DLOOP | | | | | | N5 | 7404 |

**Table S6. Variants non-transmitted from fibroblasts to iPSCs.**

| POSITION | REF | ALT | HF | LOCUS | AA_CHANGE | tRNA | MitoTIP | DISEASE SCORE | POLYPHEN2 | WHO | GB |
|---|---|---|---|---|---|---|---|---|---|---|---|
| **185** | G | A | 5.5% | MT-DLOOP | | | | | | N5 #72 | 3445 |
| **228** | G | A | 5.2% | MT-DLOOP | | | | | | N5 #72 | 1901 |
| **1388** | C | A | 2.9% | MT-RNR1 | | | | | | N6 #4 | NA |
| **1693** | C | A | 5.6%<br>9% | MT-RNR2 | | | | | | M6 A3;<br>N2 13M | NA |
| **2522** | T | C | 9% | MT-RNR2 | | | | | | N1 1535 | NA |
| **5068** | T | A | 8% | MT-ND2 | M200K | | | 0.417 | 0.184 | N1 1535 | NA |
| **5107** | C | T | 6% | MT-ND2 | T213I | | | 0.244 | 0.101 | N4 #12 | NA |
| **6486** | C | A | 1.5% | MT-CO1 | L195I | | | 0.73 | 0.99 | N6 #4 | NA |
| **7412** | C | T | 11.3% | MT-CO1 | syn | | | | | M5 A2 | 11 |
| **10158** | T | C | 0.2% | MT-ND3 | S34P | | | 0.397 | 0.202 | M4 A1 | NA |
| **10861** | T | C | 0.3% | MT-ND4 | syn | | | | | N2 13M | 42 |
| **11154** | T | C | 0.3% | MT-ND4 | I132T | | | 0.72 | 0.99 | C4 #68 | NA |
| **11157** | T | C | 0.2% | MT-ND4 | I133T | | | 0.794 | 0.99 | M4 A1 | 2 |
| **13424** | T | C | 0.2% | MT-ND5 | L363P | | | 0.857 | 0.995 | M4 A1 | NA |
| **16019** | C | A | 3% | MT-TP | | 5;II;AS;G;N | possibly benign | | | N6 #4 | NA |
| **16044** | T | C | 0.3% | MT-DLOOP | | | | | | N2 13M | 5 |

**Table S7. Variants non-transmitted from iPSCs to NPCs.**

| POSITION | REF | ALT | HF | WHO iPSC | DEPTH | FORWARD | REVERSE |
|---|---|---|---|---|---|---|---|
| 2297 | A | T | 0.7% | N6 #3 | 16359 | 7985 | 8374 |
| 2643 | G | A | 2% | N3 #205 | 5774 | 2805 | 2969 |
| 3010 | G | A | 0.6% | N2 13M | 10821 | 5220 | 5601 |
| 4596 | G | A | 1.4% | N6 #3 | 12618 | 6066 | 6552 |
| 7002 | C | A | 1.1% | N6 #2 | 17465 | 7040 | 10425 |
| 7026 | G | A | 1.5% | C5 #105 | 12452 | 5048 | 7404 |
| 7687 | C | A | 1.5% | N7 #34 | 3615 | 1605 | 2010 |
| 7970 | T | A | 2% | C5 #105 | 9320 | 5279 | 4041 |
| 12649 | C | A | 2% | C2 #37 | 1237 | 571 | 666 |
| **POSITION** | **REF** | **ALT** | **HF** | **WHO NPC** | **DEPTH** | **FORWARD** | **REVERSE** |
| 991 | G | C | 1% | N5 #72 | 5772 | 3086 | 2686 |
| 1693 | C | A | 1.9% | N6 #6-#8 | 8154 | 4812 | 3342 |
| 3243 | A | G | 1.2% | C2 #68 | 4260 | 1957 | 2303 |
| 6512 | T | C | 1% | N5 #75 | 11985 | 6789 | 5196 |
| 6641 | T | C | 0.7% | N5 #75 | 13436 | 7282 | 6154 |
| 8007 | G | A | 0.9% | N6 #4 | 6026 | 2920 | 3106 |
| 9311 | T | C | 1.6% | N2 13M | 5891 | 2603 | 3288 |
| 11443 | A | G | 0.9% | C2 #68 | 5953 | 3028 | 2925 |
| 11788 | C | T | 1% | M2 15M | 14260 | 6871 | 7389 |
| 11827 | T | C | 0.5% | N6 #8 | 11247 | 5127 | 6120 |
| 11969 | G | A | 1.5% | C2 #68 | 5185 | 2447 | 2738 |
| 12549 | C | A | 2% | N6 #4 | 5804 | 2903 | 2901 |
| 12561 | G | A | 1.5% | N6 #4 | 5514 | 2595 | 2919 |
| 13395 | A | G | 1.3% | C2 #68 | 4672 | 2233 | 2439 |
| 14560 | G | A | 1.8% | N5 #72 | 10719 | 5947 | 4772 |
| 15643 | C | T | 1% | N4 #18 | 6378 | 3380 | 2998 |
| 16400 | C | T | 1.7% | C2 #68 | 11278 | 6161 | 5117 |

**Table S8. Coverage and reads strand orientation of *unique* variants with HF≤2% in iPSCs and NPCs.**
