# Supplementary material for: The relevance of mitochondrial DNA variants fluctuation during reprogramming and neuronal differentiation of human iPSCs
Source: Stem Cell Reports. 2021 Jul 29;16(8):1953–67. doi: 10.1016/j.stemcr.2021.06.016 (PMC8365099; doi:10.1016/j.stemcr.2021.06.016)
Supplement: Document S2. Article plus supplemental information [file mmc2.pdf]

Stem Cell Reports
Article

OPEN ACCESS

The relevance of mitochondrial DNA variants fluctuation during reprogramming and neuronal differentiation of human iPSCs

Flavia Palombo,[1,11] Camille Peron,[2,11] Leonardo Caporali,[1] Angelo Iannielli,[3] Alessandra Maresca,[1] Ivano Di Meo,[2] Claudio Fiorini,[1,4] Alice Segnali,[2] Francesca L. Sciacca,[2] Ambra Rizzo,[2] Sonia Levi,[3,5] Anu Suomalainen,[6,7,8] Alessandro Prigione,[9] Vania Broccoli,[3,10] Valerio Carelli,[1,4] and Valeria Tiranti[2,*]
[1]IRCCS Istituto delle Scienze Neurologiche di Bologna, Programma di Neurogenetica, Bologna 40139, Italy
[2]Fondazione IRCCS Istituto Neurologico Carlo Besta, Milan 20133, Italy
[3]Division of Neuroscience, San Raffaele Scientific Institute, Milan 20132, Italy
[4]Department of Biomedical and NeuroMotor Sciences (DIBINEM), University of Bologna, Bologna 40123, Italy
[5]Vita-Salute San Raffaele University, Milan 20132, Italy
[6]Stem Cell and Metabolism Research Program Unit, Faculty of Medicine, University of Helsinki, Helsinki 00014, Finland
[7]Neuroscience Institute, HiLife, University of Helsinki, Helsinki 00014, Finland
[8]HUSLab, Helsinki University Hospital, Helsinki 00014, Finland
[9]Department of General Pediatrics, Neonatology and Pediatric Cardiology, Duesseldorf University Hospital, Medical Faculty, Heinrich Heine University, Duesseldorf 40225, Germany
[10]National Research Council (CNR), Institute of Neuroscience, Milan 20132, Italy
[11]These authors contributed equally
*Correspondence: valeria.tiranti@istituto-besta.it
https://doi.org/10.1016/j.stemcr.2021.06.016

SUMMARY

The generation of inducible pluripotent stem cells (iPSCs) is a revolutionary technique allowing production of pluripotent patient-specific cell lines used for disease modeling, drug screening, and cell therapy. Integrity of nuclear DNA (nDNA) is mandatory to allow iPSCs utilization, while quality control of mitochondrial DNA (mtDNA) is rarely included in the iPSCs validation process. In this study, we performed mtDNA deep sequencing during the transition from parental fibroblasts to reprogrammed iPSC and to differentiated neuronal precursor cells (NPCs) obtained from controls and patients affected by mitochondrial disorders. At each step, mtDNA variants, including those potentially pathogenic, fluctuate between emerging and disappearing, and some having functional implications. We strongly recommend including mtDNA analysis as an unavoidable assay to obtain fully certified usable iPSCs and NPCs.

INTRODUCTION

The exponential increase of applications of induced pluripotent stem cells (iPSCs) includes generation of differentiated cells, development of organoids for investigations of disease mechanisms and drug discovery (Shi et al., 2017), and their clinical use for therapeutic purposes in humans (Barker et al., 2017). This poses specific questions on their quality control, and there are concerns about age-related burden of somatic mutations (Kang et al., 2016; Lo Sardo et al., 2017), lineage-specific epigenetic memory affecting the methylation pattern (Nashun et al., 2015), and pre-existing or reprogramming-related occurrence of tumorigenic mutations (Ben-David and Benvenisty, 2011; Gore et al., 2011). Such concerns have recently hampered and currently limit the therapeutic use of autologous iPSCs (Garber, 2015).

One area of genetic variability affecting the quality of iPSCs is mitochondrial DNA (mtDNA), a small, circular, multicopy, double-stranded molecule of DNA within mitochondria, which has a mutagenesis rate much higher than nuclear DNA (Gustafsson et al., 2016; Wallace, 2015). The mtDNA hosts hundreds of pathogenic mutations causing a vast variety of clinical phenotypes (La Morgia et al., 2020) characterized by defective oxidative phosphorylation (OXPHOS), increased oxidative stress, calcium mishandling, propensity to apoptosis, altered organelle dynamics, and removal by autophagy (Nunnari and Suomalainen, 2012). Furthermore, mtDNA genetics follows peculiar rules: maternal inheritance, homo-hetero-polyplasmy, mitotic segregation and threshold effect, germline bottleneck, and individual cell clonal expansion of mutant molecules. All these mtDNA features are reflected in higher-order complexities when it comes to global function of individual cells, including iPSCs (Xu et al., 2013). Of particular relevance is the potential functional reflection on iPSCs of mtDNA sequence diversity characterizing human populations (Gómez-Durán et al., 2010; Strobbe et al., 2018).

A few studies clearly highlighted the major impact that mtDNA mutagenesis may have on iPSC quality (Kang et al., 2016; Lorenz and Prigione, 2016; Perales-Clemente et al., 2016; Prigione et al., 2011; Zambelli et al., 2018). Critical issues are the clonal expansion in single cells of private heteroplasmic mtDNA variants as part of the so-called universal heteroplasmy (Payne et al., 2013), the possible *de novo* mtDNA mutagenesis occurring during cycling of the parental cell line, the possible mtDNA mutagenesis related

This is an open access article under the CC BY license (http://creativecommons.org/licenses/by/4.0/).

to cell reprogramming, and the bottleneck effects and genetic drifts due to the mtDNA copy number reduction characterizing iPSCs (Bukowiecki et al., 2014; Xu et al., 2013). All these possibilities remain open questions, and previous studies have reached contradictory results. The introduction of deep next-generation sequencing (NGS) has lowered the detection limit of heteroplasmy to 0.2%, and different studies demonstrated that low-level heteroplasmy (down to 0.1%) may be transmitted and maintained within families (Giuliani et al., 2014; Guo et al., 2013).

We present our own analysis of iPSCs generated from fibroblasts and peripheral blood mononuclear cells (PBMCs) and, for the first time, of neuronal precursor cells (NPCs). We performed mtDNA deep sequencing in fibroblasts/iPSCs/NPCs obtained from controls and two classes of patients: a mitochondrial group carrying known homoplasmic or heteroplasmic mtDNA mutations and a nuclear group carrying mutations in nuclear genes coding for mitochondrial proteins.

RESULTS

Deep sequencing of mtDNA

We performed mtDNA deep sequencing in 17 fibroblast cell lines, one PBMC cell line, 35 iPSCs clones, and 16 NPCs, belonging to the three categories: controls (C1 to C5) (Figure S1A), mitochondrial (M1 to M6) (Figure S1B), and nuclear (N1 to N7) patients (Figure S1C) (Table 1). A median coverage of 14,978X (±4,731X), 7,138X (±3,269X), and 6,172X (±2,551X) was respectively achieved in fibroblasts/PBMCs, iPSCs, and NPCs (Table S1). In some cases, we observed a coverage reduction for certain mtDNA regions and we checked for the presence of macrodeletions by analyzing the sequencing data with the MitoSAlt tool (https://sourceforge.net/projects/mitosalt/) (Basu et al., 2020). No macrodeletions were observed with heteroplasmy levels higher than 1%. This result was further supported by the analysis with digital droplet PCR, on the same cell lines carrying *OPA1* mutations here investigated and previously published (Iannielli et al., 2018).

Variants were analyzed with PhyloTree (https://www.phylotree.org/tree/index.htm) to reconstruct the haplogroup of each cell line and the full consistency of haplotypes between parental fibroblasts, iPSCs derived clones, and correlated NPCs (Table 1). All haplogroups were common to the population of European ancestry (H, J, U), except one control cell line (C5) carrying the D1g haplotype. We analyzed only private single nucleotide mtDNA variants, not belonging to the specific haplogroup. We traced their segregation during reprogramming and differentiation to assess the change in heteroplasmy fraction (HF) and screened for possible novel damaging variants.

Impact of age, reprogramming methods, and mitochondrial haplotype on abundance of mtDNA variants in fibroblasts, iPSCs, and NPCs

In our study, the age range of subjects was wide (12–80 years) and we investigated the impact of age on the number of mtDNA variants. The average number of variants in fibroblasts from older subjects ($\geq$50 years old) was significantly higher compared with younger subjects (Figure 1A). Moreover, there was a linear correlation (Figure 1B) between ages and somatic mtDNA variability accumulated in fibroblasts. In contrast, we did not observe a greater number of mtDNA variants (Figure 1A) either in the iPSCs or in the NPCs in the older group. Similarly, the age-related variants burden was progressively lesser and gradually dropped in each cell stage (Figure 1B).

We here employed three reprogramming procedures: Sendai virus in 14 cell lines, retroviral transduction in three lines, and an episomal plasmid in the remaining one (M4, Table 1). We did not observe a significant effect of a specific reprogramming method on the number of mtDNA heteroplasmic variants either in iPSCs or in NPCs (Figure 1C).

We also evaluated the effect of mitochondrial haplotypes, specifically J, H, and U, on the burden of mtDNA variants, excluding the D1g haplotype, present in a single cell line. The number of variants in fibroblasts and NPCs was not related to a specific haplotype, while haplotype J turned out to have significantly fewer variants in iPSCs (Figure 1D).

mtDNA heteroplasmy and variants segregations from parental fibroblasts to iPSCs

The analysis of private variants showed a high frequency of heteroplasmy in the three groups for a total of 120 heteroplasmic variants; the mitogenomes of the patients' groups did not harbor a higher number of variants in both fibroblasts ($p = 0.082$) and iPSCs ($p = 0.234$) (not shown). Moreover, in iPSCs, we observed a wide range of segregation models of the fibroblast variants (Figures S3 and S4): some iPSC clones completely lost the parental fibroblast variants, presenting (Figures S2G, S3D, S4C, and S4K) or not (Figures S2A and S4B) their own unique variants. Other iPSC clones harbored entirely (Figures S2F and S4A) or in part (Figures S2A, S2B, S2D, S3B, S3E, S3F, S4D, and S4E) only the variants present in their parental fibroblasts. However, the majority of iPSC clones contained both parental fibroblasts and unique variants in variable numbers and combinations (Figures 2A–2C). Although the highest variability was observed in the iPSCs carrying the *POLG* p.P648R mutation (N6), these did not display a significantly higher number of variants compared with the iPSCs derived from control, mitochondrial, or nuclear groups (Figure S5A).

We observed a general increase of heteroplasmy levels, which, in some cases, led variants heteroplasmic in

Table 1. Features of subjects in control, mitochondrial, and nuclear groups

Patient Age	Haplogroup	Phenotype (#MIM)	Gene	Nt change	AA change	Reprogram system	Starting material	iPSC clones	NPC clones	CGH/Karyotype iPSC clones	Reference
**Control group**											
C1 34	J1c2	none	none	none	none	Sendai virus	fibroblasts	19M	0	0.4-Mb duplication in 18p11.22	
								38M	0	normal	
								44M	1	normal	
C2 54	H4a1a	none	none	none	none	Sendai virus	fibroblasts	#37	1	normal	
								#68	1	normal	
C3 32	H1	none	none	none	none	Sendai virus	fibroblasts	#7	0	1.5-Mb deletion in 16q23.3	
C4 54	H4a1a	none	none	none	none	Sendai virus	fibroblasts	#123	0	normal	
								#130	0	normal	
C5 39	D1g	none	none	none	none	Sendai virus	PBMCs	#105	0	normal	
**Mitochondrial group**											
M1 26	J1c4	LHON (#535000)	MT-ND4	m.11778G>A homoplasmic	p.R340H	Sendai virus	fibroblasts	#20	1	normal	
								#32	0	normal	
M2 19	H1at1	LHON (#535000)	MT-ND1	m.3460G>A homoplasmic	p.A52T	Sendai virus	fibroblasts	15M	1	60.6-Mb duplication in 1p35.3p22.2	
								33M	0	normal	Peron et al., (2020)
M3 40	H7	MELAS (#540000)	MT-TL1	m.3243A>G heteroplasmic	NA	retroviral transduction	fibroblasts	#A	0	normal	Hämäläinen et al., (2013)
								#B	0	normal	
								#L	0	normal	
								#N	0	normal	
M4 80	H1b	NARP (#551500)	MT-ATP6	m.9185T>C homoplasmic	p.L220P	episomal plasmid	fibroblasts	A1	1	normal	Auré et al. (2013) Lorenz et al., (2017)
M5 47	H1b	NARP (#551500)	MT-ATP6	m.9185T>C homoplasmic	p.L220P	retrovirus	fibroblasts	A2	1	normal	
M6 20	H1b	NARP (#551500)	MT-ATP6	m.9185T>C homoplasmic	p.L220P	retrovirus	fibroblasts	A3	1	normal	

(*Continued on next page*)

Table 1. *Continued*

Patient Age	Haplogroup	Phenotype (#MIM)	Gene	Nt change	AA change	Reprogram system	Starting material	iPSC clones	NPC clones	CGH/Karyotype iPSC clones	Reference
**Nuclear group**											
N1 12	U5a1a1	PKAN (#234200)	PANK2	c.569insA homozygous	p.Y190X	Sendai virus	fibroblasts	1535	1	normal	Orellana et al., (2016)
N2 20	U4b1a	DOA (#165500)	OPA1	c.1334 G>A heterozygous	p.R445H	Sendai virus	fibroblasts	13M	1	normal	
								20M	0	normal	
N3 27	H13a1a1	DOA (#165500)	OPA1	c.1334 G>A heterozygous	p.R445H	Sendai virus	fibroblasts	#202	0	normal	
								#205	0	normal	
N4 69	HV0b	DOA (#165500)	OPA1	c.1462A>G heterozygous	p.G488R	Sendai virus	fibroblasts	#12	1	normal	Iannielli et al., (2018)
								#18	1	normal	
N5 70	H1aq	DOA (#165500)	OPA1	c.1484C>T heterozygous	p.A495V	Sendai virus	fibroblasts	#72	1	normal	
								#75	1	normal	
N6 57	H1ab1	CPEO (#203700)	POLG	c.1943C>G homozygous	p.P648R	Sendai virus	fibroblasts	#2	0	normal	
								#3	0	normal	
								#4	1	normal	
								#6	0	normal	
								#8	1	normal	
N7 75	J1c15	CPEO (#609286)	TWNK	c.907C>T heterozygous	p.R303W	Sendai virus	fibroblasts	#34	0	normal	

MIM, Online Mendelian Inheritance in Man; Nt, nucleotide; AA, amino acid; CPEO, chronic progressive external ophtalmoplegia

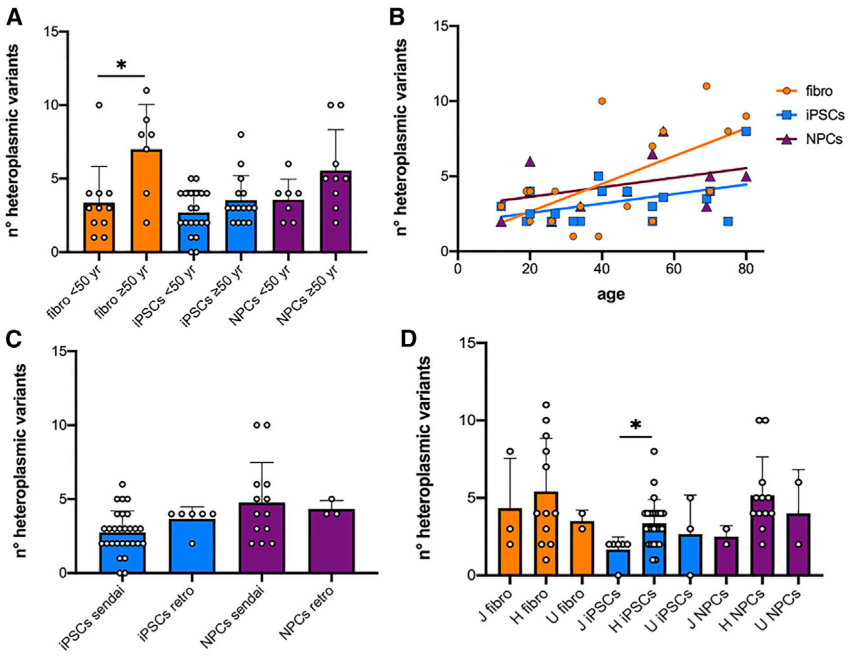

Figure 1. Effect of age, reprogramming methods, and mitochondrial haplotype on the number of mtDNA heteroplasmic variants in fibroblasts, iPSCs, and NPCs

(A) Elderly subjects presented higher number of heteroplasmic variants in fibroblasts (p = 0.0132), but not in iPSCs (p = 0.135) and NPCs (p = 0.10).

(B) The linear correlation between ages and the accumulation of heteroplasmic variants was statistically significant only in fibroblasts (adjusted R-squared = 0.376, p = 0.0068). Adjusted R-squared = 0.196, p = 0.065 in iPSCs; adjusted R-squared = 0.151, p = 0.236 in NPCs.

(C) Number of heteroplasmic variants was independent of the reprogramming method in iPSCs (p = 0.08) and NPCs (p = 0.97).

(D) Haplotype J had significantly fewer variants in iPSCs (p = 0.031). Number of variants in fibroblasts (p = 0.796) and NPCs (p = 0.241) was not related to a specific haplotype.

fibroblasts to become homoplasmic in iPSCs as in C3 #7 (m.10377C>T from 23% to 99%–100%), M1 #32 (m.11150G>A from 53% to 95%), N5 #72-#75 (from 48% to 98%–100%), and N6 #2-#4-#8 (from 21% to 100%) (Figures 2A–2C). This incremental shift of heteroplasmy was also observed for the heteroplasmic pathogenic MELAS (mitochondrial encephalomyopathy, lactic acidosis, and stroke-like episodes) m.3243A>G mutation. In fact, iPSC clones derived from M3 fibroblasts, showing a 24% mutation load of the MELAS allele, displayed a broad distribution of the HF with one clone 100% wild-type (A), and others ranging from 41% to 81% (B, L, and N) of the mutant allele (Figure 2B, variant in magenta). However, exceptions to this general increase were observed in M1 #20 (m.11150G>T from 53% to 0.65%; Figure S3A), in M4 A1 (m.10158T>C from 24% to 0.2%, m.11157T>C from 25% to 0.2%, and m.13424T>C from 28% to 0.2%; Figure S3F), in N2 13M (m.10861T>C from 14% to 0.3% and m.16044T>C from 6% to 0.3%; Figure S4B) and in N3 #205 (m.13020T>C from 11% to 1%; Figure S4C). In contrast, the Leber hereditary optic neuropathy (LHON) m.11778G>A in M1 and m.3460G>A in M2, and the neuropathy ataxia retinitis pigmentosa (NARP) m.9185T>C in M4/M5/M6 remained homoplasmic in all iPSC clones as in the original fibroblasts.

mtDNA heteroplasmy and variants segregations from iPSCs to NPCs

Sixteen iPSC clones, three in the control, five in the mitochondrial, and eight in the nuclear groups (Table 1), were differentiated in NPCs and checked for mtDNA private variants heteroplasmy. Globally, 86 heteroplasmic variants were observed in iPSCs/NPCs and the three groups did not differ in terms of variants number (p = 0.676; not shown). The large majority of the variants were shared between iPSCs and NPCs: only one NPC clone (#72 N5) presented just its own unique variants (Figure S4F), while other NPCs were more heterogeneous (#68 C2, 13M N2, and #4 N6) sharing two, two, and one variants with the iPSCs of origin, respectively (Figures S2D, S4B, and S4I).

NPCs variants usually presented an HF similar to that of the iPSCs of origin, and even in the case of increment/reduction this was of limited magnitude and consistent with the direction of heteroplasmy change previously observed in the reprogramming step (Figures 3A–3C). A few exceptions were noted, as in M1 NPC #20 holding a variant with HF increasing from 0.65% to 85%, whereas in M4 NPC A1 the HF increase was less marked (from 6% to 11%); both variants were predicted to be benign. Moreover, C1 and N6 NPCs showed, respectively, a private benign variant in the *MT-TI* tRNA gene (6% HF) and a synonymous variant in the *MT-CYB* gene (0.6% HF), present in the parental fibroblasts at extremely low HF (0.7%), but apparently absent in their corresponding iPSCs (Figures S2B and S4J).

mtDNA localization and prediction of variants transmitted in reprogramming and differentiating step

Fifty-two out of 86 variants, with HF ranging from 0.2% to 100%, were transmitted during the reprogramming step,

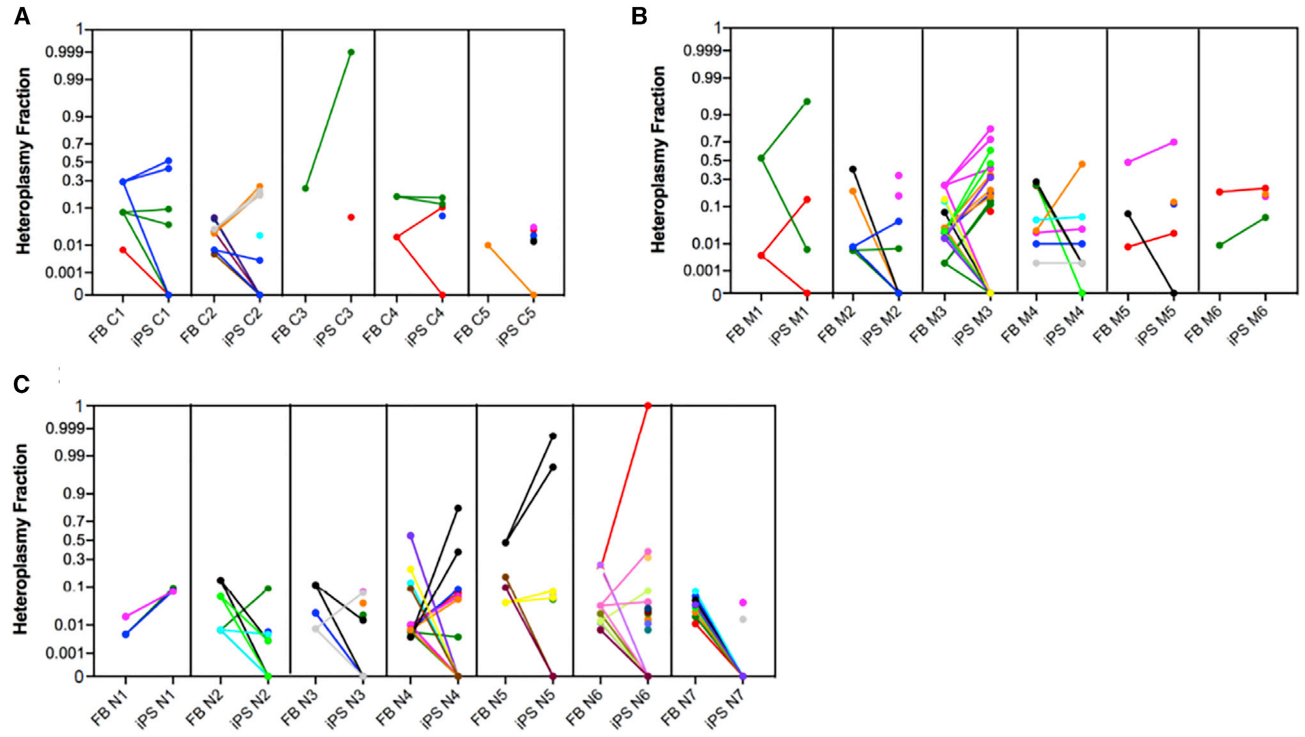

Figure 2. Fibroblasts to iPSCs heteroplasmy shift

Variants' HF fluctuations during the reprogramming step from fibroblasts to iPSCs in control (A), mitochondrial (B), and nuclear (C) groups. Dots appearing in iPSC mean unique variants. Non-transmitted variants are highlighted with a straight line going to zero in iPSC. One color represents one variant.

without any obvious bias among the three groups (p = 0.3) (Table S2). Specifically, eight variants were in the control, 23 in the mitochondrial, and 21 in the nuclear groups. We observed 35 variants in protein-coding genes, 12 rRNA genes, three in tRNA genes, but only two in the D-loop region (Figures 4A and S5B–S5D). *MT-ND4*, *MT-ND5*, and *MT-RNR2*, the three largest mtDNA genes, presented the greater number of variants, whereas *MT-ATP6*, *MT-CO3*, *MT-ND4L*, and *MT-ND6* did not show variants; *MT-TA*, *MT-TL1*, and *MT-TP* were the only tRNA genes carrying variants (Figure 4B). The variants' transmission seemed to be independent from their pathogenicity in both control and patient groups. Most of the transmitted variants were predicted to be benign (24 of 38) and were in cells from affected patients (Figures 4C and S5E–S5G), but neither benign (p = 0.251) nor damaging (p = 0.428) nor non-coding (p = 0.809) variants were preferentially transmitted during the reprogramming step.

In the differentiating step, 35 variants ranging from HF 0.2%–100%, out of 52, were transmitted from iPSCs to NPCs: five in the control, 16 in the mitochondrial, and 14 in the nuclear groups (Table S3). The variants' transmission was unaffected by the control/patient status of the NPCs (p = 0.74), and most of the variants were located in protein-coding genes. The remaining variants were in non-coding genes: two in the D-loop region, five in rRNA, and three in tRNA genes (Figures 4D and S5H–S5J). The majority of transmitted variants were in *MT-ND4* and *MT-ND5* genes, and all protein-coding genes, except *MT-ATP6*, *MT-CO1*, *MT-CO3*, and *MT-ND3*, presented with variants; *MT-TA* and *MT-TP* were the only tRNA genes carrying variants (Figure 4E). Even in iPSC to NPC transition, despite 17 out of 28 variants being predicted to be benign (Figure 4F and S5K–S5M), there was no difference in the transmission of benign (p = 0.559), likely damaging (p = 0.285), or non-coding (p = 0.621) variants in the three groups.

Analysis of variants unique in iPSCs and NPCs

The variant m.7824C>T in *MT-CO2*, causing the amino acid change p.S80F predicted to be highly damaging (disease score 0.835 and Polyphen2 score 0.913), had high heteroplasmy levels (81% and 37%), in N4 iPSC clones #12 and #18 and derived NPC (72% and 47%), and was already present at only 0.4% in N4 parental fibroblasts (Figures S4D and S4E). Therefore, we focused on iPSCs unique variants, comprising both somatic variants and variants arising during reprogramming. We counted 34 unique variants: eight in the control, six in the mitochondrial, and 20 in the

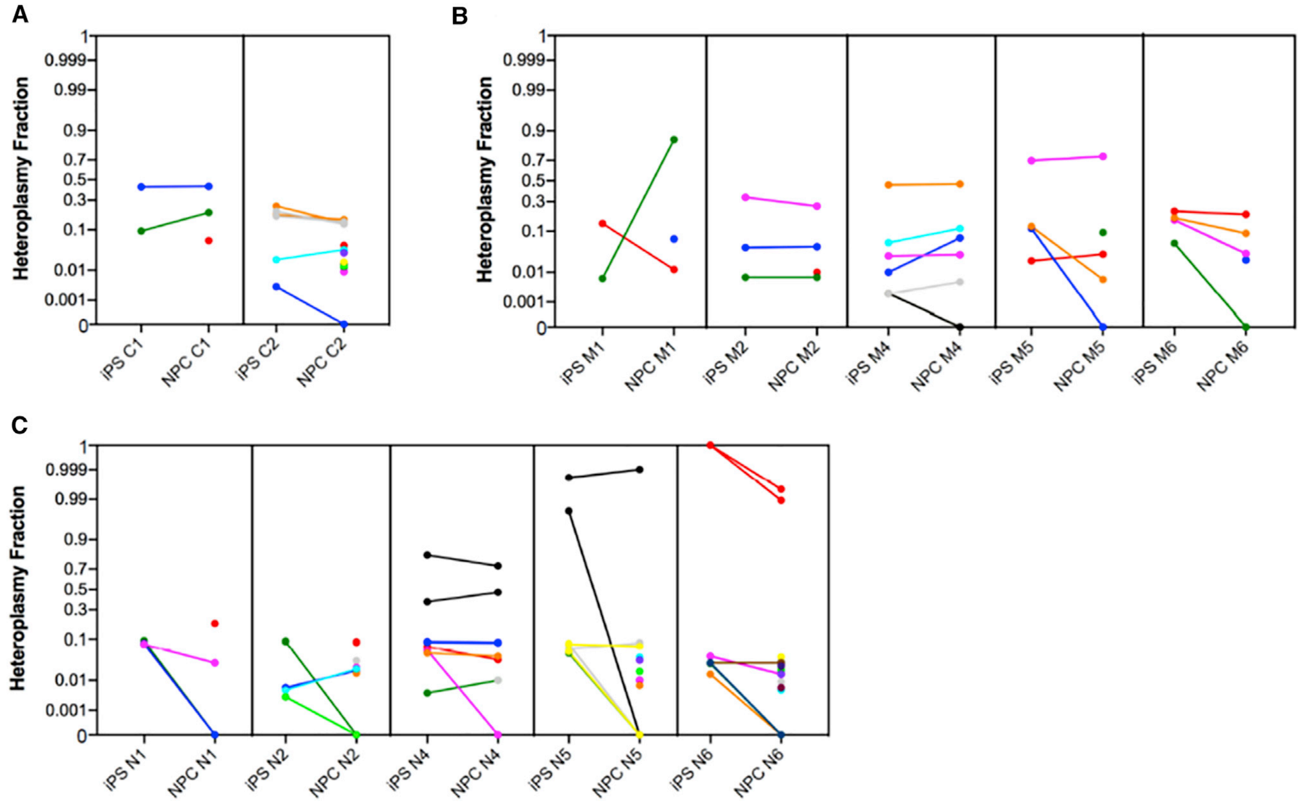

Figure 3. iPSCs to NPCs heteroplasmy shift

Variants' HF fluctuations during the differentiation step from iPSCs to NPCs in control (A), mitochondrial (B), and nuclear (C) groups. Dots appearing in NPC mean unique variants. Non-transmitted variants are highlighted with a straight line going to zero in NPC. One color represents one variant.

nuclear groups (Table S4). Although the average number of unique variants was not dependent on the presence of wild-type or mutated mitochondrial/nuclear DNA in the starting cell lines (p = 0.096), we noticed the highest variability in the N6 iPSC clone. Then, we tested whether these *POLG*-derived iPSCs accumulated more unique variants compared with all other iPSCs, and we found a statistically significant difference (Figure S5N). Five variants were in the D-loop region, eight in rRNA genes, four in tRNA, and 17 in protein-coding genes (Figure 5A). *MT-DLOOP* and *MT-RNR2* resulted in more variants, while *MT-ATP8*, *MT-CYB*, *MT-ND1*, *MT-ND3*, and *MT-ND4* did not have heteroplasmic variability; only *MT-TA*, *MT-TH*, *MT-TL1*, and *MT-TP* tRNA genes showed the presence of variants (Figure 5B). Annotation of the protein-coding variants revealed that one was a stop-gain, three were synonymous, while 13 were nonsynonymous, 10 of which were predicted to be deleterious (Figure 5C). However, we noticed that the nature of a variant (p = 0.213 for the non-coding variants) or its predicted pathogenicity (p = 0.888 and p = 0.920, respectively, for the benign and damaging variants) did not drive the occurrence as a unique variant in the three groups. Finally, interrogation of the GenBank database showed that 14 variants were never reported, whereas the most represented variants in the database were in the D-loop region and in rRNA genes (Table S4).

NPCs accumulated 31 unique variants (HF from 0.5% to 18.9%): seven in the control, four in the mitochondrial, and 20 in the nuclear groups (Table S5). We did not notice a significant difference in the average number between the three groups (p = 0.26), even though roughly half of the variants of the nuclear group were contributed by the N6 (*POLG*) patient. Five variants were located in the D-loop region, two in tRNA, three in rRNA genes, and 21 in protein-coding genes (Figure 5D). *MT-DLOOP* and *MT-CO1* accumulated more variants, while we did not observe variants in *MT-ATP6*, *MT-ATP8*, *MT-ND1*, *MT-ND3*, and *MT-ND4L* genes; *MT-TE* and *MT-TL1* were the only tRNA genes with variants (Figure 5E). Notably, the MELAS m.3243A>G mutation arose, at low heteroplasmy (1%), in C2 #68. Finally, also for NPC unique variants, notwithstanding that 17 out of 23 were predicted to be benign (Figure 5F), we failed to

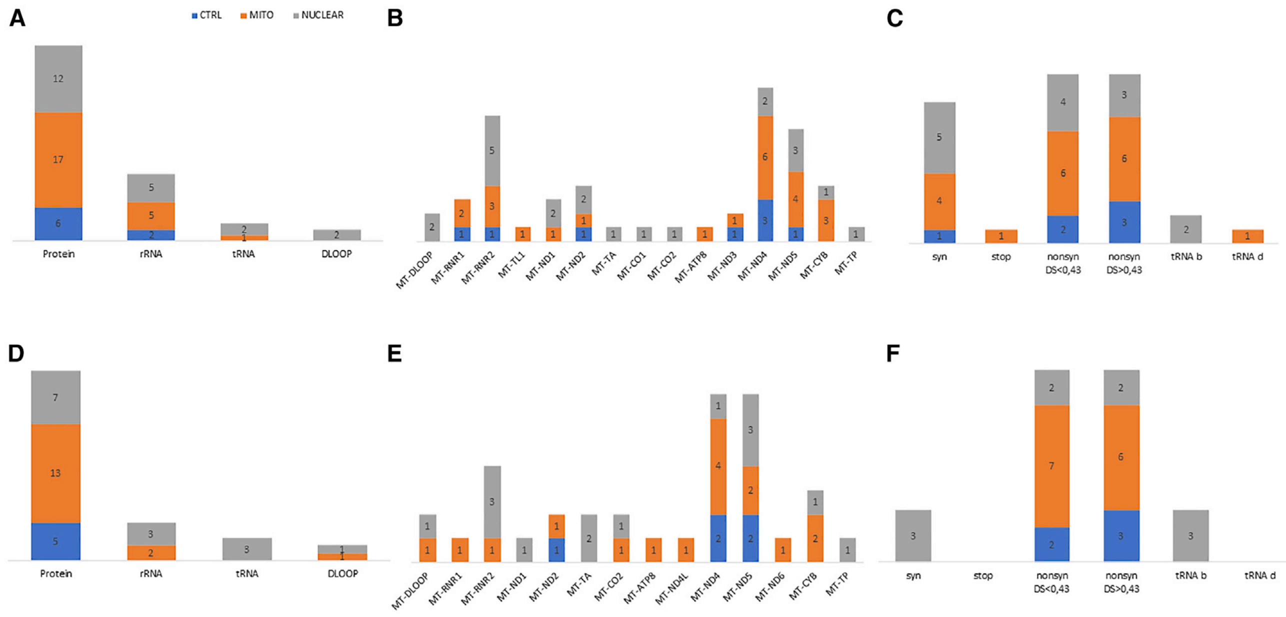

Figure 4. mtDNA localization and prediction of variants transmitted in reprogramming and differentiating steps in the three groups

(A) Fifty-two heteroplasmic variants transmitted during the reprogramming step.
(B) Mitochondrial genes localization of transmitted variants in iPSCs.
(C) Predictions of transmitted variants in iPSCs.
(D) Thirty-five heteroplasmic variants transmitted in the differentiating step.
(E) Mitochondrial genes localization of transmitted variants in NPCs.
(F) Predictions of transmitted variants in NPCs.
syn, synonymous; nonsyn, nonsynonymous; b, benign; d, damaging.

observe a significant difference in the occurrence of unique benign (p = 0.402), damaging (p = 0.767), or non-coding (p = 0.114) variants between controls and patients' groups. Ten variants, located in the *MT-DLOOP* and rRNA, were never reported in GenBank (Table S5).

Analysis of non-transmitted variants in iPSCs and NPCs

Thirty-two fibroblast variants (HF range 0.5%–55%) were non-transmitted in iPSC clones in a comparable way between groups (p = 0.357): five in the control, seven in the mitochondrial, and 20 in the nuclear groups (Table S6). One variant was in the D-loop region; nine in rRNA genes, with the 16s rRNA resulting in the gene non-transmitting the largest number of variants; four in the tRNA; and 18 in protein-coding genes, of which 13 were nonsynonymous predicted to likely be damaging (Figures S6A–S6C). In particular, two reported pathogenic variants, m.5540G>A with 5% HF (Bannwarth et al., 2013; Silvestri et al., 2000) and m.8993T>C with 19.6% HF (Kara et al., 2012; Weerasinghe et al., 2018), did not pass from C2 and M2 fibroblasts to iPSCs. Although 15 out of 22 non-transmitted variants were predicted as damaging, we noticed a statistical difference only for the non-transmitted non-coding variants among the mitochondrial and the nuclear groups (p = 0.02), whereas there was no difference for benign (p = 0.853) and damaging (p = 0.767) variants.

During the differentiation step, 16 variants with HF ranging from 0.2% to 11% were non-transmitted: one in the control, five in the mitochondrial, and 11 in the nuclear groups; the last two groups shared a variant in *MT-RNR2* (m.1693C>A) (Table S7). Importantly, also in this step, we did not observe a bias in the non-transmission of the variants among the three groups (p = 0.521). Eight variants were in non-coding regions in tRNA/rRNA genes (three in the D-loop region, one in a tRNA, and four in rRNA genes) (Figures S6D and S6E), whereas nine affected protein-coding genes, including four nonsynonymous variants predicted as deleterious (Figure S6F). Neither non-coding (p = 0.188), nor benign (p = 0.909), nor damaging (p = 0.086) variants were preferentially non-transmitted.

Functional studies of mtDNA mutations in NPCs

To verify functional consequences of predicted pathogenic mtDNA mutations we used, as a paradigm, NPCs derived from iPSCs carrying variable levels of the p.S80F mutation

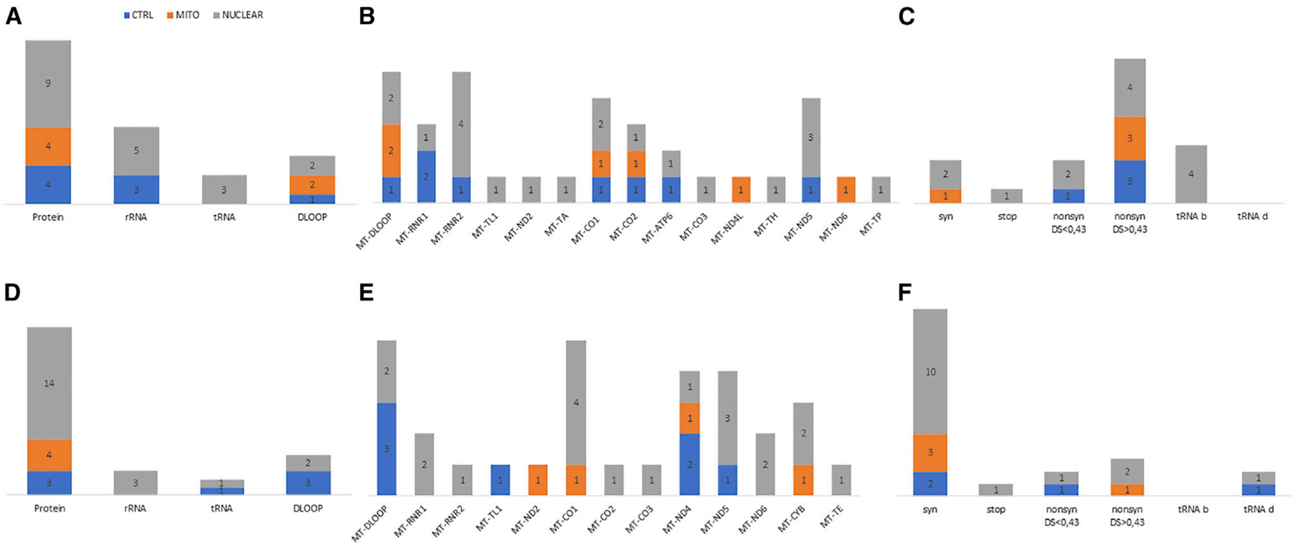

Figure 5. mtDNA localization and prediction of unique variants in iPSCs and NPCs in the three groups
(A) Thirty-four heteroplasmic variants were unique in iPSCs clones.
(B) Mitochondrial genes localization of transmitted variants in iPSCs.
(C) Predictions of unique variants in iPSCs.
(D) Thirty-one heteroplasmic variants were unique in the NPCs clones.
(E) Mitochondrial genes localization of unique variants in NPCs.
(F) Predictions of unique variants in NPCs.
syn, synonymous; nonsyn, nonsynonymous; b, benign; d, damaging.

(m.7824C>T) in the *MT-CO2* subunit gene of cytochrome c oxidase (COX), predicted to be highly pathogenic and never reported in Mitomap. We tested two independent NPCs (#12 and #18) obtained from patient N4, with 73% or 47% HF. Oxygen consumption rate showed a significant reduction of the maximal respiratory rate in NPC clone #12, and to a lesser extent in clone #18, as compared with control NPCs or NPCs derived from two clones (G488R#12 and G488R#22), generated from a different patient carrying an *OPA1* mutation identical (Iannielli et al., 2018) to the one present in patient N4, but with a wild-type mtDNA sequence (Figure 6).

DISCUSSION

Appearance of mtDNA variants during iPSCs reprogramming and their differentiation into specific lineages is a sensible area of investigation for the experimental and therapeutic use of iPSCs. We showed that, at each step from parental fibroblasts/PBMCs, to reprogrammed iPSCs, and then differentiated NPCs, HF of mtDNA variants, including those potentially pathogenic, fluctuate between emerging or disappearing or undergoing drift. The dynamics of these fluctuations may include bottleneck events but also genetic drifts in the absence of a clear purifying selection.

We performed deep mtDNA sequencing in several cell types, obtained from both control individuals and patients with mitochondrial diseases. The high coverage obtained, especially in fibroblasts, allowed us to confidently identify variants with a very low heteroplasmic load, missed by standard sequencing technologies, demonstrating the existence of universal mtDNA heteroplasmy, due to both inherited and somatic variants (Payne et al., 2013; Wei et al., 2019). We showed that low heteroplasmic variants (<1%) can be considered with high confidence true variants since those present with an extremely low HF (from 0.2% to 1%) in fibroblasts were identified in derived iPSCs and/or NPCs and unique variants were shared among different iPSCs or NPCs, indicating their probable origin from the parental fibroblasts. Fibroblasts are pooled cells with high mtDNA sequence heterogeneity and certain variants, even present at high HF in a single cell, ultimately appear diluted or even absent in the cell culture. Thus, we might consider as an initial bottleneck event the clonal nature of iPSCs reprogramming from a single fibroblast, highlighting its parental heteroplasmic profile. Ultimately, the mtDNA variants observed in the iPSCs may reflect either true *de novo* events or pre-existing heteroplasmic variants buried under the detection threshold in the parental fibroblasts, but expanding in the clonally reprogrammed cells. Congruently, it was shown that MELAS iPSC clones shifted

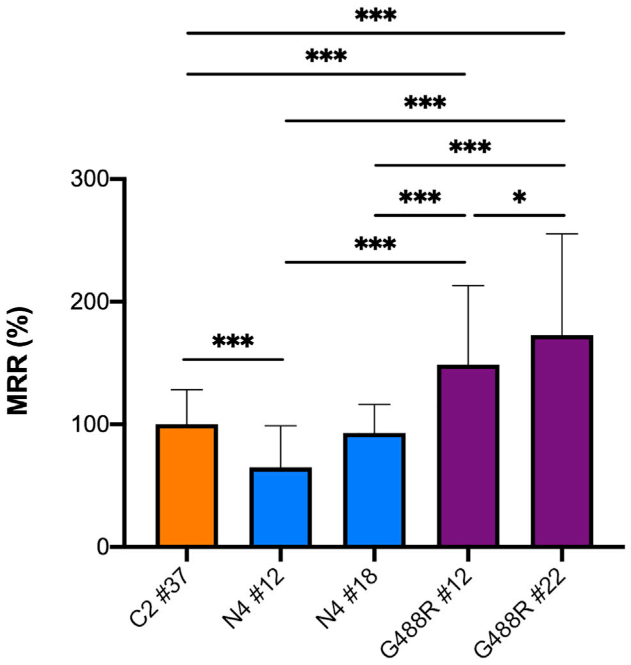

Figure 6. MRR of NPC
Control (C2 #37), *OPA1* p.G488R mutation with (N4 #12 and N4 #18) or without (G488R #12 and G488R #22; Iannielli et al., 2018) p.S80F mutation in CO2 mtDNA sequence. Control = 100% MRR. *$p < 0.05$, ***$p < 0.001$. All measurements were performed in 30 replicates for each sample. At least three different experiments in blind conditions to the examiner were carried out on different days.

either close to zero or to high mutant load during reprogramming, whereas fibroblast cultures showed a wide spectrum of heteroplasmy states (Hämäläinen et al., 2013). This scenario was already described and discussions revolved around the issue of whether the iPSCs variants were pre-existing and detectable in the original fibroblasts cell culture or not (Kang et al., 2016; Perales-Clemente et al., 2016).

Previous studies underlined the correlation of mtDNA variants in iPSCs with the age of patients originating the primary cell culture (Kang et al., 2016). We confirm this age-related increase of mtDNA variants in fibroblasts, as shown by simply comparing the young (aged 12–49 years) and old (aged 50–80 years) subjects. However, we failed to detect the same correlation either in iPSCs or in NPCs, even if in Kang's study there was also a consistent trend in reducing the amount of variants across reprogramming (see Figure 3E in Kang et al., 2016). The reprogramming procedure implies a sort of rejuvenation process (Goya et al., 2018) in which iPSCs derived from old individuals could reset their genetic heritage through multiple passages and, in doing so, erase the mtDNA alterations accumulated during aging in the parental fibroblasts. Our data are consistent with the number of passages of the iPSCs (15–20), whereas those analyzed by Kang had a significantly lower (2–4) number of passages (Kang et al., 2016), when iPSCs display the greatest heterogeneity and variability (Volpato and Webber, 2020). The hypothesis of germline purifying selection based on the functional impact of mtDNA mutations has been previously proposed in murine models, acting most efficiently against variants affecting protein-coding genes (Stewart et al., 2008). Our results to some extent provide the same indication of preferential selection against mtDNA mutation in protein-encoding genes. It has also been reported that the MELAS mutation, affecting a tRNA, progressively reduced its heteroplasmic load with increasing iPSCs passages in prolonged cell culture (Folmes et al., 2013). Thus, even if a purifying selection against specific damaging variants cannot be excluded, we here demonstrated that pathogenic variants are present in iPSCs and maintained in derived NPCs. This may suggest a different surveillance mechanism to explain the vanishing of age-related differences, which might go beyond mtDNA. It remains that iPSCs rely mainly on glycolysis, explaining the tolerance of damaging variants affecting the OXPHOS system. Further investigations are required to fully understand the rejuvenation or purifying selection.

The iPSCs carrying haplogroup J1c, but neither fibroblasts nor NPCs, had a significantly reduced variants number compared with the most frequent European haplogroup H. This points to something happening at the reprogramming step or in the culturing passages of iPSCs. The nuclear background or the existence of an mtDNA pathogenic mutation were irrelevant as the three haplogroup J1c cases were respectively a control and a LHON patient, in the young group, and a Twinkle patient, in the old group. Haplogroup J is solidly associated with LHON, as a background enhancing penetrance (Carelli et al., 2006; Hudson et al., 2007; Torroni et al., 1997). Haplogroup J has also been associated with longevity in an Italian population (De Benedictis et al., 1999), but not in others (Rose et al., 2001), and the JT superhaplogroup has been proposed to be protective in Parkinson disease (Hudson et al., 2013). Testing haplogroup J in cybrids highlighted a slightly inefficient OXPHOS compared with other haplogroups, in particular haplogroup H, which may enhance the pathogenicity of LHON mutations, but in the long run accumulate less somatic mutations favoring longevity and protecting from Parkinson (Gómez-Durán et al., 2012; Strobbe et al., 2018). This seems recapitulated in the acute model of iPSCs generation, as emphasized by our results of reduced propensity to accumulate mtDNA variants in haplogroup J.

Overall, most of the reported variants increased the level of heteroplasmy from fibroblast to iPSCs. Once fixed in iPSC clones, these variants were, in most cases, present and maintained at a similar heteroplasmic level in NPCs,

suggesting that, during this transition, no major modulations of mtDNA amount occur, although additional mtDNA variants may arise. This latter event was, however, mostly, but not exclusively, occurring in *POLG* mutant cells, characterized by defective mtDNA replication. The *POLG* mutation (Table 1), a homozygous p.P648R change hitting the spacer domain of the protein, potentially alters enzyme activity, processivity, DNA-binding affinity, or affects interactions with the mitochondrial single-stranded DNA-binding protein a partner protein, part of the mitochondrial "replisome" complex (Luo and Kaguni, 2005). It is therefore conceivable to observe an increase in mutation frequency in the *POLG* mutant cell line endowed with a crippled enzyme. This does not exclude the already mentioned pre-existence of buried variants emerging as apparently *de novo* variants. In fact, in fibroblasts, we failed to observe a difference in total variants load, which became apparent in terms of *de novo* variants only after reprogramming. A similar situation occurs in the single Twinkle iPSC clone, another gene involved in mtDNA maintenance. Remarkably, in *POLG*-, *Twinkle*-, and *OPA1*-derived iPSC clones, we failed to observe mtDNA deletions, usually accumulating in post-mitotic tissues of patients (Carelli and Chan, 2014).

We did not observe the presence of recurrent mtDNA variants, nor obvious mutational hot spots or genes more prone than others to genetic variation, except for the m.1693C>A variant in the *MT-RNR2*, detected in three unrelated iPSCs. This novel variant was absent in the Mitomap repository, and was not recognized by previous studies of the iPSCs mitogenome (Kang et al., 2016; Perales-Clemente et al., 2016). Most reported variants affected protein-coding genes and roughly half of them were potentially damaging. As proof of principle, we studied the p.S80F mutation in *MT-CO2*, present at extremely low heteroplasmic level in *OPA1* fibroblasts, and expanded during reprogramming in two independent iPSCs clones and derived NPCs. This mtDNA variant caused a severe impairment of the maximal respiratory capacity, which could cause, if unaware, a misinterpretation of the functional effect of the *OPA1* mutation.

Overall, we found about equal amounts of variants in structural genes encoding OXPHOS subunits and in genes dedicated to the mtDNA translation (tRNAs/rRNAs). This contrasts with the total size of the protein-encoding genes (11.3 kb), against the size of the translation-dedicated mtDNA genes (3.9 kb), which may suggest some degree of purifying selection mostly acting on protein-coding genes (Stewart et al., 2008). It remains that clearly pathogenic variants are tolerated during reprogramming and subsequent differentiation. Interestingly, there seems to be preferential occurrence of mtDNA variants hitting a few specific tRNAs and, more consistently, the *MT-RNR2*. Some of the tRNAs (*MT-TI*, *MT-TW*, *MT-TH*, *MT-TA*, *MT-TE*, *MT-TP*) are shared by Kang et al. (2016). However, it is unclear if there is some selectivity in mtDNA mutagenesis or if some degree of purifying selection is acting through reprogramming/differentiation, or if a combination of both mechanisms may occur.

Despite previous and the present studies reported the importance of analyzing both the mitochondrial and nuclear genome in iPSCs, mtDNA sequencing is rarely incorporated in the quality control check of iPSCs (Attwood and Edel, 2019; Doi et al., 2020; Doss and Sachinidis, 2019). Remarkably, the first iPSCs therapy for Parkinson disease started in 2018 (http://www.cira.kyoto-u.ac.jp/e/pressrelease/news/180730-170000.html) but the clinical grade iPSCs characterization did not seem to include mtDNA analysis. Likewise, various studies documenting mitochondrial dysfunctions in neurodegenerative diseases, such as multiple system atrophy (Monzio Compagnoni et al., 2018), amyotrophic lateral sclerosis(Dafinca et al., 2016), Parkinson disease (Little et al., 2018), and Alzheimer disease (Birnbaum et al., 2018), did not report mtDNA analysis of iPSCs. We here showed that mtDNA variants occur not only in iPSCs but also in NPCs, adding concerns when working with this *in vitro* system.

Our study remarked that generation of iPSCs is consistently affected by events of expansion/reduction or *de novo* generation of heteroplasmic variants in mtDNA, potentially deleterious, ultimately affecting the global healthiness of the iPSCs. This has profound implications for further differentiated cells or organoids (Lancaster and Knoblich, 2014) used to model diseases, but particularly for *in vivo* therapeutic use of iPSCs in humans, as for Parkinson patients (Fan et al., 2020; Stoddard-Bennett and Pera, 2020). Furthermore, it was recently shown that nonsynonymous mtDNA mutations in iPSCs may lead to neoantigens eliciting an immune response, indicating that autologous iPSCs may not be immunologically inert (Deuse et al., 2019).

In conclusion, the mtDNA sequence profile of iPSCs is an unavoidable step to ensure that these cells are suitable for modeling diseases and testing experimental treatments. A systematic study of the dynamic changes in mtDNA variants occurrence and segregation also provides a great opportunity to better understand all issues related to universal heteroplasmy. Ultimately, checking genetic stability of iPSCs and evaluating nuclear and mitochondrial genomes is of paramount importance for quality-grade iPSC-based clinical trials.

EXPERIMENTAL PROCEDURES

Genetic characterization of patients' derived fibroblasts

Fibroblasts derived from 13 patients affected by mitochondrial disorders and five healthy age-matched subjects were included in this study and obtained from the cell lines and DNA bank of pediatric

movement disorders and mitochondrial diseases of the Telethon Network of Genetic Biobanks (http://biobanknetwork.telethon.it). Patients were classified into a mitochondrial group (M1 to M6) with either homoplasmic or heteroplasmic mtDNA mutations, or a nuclear group (N1 to N7) with mutations in nuclear genes coding for mitochondrial proteins (Table1).

Generation and Characterization of iPSCs

We used either already generated or newly produced iPSCs. On average, passages of iPSCs in culture were in the range between 15 and 20. Table 1 summarizes all the relevant information.

iPSCs for NARP mutation were generated from fibroblasts derived from a three-generation family carrying the homoplasmic m.9185T>C mutation. Subjects M4, M5, and M6 in Table 1 correspond to patient A1 (patient III-6), patient A2 (daughter of A1, patient 1, IV-5), and patient A3 (third-degree cousin of A2, patient 2, V-5) respectively (Auré et al., 2013). iPSCs for patient M4 were generated by episomal plasmid, and for patients M5 and M6 by transduction with retrovirus. Characterization and mtDNA Sanger sequence analysis were performed as reported (Lorenz et al., 2017). iPSCs for MELAS mutation were generated from fibroblasts as described (Hämäläinen et al., 2013). The mutation load was determined in fibroblasts and derived iPSCs by minisequencing (Suomalainen et al., 1993). Available clones reported in Table 1 have the following heteroplasmic levels: #A = 4.2%, #B = 2.5%, #L = 79.7%, and #N7 = 2.5%. iPSCs for dominant optic atrophy (DOA) and pantothenate kinase-associated neurodegeneration (PKAN) were generated from primary fibroblasts as described (Iannielli et al., 2018; Orellana et al., 2016). New iPSCs for DOA and LHON were generated and characterized as described (Iannielli et al., 2018; Peron et al., 2020).

CGH array

Integrity of nuclear DNA was verified by array comparative genomic hybridization (CGH) as described (Peron et al., 2020).

mtDNA sequencing

Sequence analysis of the entire mtDNA molecule was performed by the NGS approach (Caporali et al., 2018). The primers used to amplify the mtDNA molecule in two segments are strategically located outside the regions involved in the generation of breakpoints underlying mtDNA deletions. The NGS libraries were constructed by Nextera XT (Illumina) and paired end sequenced on MiSeq System (Illumina), using MiSeq Reagent Kit v3 (600 cycles).

Fastq files were analyzed with MToolBox v1.1 and v.1.2 (https://github.com/mitoNGS/MToolBox) (Calabrese et al., 2014). Only mono-allelic SNVs (single nucleotide variant) with a read depth $\geq$100 and a base quality score $\geq$30 were retained. All private heteroplasmic SNVs were visually inspected with the tool IGV (Integrative Genome Viewer: https://software.broadinstitute.org/software/igv/) in order to check the variants' strand bias, and strongly unbalanced variants were discarded. Reads depths (total, forward, and reverse) for unique variants with HF $\leq$ 2% are reported in Table S8.

Nonsynonymous variants with a disease score >0.43 were predicted to be deleterious, while for tRNA variants we considered the MitoTIP prediction.

Statistical analysis

Statistical analyses were performed with GraphPad Prism 6.0. Differences between mean values of variants with respect to age of cell line donors and to reprogramming method were assessed by the Mann-Whitney unpaired t test. Univariate linear regression analysis was performed to assess the effect of age on the number of variants in the cell lines. Differences and multiple comparisons in the three groups were estimated with the Krustal-Wallis, one-way ANOVA test. A p value $\leq$0.05 was considered significant.

Determination of respiratory activity

Oxygen consumption rate (OCR) was measured in DOA and control NPCs with an XF96 Extracellular Flux Analyzer (Seahorse Bioscience, Billerica, MA). NPCs were seeded at a density of 20,000 cells/well and measurement was performed as described (Invernizzi et al., 2012). Maximal respiration rate (MRR) was calculated as percentage of control.

Data and code availability

The SRA (Sequence Read Archive) accession number for the data reported in this paper is PRJNA706687.

SUPPLEMENTAL INFORMATION

Supplemental information can be found online at https://doi.org/10.1016/j.stemcr.2021.06.016.

AUTHORS CONTRIBUTIONS

Conceptualization, V.C. and V.T.; methodology, F.P., C.P., A.I., I.D.M., A.M., L.C., S.L., A.P., A.S., and V.B.; investigation, F.P., C.P., A.I., L.C., C.F., A.S., F.L.S., and A.R.; resources, C.P., A.I., S.L., A.P., A.S., and V.B.; data curation, F.P., C.F., and L.C.; writing – original draft, F.P., C.P., V.C., and V.T.; writing – review & editing, all authors; funding acquisition, V.C., V.B., V.T.; supervision, V.C., V.B., and V.T.

DECLARATION OF INTERESTS

The authors declare no competing interests.

ACKNOWLEDGMENTS

The financial support of Mitocon, Italy, grant no. 2018-01 to V.T. and of the grant from the Italian Ministry of Health RF-2018-12366703 to V.C., V.B., and V.T. is acknowledged. C.P. is sustained by a fellowship of Associazione Luigi Comini ONLUS, Italy (http://www.luigicominionlus.org/). We would like to thank Tuula Manninen for generation of MELAS iPSC lines. We thank funding agencies Jane and Aatos Erkko Foundation and Juselius Foundation, Finland; Academy of Finland and University of Helsinki, Finland. We thank funding agencies: UMDF, United States; BMBF (AZ031L0211 and AZ01GM2002A) and University of Düsseldorf, Germany.

Received: March 14, 2021
Revised: June 25, 2021
Accepted: June 26, 2021
Published: July 29, 2021

REFERENCES

Attwood, S.W., and Edel, M.J. (2019). iPS-cell technology and the problem of genetic instability–can it ever be safe for clinical use? J. Clin. Med. *8*, 288.

Auré, K., Dubourg, O., Jardel, C., Clarysse, L., Sternberg, D., Fournier, E., Laforêt, P., Streichenberger, N., Petiot, P., Gervais-Bernard, H., et al. (2013). Episodic weakness due to mitochondrial DNA MT-ATP6/8 mutations. Neurology *81*, 1810–1818.

Bannwarth, S., Procaccio, V., Lebre, A.S., Jardel, C., Chaussenot, A., Hoarau, C., Maoulida, H., Charrier, N., Gai, X., Xie, H.M., et al. (2013). Prevalence of rare mitochondrial DNA mutations in mitochondrial disorders. J. Med. Genet. *50*, 704–714.

Barker, R.A., Parmar, M., Studer, L., and Takahashi, J. (2017). Human trials of stem cell-derived dopamine neurons for Parkinson's disease: dawn of a new era. Cell Stem Cell *21*, 569–573.

Basu, S., Xie, X., Uhler, J.P., Hedberg-Oldfors, C., Milenkovic, D., Baris, O.R., Kimoloi, S., Matic, S., Stewart, J.B., Larsson, N.-G., et al. (2020). Accurate mapping of mitochondrial DNA deletions and duplications using deep sequencing. PLoS Genet. *16*, e1009242.

Ben-David, U., and Benvenisty, N. (2011). The tumorigenicity of human embryonic and induced pluripotent stem cells. Nat. Rev. Cancer *11*, 268–277.

Birnbaum, J.H., Wanner, D., Gietl, A.F., Saake, A., Kündig, T.M., Hock, C., Nitsch, R.M., and Tackenberg, C. (2018). Oxidative stress and altered mitochondrial protein expression in the absence of amyloid-β and tau pathology in iPSC-derived neurons from sporadic Alzheimer's disease patients. Stem Cell Res. *27*, 121–130.

Bukowiecki, R., Adjaye, J., and Prigione, A. (2014). Mitochondrial function in pluripotent stem cells and cellular reprogramming. Gerontology *60*, 174–182.

Calabrese, C., Simone, D., Diroma, M.A., Santorsola, M., Guttà, C., Gasparre, G., Picardi, E., Pesole, G., and Attimonelli, M. (2014). MToolBox: a highly automated pipeline for heteroplasmy annotation and prioritization analysis of human mitochondrial variants in high-throughput sequencing. Bioinformatics *30*, 3115–3117.

Caporali, L., Iommarini, L., La Morgia, C., Olivieri, A., Achilli, A., Maresca, A., Valentino, M.L., Capristo, M., Tagliavini, F., Del Dotto, V., et al. (2018). Peculiar combinations of individually non-pathogenic missense mitochondrial DNA variants cause low penetrance Leber's hereditary optic neuropathy. PLoS Genet. *14*, e1007210.

Carelli, V., and Chan, D.C. (2014). Mitochondrial DNA: impacting central and peripheral nervous systems. Neuron *84*, 1126–1142.

Carelli, V., Achilli, A., Valentino, M.L., Rengo, C., Semino, O., Pala, M., Olivieri, A., Mattiazzi, M., Pallotti, F., Carrara, F., et al. (2006). Haplogroup effects and recombination of mitochondrial DNA: novel clues from the analysis of Leber hereditary optic neuropathy pedigrees. Am. J. Hum. Genet. *78*, 564–574.

Dafinca, R., Scaber, J., Ababneh, N., Lalic, T., Weir, G., Christian, H., Vowles, J., Douglas, A.G.L., Fletcher-Jones, A., Browne, C., et al. (2016). C9orf72 hexanucleotide expansions are associated with altered endoplasmic reticulum calcium homeostasis and stress granule formation in induced pluripotent stem cell-derived neurons from patients with amyotrophic lateral sclerosis and frontotemporal dementia. Stem Cells *34*, 2063–2078.

De Benedictis, G., Rose, G., Carrieri, G., De Luca, M., Falcone, E., Passarino, G., Bonafe, M., Monti, D., Baggio, G., Bertolini, S., et al. (1999). Mitochondrial DNA inherited variants are associated with successful aging and longevity in humans. FASEB J. *13*, 1532–1536.

Deuse, T., Hu, X., Agbor-Enoh, S., Koch, M., Spitzer, M.H., Gravina, A., Alawi, M., Marishta, A., Peters, B., Kosaloglu-Yalcin, Z., et al. (2019). De novo mutations in mitochondrial DNA of iPSCs produce immunogenic neoepitopes in mice and humans. Nat. Biotechnol. *37*, 1137–1144.

Doi, D., Magotani, H., Kikuchi, T., Ikeda, M., Hiramatsu, S., Yoshida, K., Amano, N., Nomura, M., Umekage, M., Morizane, A., et al. (2020). Pre-clinical study of induced pluripotent stem cell-derived dopaminergic progenitor cells for Parkinson's disease. Nat. Commun. *11*, 3369.

Doss, M.X., and Sachinidis, A. (2019). Current challenges of iPSC-based disease modeling and therapeutic implications. Cells *8*, 403.

Fan, Y., Winanto, N., and Ng, S.-Y. (2020). Replacing what's lost: a new era of stem cell therapy for Parkinson's disease. Transl. Neurodegener. *9*, 2.

Folmes, C.D.L., Martinez-Fernandez, A., Perales-Clemente, E., Li, X., McDonald, A., Oglesbee, D., Hrstka, S.C., Perez-Terzic, C., Terzic, A., and Nelson, T.J. (2013). Disease-causing mitochondrial heteroplasmy segregated within induced pluripotent stem cell clones derived from a patient with MELAS. Stem Cells *31*, 1298–1308.

Garber, K. (2015). RIKEN suspends first clinical trial involving induced pluripotent stem cells. Nat. Biotechnol. *33*, 890–891.

Giuliani, C., Barbieri, C., Li, M., Bucci, L., Monti, D., Passarino, G., Luiselli, D., Franceschi, C., Stoneking, M., and Garagnani, P. (2014). Transmission from centenarians to their offspring of mtDNA heteroplasmy revealed by ultra-deep sequencing. Aging (Albany NY) *6*, 454–467.

Gómez-Durán, A., Pacheu-Grau, D., López-Gallardo, E., Díez-Sánchez, C., Montoya, J., López-Pérez, M.J., and Ruiz-Pesini, E. (2010). Unmasking the causes of multifactorial disorders: OXPHOS differences between mitochondrial haplogroups. Hum. Mol. Genet. *19*, 3343–3353.

Gómez-Durán, A., Pacheu-Grau, D., Martínez-Romero, I., López-Gallardo, E., López-Pérez, M.J., Montoya, J., and Ruiz-Pesini, E. (2012). Oxidative phosphorylation differences between mitochondrial DNA haplogroups modify the risk of Leber's hereditary optic neuropathy. Biochim. Biophys. Acta *1822*, 1216–1222.

Gore, A., Li, Z., Fung, H.-L., Young, J.E., Agarwal, S., Antosiewicz-Bourget, J., Canto, I., Giorgetti, A., Israel, M.A., Kiskinis, E., et al. (2011). Somatic coding mutations in human induced pluripotent stem cells. Nature *471*, 63–67.

Goya, R.G., Lehmann, M., Chiavellini, P., Canatelli-Mallat, M., Hereñú, C.B., and Brown, O.A. (2018). Rejuvenation by cell reprogramming: a new horizon in gerontology. Stem Cell Res. Ther. *9*, 349.

Guo, Y., Li, C.-I., Sheng, Q., Winther, J.F., Cai, Q., Boice, J.D., and Shyr, Y. (2013). Very low-level heteroplasmy mtDNA variations are inherited in humans. J. Genet. Genomics *40*, 607–615.

Gustafsson, C.M., Falkenberg, M., and Larsson, N.-G. (2016). Maintenance and expression of mammalian mitochondrial DNA. Annu. Rev. Biochem. *85*, 133–160.

Hämäläinen, R.H., Manninen, T., Koivumäki, H., Kislin, M., Otonkoski, T., and Suomalainen, A. (2013). Tissue- and cell-type-specific manifestations of heteroplasmic mtDNA 3243A>G mutation in human induced pluripotent stem cell-derived disease model. Proc. Natl. Acad. Sci. U S A *110*, E3622–E3630.

Hudson, G., Carelli, V., Spruijt, L., Gerards, M., Mowbray, C., Achilli, A., Pyle, A., Elson, J., Howell, N., La Morgia, C., et al. (2007). Clinical expression of Leber hereditary optic neuropathy is affected by the mitochondrial DNA–haplogroup background. Am. J. Hum. Genet. *81*, 228–233.

Hudson, G., Nalls, M., Evans, J.R., Breen, D.P., Winder-Rhodes, S., Morrison, K.E., Morris, H.R., Williams-Gray, C.H., Barker, R.A., Singleton, A.B., et al. (2013). Two-stage association study and meta-analysis of mitochondrial DNA variants in Parkinson disease. Neurology *80*, 2042–2048.

Iannielli, A., Bido, S., Folladori, L., Segnali, A., Cancellieri, C., Maresca, A., Massimino, L., Rubio, A., Morabito, G., Caporali, L., et al. (2018). Pharmacological inhibition of necroptosis protects from dopaminergic neuronal cell death in Parkinson's disease models. Cell Rep. *22*, 2066–2079.

Invernizzi, F., D'Amato, I., Jensen, P.B., Ravaglia, S., Zeviani, M., and Tiranti, V. (2012). Microscale oxygraphy reveals OXPHOS impairment in MRC mutant cells. Mitochondrion *12*, 328–335.

Kang, E., Wang, X., Tippner-Hedges, R., Ma, H., Folmes, C.D.L., Gutierrez, N.M., Lee, Y., Van Dyken, C., Ahmed, R., Li, Y., et al. (2016). Age-related accumulation of somatic mitochondrial DNA mutations in adult-derived human iPSCs. Cell Stem Cell *18*, 625–636.

Kara, B., Arıkan, M., Maraş, H., Abacı, N., Cakıris, A., and Ustek, D. (2012). Whole mitochondrial genome analysis of a family with NARP/MILS caused by m.8993T>C mutation in the MT-ATP6 gene. Mol. Genet. Metab. *107*, 389–393.

La Morgia, C., Maresca, A., Caporali, L., Valentino, M.L., and Carelli, V. (2020). Mitochondrial diseases in adults. J. Intern. Med. *287*, 592–608.

Lancaster, M.A., and Knoblich, J.A. (2014). Generation of cerebral organoids from human pluripotent stem cells. Nat. Protoc. *9*, 2329–2340.

Little, D., Luft, C., Mosaku, O., Lorvellec, M., Yao, Z., Paillusson, S., Kriston-Vizi, J., Gandhi, S., Abramov, A.Y., Ketteler, R., et al. (2018). A single cell high content assay detects mitochondrial dysfunction in iPSC-derived neurons with mutations in SNCA. Sci. Rep. *8*, 9033.

Lo Sardo, V., Ferguson, W., Erikson, G.A., Topol, E.J., Baldwin, K.K., and Torkamani, A. (2017). Influence of donor age on induced pluripotent stem cells. Nat. Biotechnol. *35*, 69–74.

Lorenz, C., and Prigione, A. (2016). Aging vs. rejuvenation: reprogramming to iPSCs does not turn back the clock for somatic mitochondrial DNA mutations. Stem Cell Invest. *3*, 43.

Lorenz, C., Lesimple, P., Bukowiecki, R., Zink, A., Inak, G., Mlody, B., Singh, M., Semtner, M., Mah, N., Auré, K., et al. (2017). Human iPSC-derived neural progenitors are an effective drug discovery model for neurological mtDNA disorders. Cell Stem Cell *20*, 659–674.e9.

Luo, N., and Kaguni, L.S. (2005). Mutations in the spacer region of *Drosophila* mitochondrial DNA polymerase affect DNA binding, processivity, and the Balance between pol and exo function*. J. Biol. Chem. *280*, 2491–2497.

Monzio Compagnoni, G., Kleiner, G., Samarani, M., Aureli, M., Faustini, G., Bellucci, A., Ronchi, D., Bordoni, A., Garbellini, M., Salani, S., et al. (2018). Mitochondrial dysregulation and impaired autophagy in iPSC-derived dopaminergic neurons of multiple system atrophy. Stem Cell Reports *11*, 1185–1198.

Nashun, B., Hill, P.W.S., and Hajkova, P. (2015). Reprogramming of cell fate: epigenetic memory and the erasure of memories past. EMBO J. *34*, 1296–1308.

Nunnari, J., and Suomalainen, A. (2012). Mitochondria: in sickness and in health. Cell *148*, 1145–1159.

Orellana, D.I., Santambrogio, P., Rubio, A., Yekhlef, L., Cancellieri, C., Dusi, S., Giannelli, S.G., Venco, P., Mazzara, P.G., Cozzi, A., et al. (2016). Coenzyme A corrects pathological defects in human neurons of PANK2-associated neurodegeneration. EMBO Mol. Med. *8*, 1197–1211.

Payne, B.A.I., Wilson, I.J., Yu-Wai-Man, P., Coxhead, J., Deehan, D., Horvath, R., Taylor, R.W., Samuels, D.C., Santibanez-Koref, M., and Chinnery, P.F. (2013). Universal heteroplasmy of human mitochondrial DNA. Hum. Mol. Genet. *22*, 384–390.

Perales-Clemente, E., Cook, A.N., Evans, J.M., Roellinger, S., Secreto, F., Emmanuele, V., Oglesbee, D., Mootha, V.K., Hirano, M., Schon, E.A., et al. (2016). Natural underlying mtDNA heteroplasmy as a potential source of intra-person hiPSC variability. EMBO J. *35*, 1979–1990.

Peron, C., Mauceri, R., Cabassi, T., Segnali, A., Maresca, A., Iannielli, A., Rizzo, A., Sciacca, F.L., Broccoli, V., Carelli, V., et al. (2020). Generation of a human iPSC line, FINCBi001-A, carrying a homoplasmic m.G3460A mutation in MT-ND1 associated with Leber's hereditary optic neuropathy (LHON). Stem Cell Res. *48*, 101939.

Prigione, A., Lichtner, B., Kuhl, H., Struys, E.A., Wamelink, M., Lehrach, H., Ralser, M., Timmermann, B., and Adjaye, J. (2011). Human induced pluripotent stem cells harbor homoplasmic and heteroplasmic mitochondrial DNA mutations while maintaining human embryonic stem cell-like metabolic reprogramming. Stem Cells *29*, 1338–1348.

Rose, G., Passarino, G., Carrieri, G., Altomare, K., Greco, V., Bertolini, S., Bonafè, M., Franceschi, C., and De Benedictis, G. (2001). Paradoxes in longevity: sequence analysis of mtDNA haplogroup J in centenarians. Eur. J. Hum. Genet. *9*, 701–707.

Shi, Y., Inoue, H., Wu, J.C., and Yamanaka, S. (2017). Induced pluripotent stem cell technology: a decade of progress. Nat. Rev. Drug Discov. *16*, 115–130.

Silvestri, G., Mongini, T., Odoardi, F., Modoni, A., deRosa, G., Doriguzzi, C., Palmucci, L., Tonali, P., and Servidei, S. (2000). A new mtDNA mutation associated with a progressive encephalopathy and cytochrome c oxidase deficiency. Neurology *54*, 1693–1696.

Stewart, J.B., Freyer, C., Elson, J.L., Wredenberg, A., Cansu, Z., Trifunovic, A., and Larsson, N.-G. (2008). Strong purifying

selection in transmission of mammalian mitochondrial DNA. PLoS Biol. *6*, e10.

Stoddard-Bennett, T., and Pera, R.R. (2020). Stem cell therapy for Parkinson's disease: safety and modeling. Neural Regen. Res. *15*, 36–40.

Strobbe, D., Caporali, L., Iommarini, L., Maresca, A., Montopoli, M., Martinuzzi, A., Achilli, A., Olivieri, A., Torroni, A., Carelli, V., et al. (2018). Haplogroup J mitogenomes are the most sensitive to the pesticide rotenone: relevance for human diseases. Neurobiol. Dis. *114*, 129–139.

Suomalainen, A., Majander, A., Pihko, H., Peltonen, L., and Syvänen, A.C. (1993). Quantification of tRNA3243(Leu) point mutation of mitochondrial DNA in MELAS patients and its effects on mitochondrial transcription. Hum. Mol. Genet. *2*, 525–534.

Torroni, A., Petrozzi, M., D'Urbano, L., Sellitto, D., Zeviani, M., Carrara, F., Carducci, C., Leuzzi, V., Carelli, V., Barboni, P., et al. (1997). Haplotype and phylogenetic analyses suggest that one European-specific mtDNA background plays a role in the expression of Leber hereditary optic neuropathy by increasing the penetrance of the primary mutations 11778 and 14484. Am. J. Hum. Genet. *60*, 1107–1121.

Volpato, V., and Webber, C. (2020). Addressing variability in iPSC-derived models of human disease: guidelines to promote reproducibility. Dis. Model. Mech. *13*, dmm042317.

Wallace, D.C. (2015). Mitochondrial DNA variation in human radiation and disease. Cell *163*, 33–38.

Weerasinghe, C.A.L., Bui, B.-H.T., Vu, T.T., Nguyen, H.-L.T., Phung, B.-K., Nguyen, V.-M., Pham, V.-A., Cao, V.-H., and Phan, T.-N. (2018). Leigh syndrome T8993C mitochondrial DNA mutation: heteroplasmy and the first clinical presentation in a Vietnamese family. Mol. Med. Rep. *17*, 6919–6925.

Wei, W., Tuna, S., Keogh, M.J., Smith, K.R., Aitman, T.J., Beales, P.L., Bennett, D.L., Gale, D.P., Bitner-Glindzicz, M.A.K., Black, G.C., et al. (2019). Germline selection shapes human mitochondrial DNA diversity. Science *364*, eaau6520.

Xu, X., Duan, S., Yi, F., Ocampo, A., Liu, G.-H., and Izpisua Belmonte, J.C. (2013). Mitochondrial regulation in pluripotent stem cells. Cell Metab. *18*, 325–332.

Zambelli, F., Mertens, J., Dziedzicka, D., Sterckx, J., Markouli, C., Keller, A., Tropel, P., Jung, L., Viville, S., Van de Velde, H., et al. (2018). Random mutagenesis, clonal events, and embryonic or somatic origin determine the mtDNA variant type and load in human pluripotent stem cells. Stem Cell Reports *11*, 102–114.

Stem Cell Reports, Volume *16*

Supplemental Information

The relevance of mitochondrial DNA variants fluctuation during reprogramming and neuronal differentiation of human iPSCs

Flavia Palombo, Camille Peron, Leonardo Caporali, Angelo Iannielli, Alessandra Maresca, Ivano Di Meo, Claudio Fiorini, Alice Segnali, Francesca L. Sciacca, Ambra Rizzo, Sonia Levi, Anu Suomalainen, Alessandro Prigione, Vania Broccoli, Valerio Carelli, and Valeria Tiranti

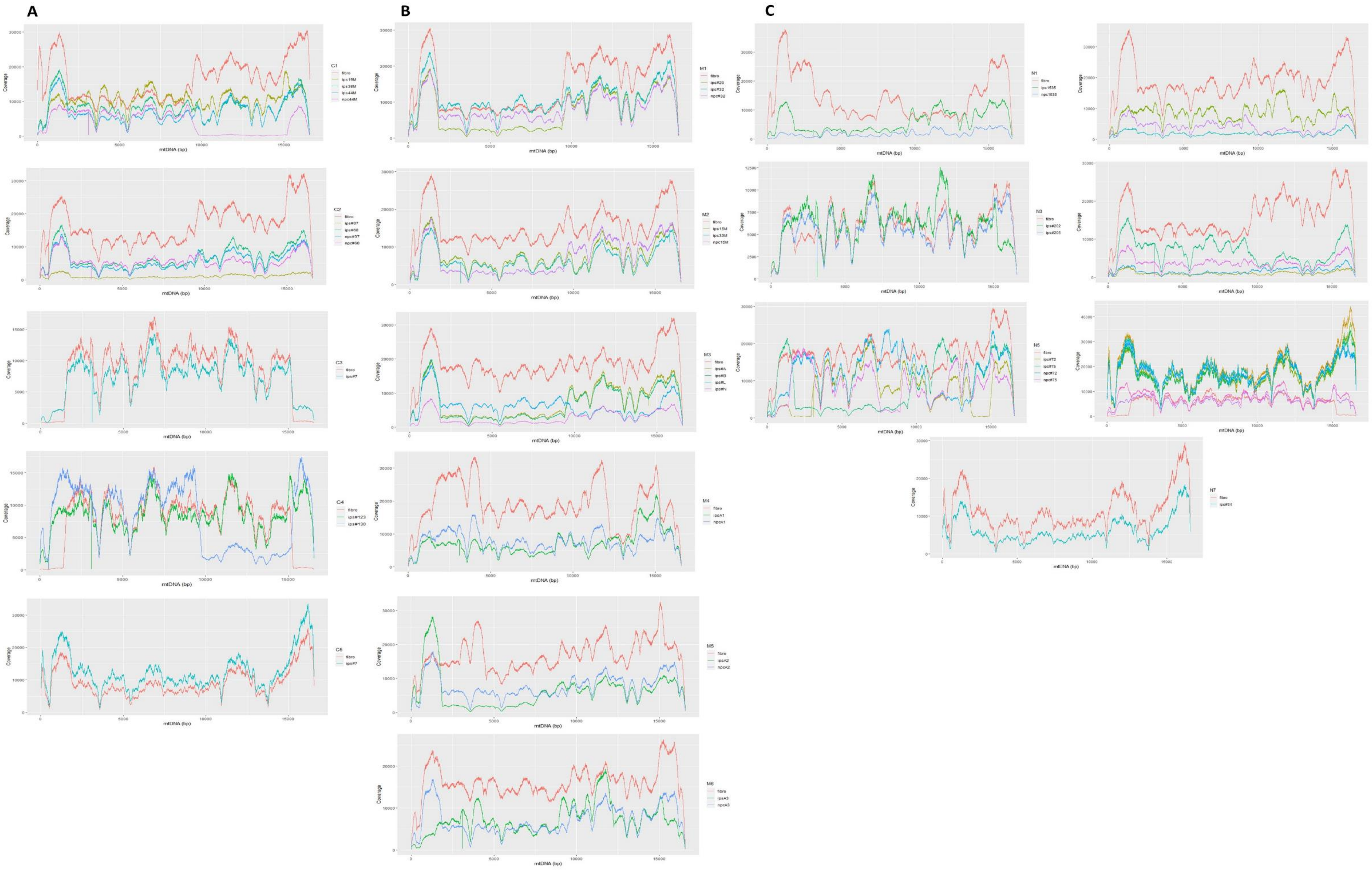

Figure S1. mtDNA NGS coverage in CONTROL (A), MITO (B) and NUCLEAR (C) group.

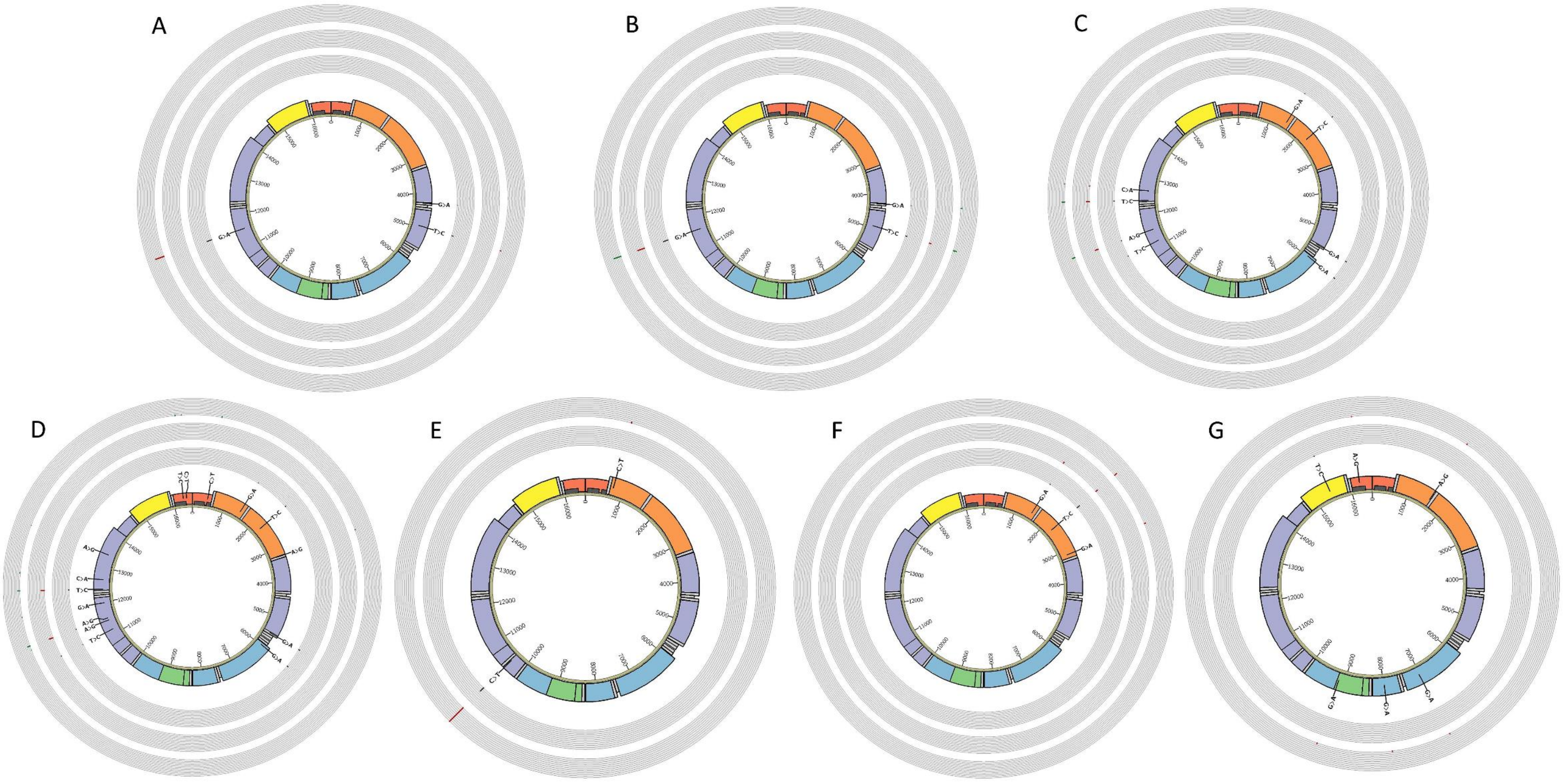

Figure S2. Circos plot of the structure of the human mitochondrial genome showing CONTROL heteroplasmic variants. Colored boxes represent: MT-DLOOP (red), transfer RNAs (light gray), ribosomal RNAs (orange), Complex I genes (purple), Complex III gene (yellow), Complex IV genes (blue) and Complex V genes (green). Shorter boxes are antisense transcripts, while others are sense transcripts. Dark gray bars represent hypervariable regions. The innermost circle with black lines showed fibroblasts heteroplasmic variants, the outer circles with red lines showed iPSCs heteroplasmic variants and the outermost circle with green lines showed NPCs heteroplasmic variants; the length of the lines are proportionate to variants heteroplasmic loads. A. C1 fibroblasts, 19M and 38M iPSC clones. B. C1 fibroblasts, 44M iPSC and NPC clones. C. C2 fibroblasts, #37 iPSC and NPC clones. D. C2 fibroblasts, #68 iPSC and NPC clones. E. C3 fibroblasts and #7 iPSC clone. F. C4 fibroblasts, #123 and #130 iPSC clones. G. C5 fibroblasts and #105 iPSC clone.

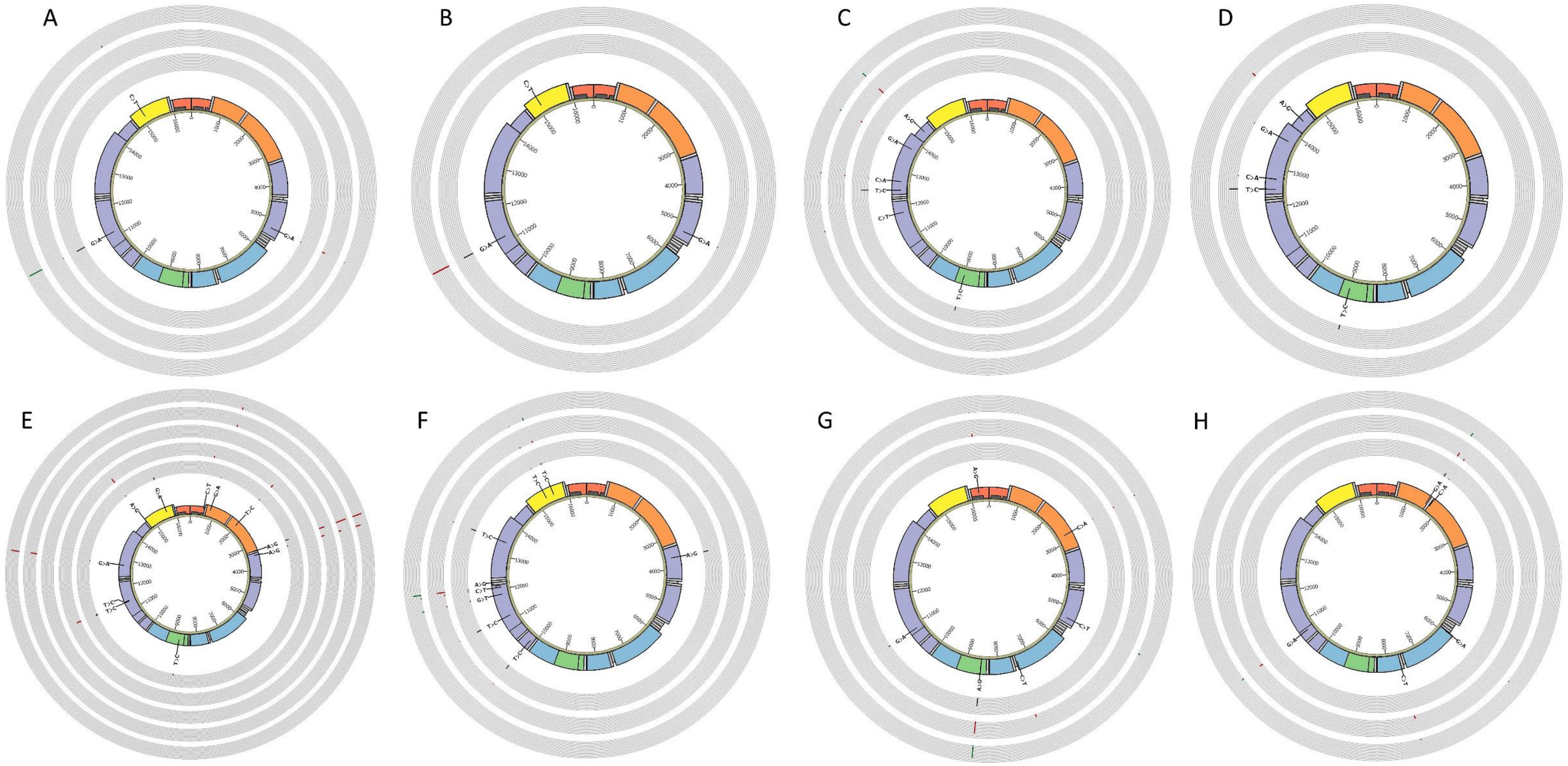

Figure S3. Circos plot of the structure of the human mitochondrial genome showing MITO heteroplasmic variants. A. M1 fibroblasts, #20 iPSC and NPC clones. B. M1 fibroblasts and #32 iPSC clone. C. M2 fibroblasts, 15M iPSC and NPC clones. D. M2 fibroblasts and 33M iPSC clone. E. M3 fibroblasts, #A, #B, #L and #N iPSC clone. F. M4 fibroblasts, A1 iPSCs and NPC clones. G. M5 fibroblasts, A2 iPSC and NPC clones. H. M6 fibroblasts, A3 iPSC and NPC clones.

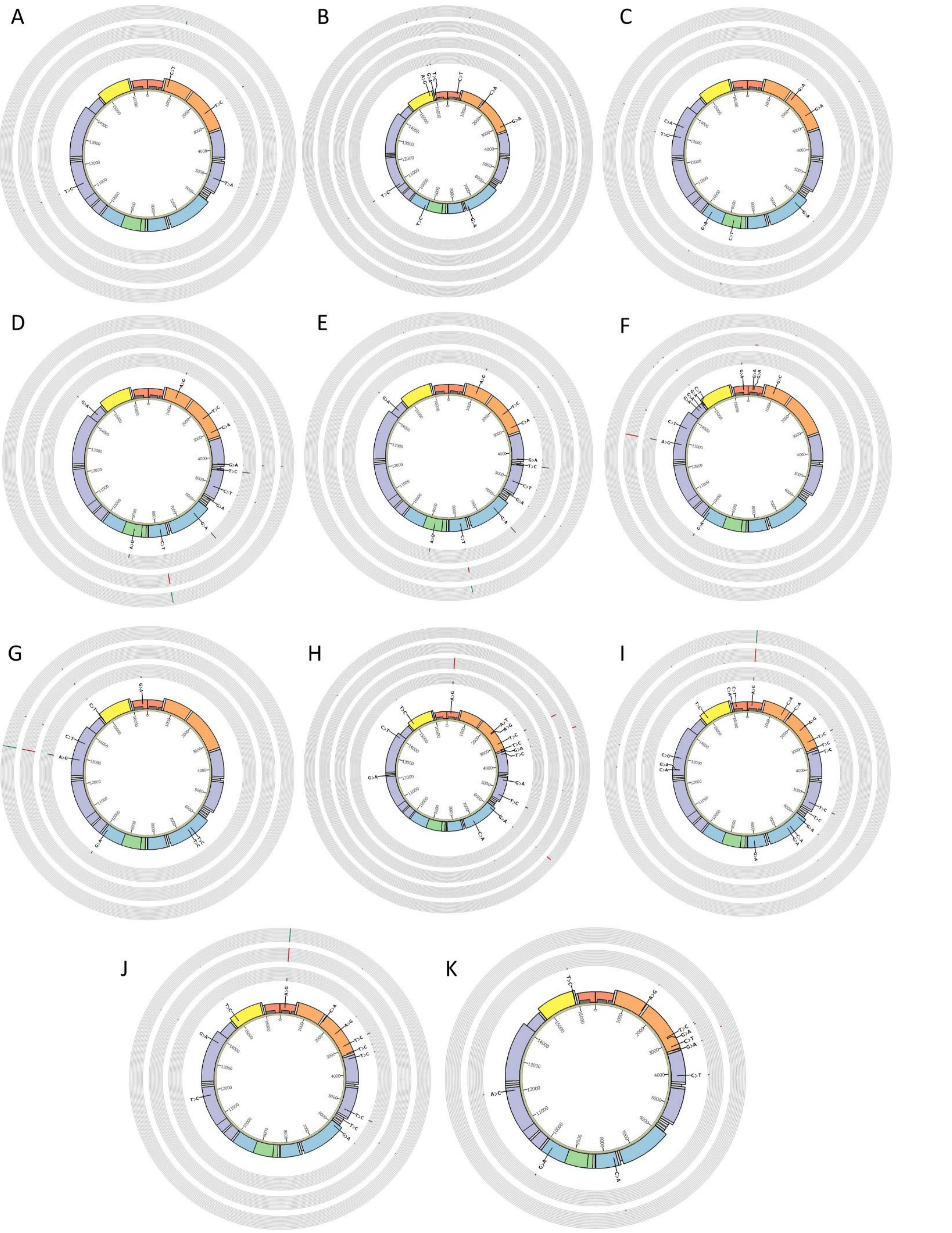

Figure S4. Circos plot of the structure of the human mitochondrial genome showing NUCLEAR heteroplasmic variants. A. N1 fibroblasts, 1535 iPSC and NPC clones. B. N2 fibroblasts, 13M iPSC and NPC clones. C. N3 fibroblasts, #202 and #205 iPSC clones. D. N4 fibroblasts, #12 iPSC and NPC clones. E. N4 fibroblasts, #18 iPSC and NPC clones. F. N5 fibroblasts, #72 iPSC and NPC clones. G. N5 fibroblasts, #75 iPSC and NPC clones. H. N6 fibroblasts, #2, #3 and #6 iPSC clones. I. N6 fibroblasts, #4 iPSC and NPC clones. J. N6 fibroblasts, #8 iPSC and NPC clones. K. N7 fibroblasts and #34 iPSC clone

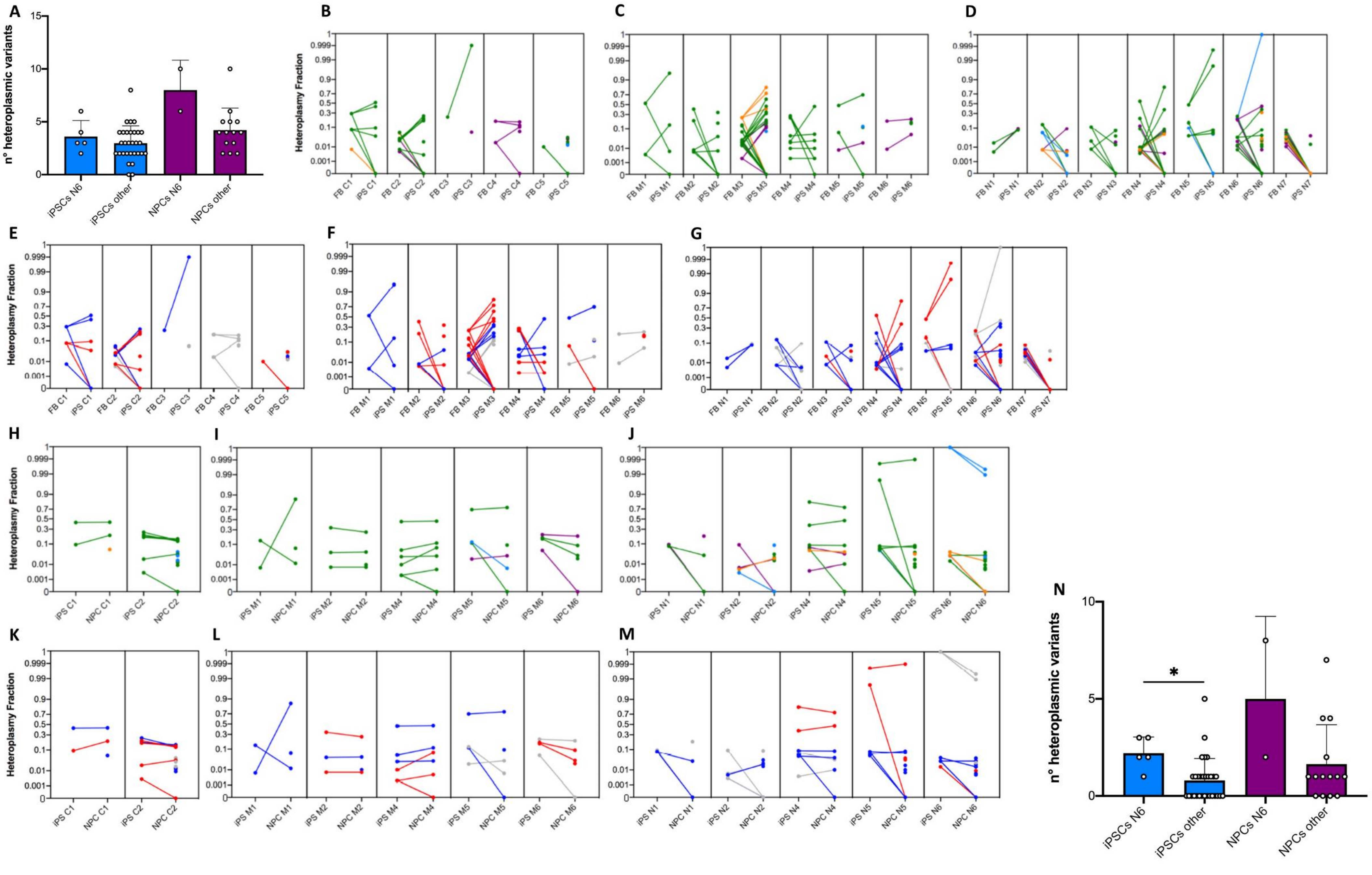
A
n° heteroplasmic variants
iPSCs N6
iPSCs other
NPCs N6
NPCs other
B
C
D
E
F
G
H
I
J
K
L
M
Heteroplasmy Fraction
FB C1
iPS C1
FB C2
iPS C2
FB C3
iPS C3
FB C4
iPS C4
FB C5
iPS C5
FB M1
iPS M1
FB M2
iPS M2
FB M3
iPS M3
FB M4
iPS M4
FB M5
iPS M5
FB M6
iPS M6
FB N1
iPS N1
FB N2
iPS N2
FB N3
iPS N3
FB N4
iPS N4
FB N5
iPS N5
FB N6
iPS N6
FB N7
iPS N7
NPC C1
NPC C2
NPC M1
NPC M2
NPC M4
NPC M5
NPC M6
NPC N1
NPC N2
NPC N4
NPC N5
NPC N6
N
*

Figure S5. Analysis and characterization of heteroplasmic variants. A. iPSC and NPC clones obtained from the N6 patient carrying the *POLG* p.P648R mutation did not display a significantly higher number of heteroplasmic variants compared to the iPSCs (p=0.417) and the NPCs (p=0.06) derived from controls and from the MITO or NUCLEAR groups. B-D. Variants HF fluctuations during the reprogramming step from fibroblasts to iPSCs in CONTROL (B), MITO (C) and NUCLEAR (D) groups. Dots appearing in iPSC means unique variants. Non transmitted variants are highlighted with a straight line going to zero in iPSC. blue= MT-DLOOP violet=rRNA orange=tRNA green=coding genes. E-G. Variants HF fluctuations during the reprogramming step from fibroblasts to iPSCs in CONTROL (E), MITO (F) and NUCLEAR (G) groups. Dots appearing in iPSC means unique variants. Non transmitted variants are highlighted with a straight line going to zero in iPSC. blue=benign red=damaging gray=no prediction. H-J. Variants HF fluctuations during differentiation step from iPSCs to NPCs in CONTROL (H) MITO (I) and NUCLEAR (J) groups. Dots appearing in NPC means unique variants. Non transmitted variants are highlighted with a straight line going to zero in NPC. blue= MT-DLOOP violet=rRNA orange=tRNA green=coding genes. K-M. Variants HF fluctuations during differentiation step from iPSCs to NPCs in CONTROL (K), MITO (L) and NUCLEAR (M) groups. Dots appearing in NPC means unique variants. Non transmitted variants are highlighted with a straight line going to zero in NPC. blue=benign; red=damaging; gray=no prediction. N. iPSC clones obtained from the N6 patient carrying the *POLG* p.P648R mutation accumulated a significantly higher number of unique variants compared to all other iPSCs (p=0.003), differently from the NPC clones (p=0.09).

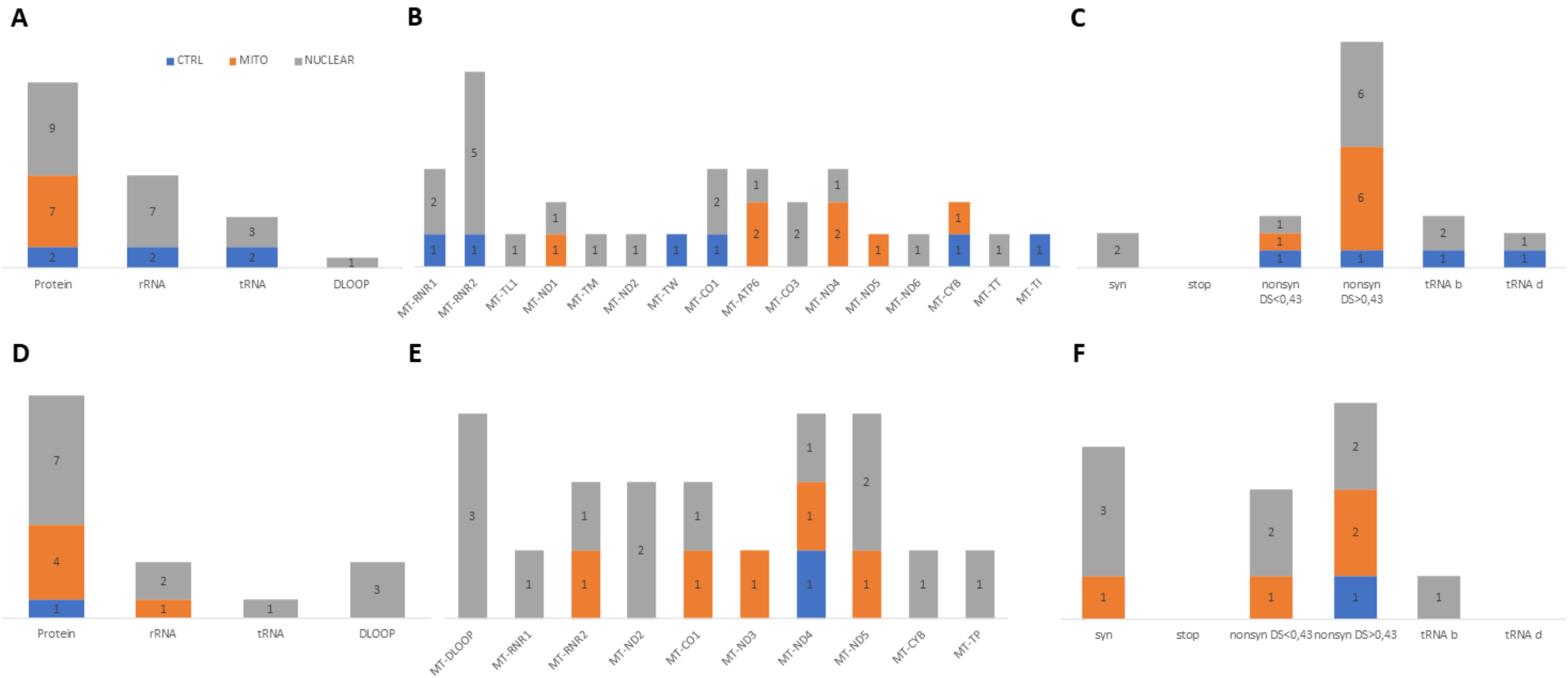

Figure S6. mtDNA localization and prediction of variants non-transmitted in reprogramming and differentiating steps in the three groups. A. Thirty-two heteroplasmic variants were non-transmitted to the iPSCs clones during the reprogramming step. B. Mitochondrial genes localization of non-transmitted variants in iPSCs. C. Predictions of non-transmitted variants in iPSCs. D. Sixteen heteroplasmic variants were non-transmitted to the NPCs clones in the differentiating step. E. Mitochondrial genes localization of non-transmitted variants in NPCs. F. Predictions of non-transmitted variants in NPCs. syn=synonymous; nonsyn=nonsynonymous; b=benign; d=damaging

Fibroblasts	Mean Coverage	% of bases above 5000X	iPSC clones	Mean Coverage	% of bases above 5000X	NPC clones mean coverage	% of bases above 5000X
**C1**	16,215 X	100	19M	10.433 X	94.4		
			38M	8,331 X	84.5		
			44M	7,055 X	72.7	4,057 X	51
**C2**	16,559 X	100	#37	1,087 X	0	5,531 X	47.1
			#68	6,653 X	56.1	5,534 X	60
**C3**	7,641 X	80.5	#7	7,274 X	76.5		
**C4**	7,874 X	78.7	#123	8,437 X	90		
			#130	8,220 X	61.5		
**C5**	7,740 X	89.5	#105	9,135 X	96.3		
**M1**	14,751 X	100	#20	7,282 X	49.2	7,231 X	82
			#32	8,798 X	94.2		
**M2**	15,206 X	100	15M	6,785 X	75.4	6,230 X	50
			33M	6,095 X	65.5		
**M3**	18,298 X	100	#A	6,171 X	49.2		
			#B	5,923 X	48.4		
			#L	5,904 X	63.2		
			#N	2,726 X	16		
**M4**	18,917 X	100	A1	7,189 X	72.8	7,417 X	85.3
**M5**	7,537 X	68.5	A2	6,271 X	56.3	7,002 X	82
**M6**	16,034 X	100	A3	7,138 X	74.2	6,618 X	70.8
**N1**	14,648 X	100	1535	5,970 X	45.6	1,913 X	0
**N2**	19,002 X	100	13M	8,458 X	90.4	4,074 X	27.6
			20M	1,857 X	0		
**N3**	6,453 X	68.3	#202	6,238 X	75.3		
			#205	5,711 X	67.4		
**N4**	15,839 X	100	#12	1,136 X	0	1,655 X	0
			#18	6,857 X	67.2	3,776 X	13.7
**N5**	17,048 X	100	#72	7,793 X	66.6	12,425 X	85.2
			#75	8,388 X	49.8	7,232 X	75.7
**N6**	5,698 X	89.9	#2	15,998 X	98.3		

			#3	14,770 X	97.9		
			#4	11,107 X	94.2	6,114 X	77
			#6	11,723 X	95.7		
			#8	13,095 X	97.3	7,229 X	86.3
**N7**	10,640 X	96.3	#34	5,606 X	49.5		

Table S1. mtDNA NGS coverage.

POSITION	REF	ALT	LOCUS	AA CHANGE	tRNA	MitoTIP	DISEASE SCORE	POLYPHEN2	WHO
183	A	G	MT-DLOOP						N6
811	G	A	MT-RNR1						M3
1489	G	A	MT-RNR1						C4
1562	G	A	MT-RNR1						M6
1693	C	A	MT-RNR2						N2
1693	C	A	MT-RNR2						M6
1975	T	C	MT-RNR2						M3
2256	T	C	MT-RNR2						C4
2404	T	C	MT-RNR2						N4
2522	T	C	MT-RNR2						N1
2766	C	A	MT-RNR2						M5
2895	T	C	MT-RNR2						N6
3009	C	A	MT-RNR2						N4
3243	A	G	MT-TL1		14;0;DL;A;Y	pathogenic			M3
3399	A	G	MT-ND1	Syn					M3
3404	T	C	MT-ND1	L33P			0.84	0.991	N6
4244	G	A	MT-ND1	S313N			0.11	0.0	N4
4933	T	C	MT-ND2	L155P			0.857	0.99	C1
5068	T	A	MT-ND2	M200K			0.417	0.184	N1
5107	C	T	MT-ND2	T213I			0.244	0.101	N4
5293	G	A	MT-ND2	S275N			0.189	0	M1
5610	G	A	MT-TA		50;II;TS;C;N	possibly benign			N4
6018	G	A	MT-CO1	A39T			0.14	0.015	N6
7824	C	T	MT-CO2	S80F			0.835	0.913	N4
8537	A	G	MT-ATP8	I58V			0.341	0.326	M5
10158	T	C	MT-ND3	S34P			0.397	0.202	M4
10377	C	T	MT-ND3	Syn					C3
10861	T	C	MT-ND4	Syn					N2
11150	G	A	MT-ND4	A131T			0.161	0.035	M1
11154	T	C	MT-ND4	I132T			0.72	0.99	C2
11157	T	C	MT-ND4	I133T			0.794	0.99	M4
11299	T	C	MT-ND4	Syn					N1
11361	T	C	MT-ND4	M201T			0.709	0.949	M3

11511	A	G	MT-ND4	N251S			0.153	0	C2
11546	G	A	MT-ND4	V263M			0.137	0	C1
11835	G	T	MT-ND4	W359L			0.73	0.995	M4
12049	C	T	MT-ND4	Syn					M4
12100	A	G	MT-ND4	Syn					M4
12371	T	C	MT-ND5	L12P			O.502	na	C2
12721	C	A	MT-ND5	L129M			0.62	0.965	M2
12778	G	A	MT-ND5	Stop-gain					M3
12926	A	G	MT-ND5	D197G			0.485	0.65	N5
13020	A	C	MT-ND5	Syn					N3
13424	T	C	MT-ND5	L363P			0.857	0.995	M4
13425	C	A	MT-ND5	Syn					N3
13759	G	A	MT-ND5	A475P			0.08	0	M2
14750	A	G	MT-CYB	T2A			0.051	0	M3
14761	C	T	MT-CYB	Syn					N5
15293	T	C	MT-CYB	F183L			0.74	0.999	M4
15541	T	C	MT-CYB	Syn					M4
15959	G	A	MT-TP		70;II;AS;C;N	possibly benign			N2
16044	T	C	MT-DLOOP						N2

Table S2. Variants transmitted from fibroblasts to iPSCs clones.

POSITION	REF	ALT	LOCUS	AA CHANGE	tRNA	MitoTIP	DISEASE SCORE	POLYPHEN2	WHO
183	A	G	MT-DLOOP						N6
1562	G	A	MT-RNR1						M6
2404	T	C	MT-RNR2						N4
2766	C	A	MT-RNR2						M5
3009	C	A	MT-RNR2						N4
3010	G	A	MT-RNR2						N2
4244	G	A	MT-ND1	S313N			0.11	0	N4
4933	T	C	MT-ND2	L155P			0.857	0.99	C1
5293	G	A	MT-ND2	S275N			0.189	0	M1
5610	G	A	MT-TA		50;II;TS;C;N	possibly benign			N4
5654	T	C	MT-TA		2;II;AS;A;N	possibly benign			N6
7592	C	T	MT-CO2	H3Y			0.718	0.991	M6
7824	C	T	MT-CO2	S80F			0.835	0.913	N4
8537	A	G	MT-ATP8	I58V			0.341	0.326	M5
10695	G	A	MT-ND4L	A76T			0.74	0.931	M6
11150	G	A	MT-ND4	A131T			0.161	0.035	M1
11299	T	C	MT-ND4	Syn					N1
11511	A	G	MT-ND4	N251S			0.153	0	C2
11546	G	A	MT-ND4	V263M			0.137	0	C1
11835	G	T	MT-ND4	W359L			0.73	0.995	M4
12049	C	T	MT-ND4	Syn					M4
12100	A	G	MT-ND4	Syn					M4
12371	T	C	MT-ND5	L12P			0.502	No Info	C2
12649	C	A	MT-ND5	L105M			0.67	1	C2
12721	C	A	MT-ND5	L129M			0.62	0.965	M2
12926	A	G	MT-ND5	D197G			0.485	0.65	N5
13575	C	T	MT-ND5	Syn					N5
13759	G	A	MT-ND5	A475T			0.08	0	M2

13813	G	A	MT-ND5	V493I			0.14	0.002	N6
14424	A	G	MT-ND6	S84P			0.832	1	M2
14761	C	T	MT-CYB	Syn					N5
15293	T	C	MT-CYB	F183L			0.74	0.999	M4
15541	T	C	MT-CYB	Syn					M4
15959	G	A	MT-TP		70;II;AS;C;N	possibly benign			N2
16254	A	G	MT-DLOOP						M5

Table S3. Variants transmitted from iPSCs to NPCs clones.

POSITION	REF	ALT	HF	LOCUS	AA CHANGE	tRNA	MitoTIP	DISEASE SCORE	POLYPHEN2	WHO	GB
185	G	A	6%	MT-DLOOP						N5 #72	3445
228	G	A	5%	MT-DLOOP						N5 #72	1901
537	C	T	8%	MT-DLOOP						M3 A	114
722	C	T	6%	MT-RNR1						C3 #7	62
1388	C	A	2.9%	MT-RNR1						N6 #4	NA
1573	A	G	2.9%	MT-RNR1						C5 #105	NA
2297	A	T	0.7%	MT-RNR2						N6 #3	NA
2643	G	A	2%	MT-RNR2						N3 #205	2
3010	G	A	0.6%	MT-RNR2						N2 13M	7223
3110	C	T	4.3%	MT-RNR2						N7 #34	10
3123	G	A	7%	MT-RNR2						C4 #130	NA
3281	G	A	32%	MT-TL1		50;0;TS;G;N	possibly benign			N6 #6	2
4596	G	A	1.4%	MT-ND2	V43I			0.12	0.006	N6 #3	NA
5654	T	C	4.4%	MT-TA		2;II;AS;A;N	possibly benign			N6 #8	5
6486	C	A	15%	MT-CO1	L195I			0.73	0.99	N6 #4	NA
7002	C	A	1.1%	MT-CO1	L367M			0.75	0.999	N6 #2	NA
7026	G	A	1.5%	MT-CO1	A375T			0.87	1.0	C5 #105	NA
7412	C	T	11%	MT-CO1	syn					M5 A2	11
7592	C	T	16%	MT-CO2	H3Y			0.718	0.991	M6 A3	1
7687	C	A	1.5%	MT-CO2	I34M			0.77	0.969	N7 #34	NA
7970	T	A	2%	MT-CO2	E129K			0.13	0.0	C5 #105	NA
8815	C	T	8%	MT-ATP6	Stop-gain					N3 #205	NA
9163	G	A	3.4%	MT-ATP6	V213I			0.81	0.975	C5 #105	58
9715	G	A	4%	MT-CO3	G170D			0.842	0.999	N3 #202	NA
10695	G	A	17%	MT-ND4L	A76T			0.74	0.931	M6 A3	2
12192	G	A	2.2%	MT-TH		59;II;TL;G;N	likely benign			N6 #3	112
12649	C	A	2%	MT-ND5	L105M			0.67	1.0	C2 #37	NA

13575	C	T	8%<br>7%	MT-ND5	syn					N5 #72<br>N5 #75	24
13813	G	A	3%	MT-ND5	V493I			0.14	0.002	N6 #8	22
14004	C	T	2.5%	MT-ND5	syn					N6 #6	7
14424	A	G	34%<br>16%	MT-ND6	S84P			0.832	1.0	M2 #15<br>M2 #33	1
16019	C	A	3%	MT-TP		5;II;AS;G;N	possibly benign			N6 #4	NA
16240	A	G	1.3%	MT-DLOOP						C5 #105	358
16254	A	G	12%	MT-DLOOP						M5 A2	229

Table S4. ***Unique*** **variants of iPSCs clones.**

POSITION	REF	ALT	HF	LOCUS	AA CHANGE	tRNA	MitoTIP	DISEASE SCORE	POLYPHEN2	WHO	GB
438	C	T	8.8%	MT-DLOOP						N2 13M	15
456	C	T	4.6%	MT-DLOOP						C2 #68	1911
765	C	T	18.9%	MT-RNR1						N1 1535	NA
991	G	C	1%	MT-RNR1						N5 #72	NA
1693	C	A	2.8%<br>1.9%	MT-RNR2						N6 #4<br>N6 #8	NA
3243	A	G	1.2%	MT-TL1		14;;DL;A;Y	confirmed pathogenic			C2 #68	9
5366	C	T	9.4%	MT-ND2	syn					M5 A2	3
5917	G	A	2.2%	MT-CO1	R5H			0.76	0.999	M6 A3	NA
6512	T	C	1%	MT-CO1	syn					N5 #75	8
6610	G	A	3.1%	MT-CO1	Stop-gain					N6 #4	NA
6641	T	C	0.7%	MT-CO1	syn					N5 #75	40
7337	G	A	2.3%	MT-CO1	syn					N2 13M	589
8007	G	A	0.9%	MT-CO2	R141Q			0.8	1.0	N6 #4	NA
9311	T	C	1.6%	MT-CO3	syn					N2 13M	47
11443	A	G	0.9%	MT-ND4	syn					C2 #68	4
11788	C	T	1%	MT-ND4	syn					M2 15M	33
11827	T	C	0.5%	MT-ND4	syn					N6 #8	80
11969	G	A	1.5%	MT-ND4	A404T			0.26	0.002	C2 #68	742
12549	C	A	2%	MT-ND5	syn					N6 #4	NA
12561	G	A	1.5%	MT-ND5	syn					N6 #4	230
13011	C	G	4.2%	MT-ND5	syn					N6 #4	NA
13395	A	G	1.3%	MT-ND5	syn					C2 #68	337
14438	G	A	4.2%	MT-ND6	P79L			0.91	1.0	N5 #72	NA
14560	G	A	1.8%	MT-ND6	syn					N5 #72	1140
14710	G	A	3.5%	MT-TE		36;II;CL;C;N	confirmed pathogenic			N5 #72	NA *Encephalomyopathy + Retinopathy

15100	C	T	7%	MT-CYB	syn					M1 #20	16
15643	C	T	1%	MT-CYB	syn					N4 #18	9
15758	A	G	3.4%	MT-CYB	I338V			0.15	0.001	N2 13M	389
16148	C	T	2.5%	MT-DLOOP						N6 #4	3350
16304	T	C	3%	MT-DLOOP						C2 #68	11367
16400	C	T	1.7%	MT-DLOOP						C2 #68	322

Table S5. *Unique* variants in NPCs clones.

POSITION	REF	ALT	HF	LOCUS	AA CHANGE	tRNA	MitoTIP	DISEASE SCORE	POLYPHEN2	WHO	GB
1082	A	G	12%	MT-RNR1						N4	NA
1489	G	A	0.5%	MT-RNR1						C2	NA
1578	A	G	1.1%	MT-RNR1						N7	NA
1816	G	A	2.3%	MT-RNR2						N3	1
2256	T	C	2.6%	MT-RNR2						C2	NA
2338	A	G	1.2%	MT-RNR2						N6	NA
2778	T	C	1.8%	MT-RNR2						N7	NA
2819	G	A	6.1%	MT-RNR2						N7	NA
3193	T	C	2.2%	MT-RNR2						N6	NA
3248	G	A	2.4%	MT-TL1		18;0;DL;G;Y	possibly benign			N7	1
3628	A	G	25.3%	MT-ND1	T108A			0.135	0.001	M4	NA
4059	C	T	4.9%	MT-ND1	syn					N7	57
4445	T	C	55.4%	MT-TM		48;II;VL;U;Y	possibly benign			N4	NA
5296	T	C	25.1%	MT-ND2	L276P			0.69	0.988	N6	NA
5540	G	A	5.6%	MT-TW		30;II;CS;G;N0.65	possibly pathogenic			C2	NA *Encephalomyopathy/ DEAF
5970	G	A	6%	MT-CO1	G23S			0.26	0.04	C2	3
6261	G	A	2.3%	MT-CO1	A120T			0.664	0.988	N3	361
6382	G	A	55.2%	MT-CO1	G160E			0.869	0.992	N4	NA
8728	T	C	7.4%	MT-ATP6	W68R			0.874	0.999	M3	NA
8817	A	G	21.5%	MT-ATP6	syn					N4	54
8993	T	C	19.6%	MT-ATP6	L156P			0.902	0.999	M2	2 *NARP/Leigh Disease /MILS /other
9753	G	A	8%	MT-CO3	E183K			0.76	0.983	N7	1
9906	G	A	15.7%	MT-CO3	G234S			0.758	1.0	N5	NA
10863	G	A	6.9%	MT-ND4	S35N			0.762	0.995	M5	3
11340	T	C	12.9%	MT-ND4	L194S			0.699	0.978	M3	NA
12040	A	C	3.2%	MT-ND4	K427N			0.7	0.994	N7	11
12460	T	C	40.5%	MT-ND5	S42P			0.711	0.978	M2	NA
14384	G	A	9.1%	MT-ND6	A97V			0.073	0.0	N4	83

15441	T	C	1%	MT-CYB	L232S			0.66	0.981	C5	NA
15612	G	A	13.9%	MT-CYB	G289E			0.844	1.0	M3	NA
15898	T	C	3.8%	MT-TT		11;II;DS;U;N	possibly pathogenic			N7	1
16390	G	A	9.7%	MT-DLOOP						N5	7404

Table S6. Variants non-transmitted from fibroblasts to iPSCs.

POSITION	REF	ALT	HF	LOCUS	AA_CHANGE	tRNA	MitoTIP	DISEASE SCORE	POLYPHEN2	WHO	GB
**185**	G	A	5.5%	MT-DLOOP						N5 #72	3445
**228**	G	A	5.2%	MT-DLOOP						N5 #72	1901
**1388**	C	A	2.9%	MT-RNR1						N6 #4	NA
**1693**	C	A	5.6%<br>9%	MT-RNR2						M6 A3;<br>N2 13M	NA
**2522**	T	C	9%	MT-RNR2						N1 1535	NA
**5068**	T	A	8%	MT-ND2	M200K			0.417	0.184	N1 1535	NA
**5107**	C	T	6%	MT-ND2	T213I			0.244	0.101	N4 #12	NA
**6486**	C	A	1.5%	MT-CO1	L195I			0.73	0.99	N6 #4	NA
**7412**	C	T	11.3%	MT-CO1	syn					M5 A2	11
**10158**	T	C	0.2%	MT-ND3	S34P			0.397	0.202	M4 A1	NA
**10861**	T	C	0.3%	MT-ND4	syn					N2 13M	42
**11154**	T	C	0.3%	MT-ND4	I132T			0.72	0.99	C4 #68	NA
**11157**	T	C	0.2%	MT-ND4	I133T			0.794	0.99	M4 A1	2
**13424**	T	C	0.2%	MT-ND5	L363P			0.857	0.995	M4 A1	NA
**16019**	C	A	3%	MT-TP		5;II;AS;G;N	possibly benign			N6 #4	NA
**16044**	T	C	0.3%	MT-DLOOP						N2 13M	5

Table S7. Variants non-transmitted from iPSCs to NPCs.

POSITION	REF	ALT	HF	WHO iPSC	DEPTH	FORWARD	REVERSE
2297	A	T	0.7%	N6 #3	16359	7985	8374
2643	G	A	2%	N3 #205	5774	2805	2969
3010	G	A	0.6%	N2 13M	10821	5220	5601
4596	G	A	1.4%	N6 #3	12618	6066	6552
7002	C	A	1.1%	N6 #2	17465	7040	10425
7026	G	A	1.5%	C5 #105	12452	5048	7404
7687	C	A	1.5%	N7 #34	3615	1605	2010
7970	T	A	2%	C5 #105	9320	5279	4041
12649	C	A	2%	C2 #37	1237	571	666
**POSITION**	**REF**	**ALT**	**HF**	**WHO NPC**	**DEPTH**	**FORWARD**	**REVERSE**
991	G	C	1%	N5 #72	5772	3086	2686
1693	C	A	1.9%	N6 #6-#8	8154	4812	3342
3243	A	G	1.2%	C2 #68	4260	1957	2303
6512	T	C	1%	N5 #75	11985	6789	5196
6641	T	C	0.7%	N5 #75	13436	7282	6154
8007	G	A	0.9%	N6 #4	6026	2920	3106
9311	T	C	1.6%	N2 13M	5891	2603	3288
11443	A	G	0.9%	C2 #68	5953	3028	2925
11788	C	T	1%	M2 15M	14260	6871	7389
11827	T	C	0.5%	N6 #8	11247	5127	6120
11969	G	A	1.5%	C2 #68	5185	2447	2738
12549	C	A	2%	N6 #4	5804	2903	2901
12561	G	A	1.5%	N6 #4	5514	2595	2919
13395	A	G	1.3%	C2 #68	4672	2233	2439
14560	G	A	1.8%	N5 #72	10719	5947	4772
15643	C	T	1%	N4 #18	6378	3380	2998
16400	C	T	1.7%	C2 #68	11278	6161	5117

Table S8. Coverage and reads strand orientation of *unique* variants with HF≤2% in iPSCs and NPCs.
